# Multiple Roles of Apolipoprotein E4 in Oxidative Lipid Metabolism and Ferroptosis During the Pathogenesis of Alzheimer’s Disease

**DOI:** 10.1007/s12031-024-02224-4

**Published:** 2024-07-03

**Authors:** Parisa Faraji, Hartmut Kühn, Shahin Ahmadian

**Affiliations:** 1 Institute of Biochemistry and Biophysics, University of Tehran, Tehran, Iran; 2 Department of Biochemistry, Charité-Universitätsmedizin Berlin, Corporate Member of Freie Universität Berlin and Humboldt Universität zu Berlin, Charitéplatz 1, 10117 Berlin, Germany

**Keywords:** Neurodegeneration, Proteostasis, Lipid peroxidation, Iron homeostasis, Glutathione peroxidase 4, Free radicals

## Abstract

Alzheimer’s disease (AD) is the most prevalent neurodegenerative disease worldwide and has a great socio-economic impact. Modified oxidative lipid metabolism and dysregulated iron homeostasis have been implicated in the pathogenesis of this disorder, but the detailed pathophysiological mechanisms still remain unclear. Apolipoprotein E (APOE) is a lipid-binding protein that occurs in large quantities in human blood plasma, and a polymorphism of the *APOE* gene locus has been identified as risk factors for AD. The human genome involves three major APOE alleles (*APOE2*, *APOE3*, *APOE4*), which encode for three subtly distinct apolipoprotein E isoforms (APOE2, APOE3, APOE4). The canonic function of these apolipoproteins is lipid transport in blood and brain, but *APOE4* allele carriers have a much higher risk for AD. In fact, about 60% of clinically diagnosed AD patients carry at least one *APOE4* allele in their genomes. Although the APOE4 protein has been implicated in pathophysiological key processes of AD, such as extracellular beta-amyloid (Aβ) aggregation, mitochondrial dysfunction, neuroinflammation, formation of neurofibrillary tangles, modified oxidative lipid metabolism, and ferroptotic cell death, the underlying molecular mechanisms are still not well understood. As for all mammalian cells, iron plays a crucial role in neuronal functions and dysregulation of iron homeostasis has also been implicated in the pathogenesis of AD. Imbalances in iron homeostasis and impairment of the hydroperoxy lipid-reducing capacity induce cellular dysfunction leading to neuronal ferroptosis. In this review, we summarize the current knowledge on *APOE4*-related oxidative lipid metabolism and the potential role of ferroptosis in the pathogenesis of AD. Pharmacological interference with these processes might offer innovative strategies for therapeutic interventions.

**Supplementary Information:**

The online version contains supplementary material available at 10.1007/s12031-024-02224-4.

## Background

Alzheimer’s disease (AD) is the most prevalent neurodegenerative disorder worldwide. It is characterized by a number of clinically important symptoms such as cognitive malfunctions and loss of memory (Teng 2024; Vejandla et al. 2024). The name AD was first introduced in 1906 by the German psychiatrist Alois Alzheimer. These days, about 70% of all human dementia cases have been related to AD (Yamazaki et al. 2019). AD is an age-related disorder, and in older individuals its incidence is roughly doubling every 5–10 years (Yamazaki et al. 2019). For persons between 65 and 69 years of age, a low incidence (0.6%) has been reported (Yamazaki et al. 2019). In contrast, for individuals older than 85 years, the incidence is dramatically (8.4%) higher (Yamazaki et al. 2019). The prevalence is also increasing with age rising from about 3% in the age group 65–74 years to almost 50% among individuals 85 years or older (Yamazaki et al. 2019). In 2020, AD affected about 50 million people in the world but this number is expected to grow because of the demographic changes. Globally, the number of adults of 65 years and older will reach about 973 million in 2030 (Yamazaki et al. 2019), and thus, much more AD cases will be diagnosed. In other estimates (2023), the global number of persons with AD dementia, prodromal AD, and preclinical AD was 32, 69, and 315 million, respectively. Together they constituted 416 million across the AD continuum or 22% of all persons aged 50 and above (Gustavsson et al. 2023). Taking into account that advanced age is the most significant risk factor for AD, one cannot underestimate the socio-economic impact of this disorder (Zhu and Sano 2006).

The most prominent morphological sign of AD is the extracellular deposition of beta-amyloid (Aβ) forming the characteristic amyloid plaques. In addition, intraneuronal accumulation of hyperphosphorylated tau proteins occurs, which leads to the formation of neurofibrillary tangles (NFT) disturbing the functionality of the neuronal cytoskeleton (Anand et al. 2014). Although NFT formation has also been reported in other neurodegenerative diseases, it is considered a pathological hallmark of AD.

Considering the time course of symptom development and the pathogenetic mechanisms, two major forms of AD can be distinguished. The early-onset form of AD (FAD), which represents less than 1% of all AD cases, is a genetic disease. It follows an autosomal dominant mode of inheritance and is usually fully developed before the age of 65 years (Sherrington, Rogaev et al. 1995; Hardy and Selkoe 2002). In most cases, FAD is caused by mutations in two different genes: (i) The first type of mutations is localized in the genes encoding for the proteins presenilin 1 (*Psen1*) and/or presenilin 2 (*Psen2*). The *Psen1* gene is located in a central region (q24.2–q24.3) of the long arm of chromosome 14. In contrast, the *Psen2* gene was mapped to the distal region (q42.13) of the long arm of chromosome 1. The corresponding proteins are essential for the catalytic activity of gamma-secretase, which plays an important role in the metabolism of the amyloid precursor protein (APP) (Rogaev et al. 1995; Xu 2009). (ii) The second type of mutations are localized in the *APP* gene. This gene is located in the central region (q21.3) of the long arm of chromosome 21. The pathophysiologically relevant mutations mainly occur in the region of the *APP* gene that encodes for the recognition sequences of the secretase proteases. These enzymes hydrolytically cleave APP forming A $\upbeta$ cleavage products. These proteolytic fragments are secreted into the extracellular space, aggregate, and form the amyloid plaques (Zhang et al. 2011a, b).

The major variant of AD is late-onset AD (LOAD), which usually affects patients with an age higher than 65 years (Haass and Selkoe 2007; Huang and Mucke 2012). Although a number of genetic and environmental risk factors have been described for LOAD, the expression of the *APOE4* allele is the major one. More than 15% of all LOAD patients carry this dysfunctional allele at the *APOE* gene locus. Homozygous allele carriers have a 20-fold higher risk for LOAD when compared with carriers of other *APOE* allele combinations (Corder et al. 1993; Association 2018). While the precise mechanism by which the APOE4 protein contributes to the pathogenesis of LOAD remains a subject of ongoing debate, recent experiments involving genetically modified mice suggested a strong impairment in the sortilin-dependent neuronal uptake of APOE lipids. This impairment may be caused by a compromised reshuffling of sortilin to the cellular membrane (14), which reduces fatty acid-binding protein-7-dependent intracellular lipid signaling (Asaro et al. 2021).

A cellular hallmark in both forms of AD is premature cell death, and investigations into the mechanisms of neuronal cell death have recently suggested that different types of cell death such as apoptosis (D’Arcy 2019), necrosis (Deroux et al. 2022), and ferroptosis (Tang et al. 2021) may be involved. Ferroptosis is a non-apoptotic form of regulated cell death that is characterized by dysregulated iron homeostasis and uncontrolled lipid peroxidation (Li et al. 2020). It has first been implicated in regression of tumor growth (Tang et al. 2020; He et al. 2021) but also in neuronal cell death in neurodegenerative diseases (Reichert et al. 2020; Bao et al. 2021).

Because of the complexity of AD (Teng 2024) and because of its socio-economic relevance (Zhu and Sano 2006), a large number of researchers have explored different aspects of this disease. Thus, writing a useful review on this disorder is rather challenging. In fact, a PubMed search (March 5, 2024) with the keywords “Alzheimer and review” revealed 52,367 hits. If a similar search was performed with the keywords “Alzheimer and review and APOE,” some 1820 hits were obtained. Finally, we repeated the search with the keywords “Alzheimer and review and APOE and ferroptosis.” Here, we found two papers (Plascencia-Villa and Perry 2021; Wang et al. 2024). We are well aware of the fact that such keyword-based database searches might sometimes be misleading. However, the low hit numbers we obtained in all of our database searches suggested to us that no review paper has recently been published, which addresses the roles of apolipoprotein E4 in oxidative lipid metabolism and ferroptosis in the pathogenesis of AD. These results encouraged us to write this review article. To avoid a major thematic overlap with previous review papers on related topics, we had a closer look at the two papers identified as positive hits in our database search strategy. In the first paper (Plascencia-Villa and Perry 2021), innovative preventive and therapeutic scenarios for AD are discussed. In the second study (Wang et al. 2024), the functions of sphingolipids in the pathogenesis of AD are reviewed. However, neither of these two references addressed the molecular relations between the APOE polymorphism, oxidative lipid metabolism, and ferroptosis in the pathogenesis of AD, and thus, there is no thematic overlap when one compares these two papers with our review.

## Apolipoprotein E and Alzheimer’s Disease

During the past 20 years, numerous large-scale epidemiological studies have indicated that the presence of an *APOE4* allele at the *APOE* gene locus is associated with an increased individual risk for the development of LOAD (Mamun et al. 2020; Sun et al. 2023). In fact, individuals carrying two *APOE4* alleles at this gene locus or an *APOE3* + *APOE4* allele combination have an increased risk for LOAD (Serrano-Pozo et al. 2021). At least one *APOE4* allele is present in 60% of LOAD patients (Xian et al. 2018), and the age for the onset of the disease is significantly reduced in *APOE4* allele carriers (Huang and Mucke 2012). On the other hand, individuals carrying *APOE2* or *APOE3* allele combinations are at lower risk (Zhang and Hong 2015). In Table 1, selected roles of APOE in the pathogenesis of AD and the corresponding pathomechanisms are summarized. Most of them will be discussed in more detail in the text.

**Table 1:** Roles of APOE in the pathogenesis of AD and the corresponding pathomechanisms

Effects of apoE4	Mechanism of action	Type of study
Aβ metabolism	APOE4 induces Aβ plaque formation (Koistinaho et al. 2004; Fernandez et al. 2019)	In vitro Clinical trials
	APOE4 reduces monomeric Aβ (Huynh et al. 2017), causes decrease in Aβ clearance (Safieh et al. 2019), and activates gamma-secretase (Lane-Donovan and Herz 2017)	In vivo
	APOE4 induces formation of APOE-fragment/Aβ heterodimers (Mouchard et al. 2019) review and Aβ complexes in brain vasculature (Martel et al. 1997; Kim et al. 2009)	In vivo
Tau phosphorylation	APOE4 enhances the phosphorylation of tau (Brecht et al. 2004)	In vivo
	APOE4 reduces monomeric Aβ (Huynh et al. 2017), causes decrease in Aβ clearance (Safieh et al. 2019), and activates gamma-secretase (Lane-Donovan and Herz 2017)	In vivo
	APOE4 proteolysis fragments induce hyperphosphorylation of tau proteins (Huang et al. 2001; Harris et al. 2003)	In vitro In vivo
	APOE4 interacts directly with tau in the cytoplasm to induce its hyperphosphorylation (Harris et al. 2004)	In vivo
Lipid metabolism	APOE4 increases the production of phospholipids and cholesteryl esters by MAM functions (Tambini et al. 2016)	In vitro
	APOE4 deficiency causes the lack of lipid transport in the brain (Carter et al. 2017)	In vivo
Neuroinflammation	APOE4 induces the expression of IL-1β, more than APOE3 (Guo et al. 2004; Dorey et al. 2014)	In vitro In vivo
	APOE4 pro-inflammatory role is stronger than APOE3 (Guo et al. 2004)	In vivo
	APOE4 causes the activation of the inflammatory receptor and the suppression of the anti-inflammatory receptor (Li et al. 2015)	In vitro
	APOE4 increase inflammation (Ophir et al. 2005; Cash et al. 2012; Dorey et al. 2014; Rodriguez et al. 2014; Du et al. 2015; Li et al. 2015) and neuroinflammation (Lukiw et al. 2008; Teter et al. 2016)	In vitro, in vivo Clinical trials In vitro
Vascular integrity/function	APOE4 increases the risk of vascular dementia and atherosclerosis (Mahley et al. 2009; Rohn 2014)	In vivo
	APOE4 carriers show cerebrovascular dysfunction (Bell et al. 2012; Tai et al. 2016)	In vivo Clinical trials
	APOE4 induces accumulation of Aβ, fibrin, and fibrinogen in the neuro-vasculature of AD patients (Cortes-Canteli et al. 2012; Rannikmäe et al. 2014)	In vivo Meta-analysis
Insulin and VEGF signaling	APOE4 disrupts insulin metabolism in neurons (Ong et al. 2014; Traversy et al. 2017)	In vivo Clinical trials
Synaptic plasticity	APOE4 inhibits neurite outgrowth in some cases (Nathan et al. 2002)	In vitro
	APOE3 activates LRP1, APOE receptor, heparin sulfate proteoglycan more than APOE4 (Hayashi et al. 2007)	Clinical trials
Mitochondrial function	APOE4 fragments reduce electron transport and the function of ATP synthase (Huang and Mucke 2012; Liu et al. 2013)	Clinical trials
	Expression of mitochondrial respiratory complexes I, IV, and V is downregulated in APOE4 carriers (Chang et al. 2005; Chen et al. 2011)	In vivo
	APOE4 leads to mitochondrial dysfunction and enhanced levels of ROS production (Liang et al. 2021)	In vivo and in vitro

### Apolipoprotein E Structures and Functions

Apolipoprotein E (APOE) is a small (34 kDa) protein that consists of 299 amino acids. As with other apolipoproteins (apolipoprotein A, apolipoprotein B48, apolipoprotein B100, apolipoprotein C, etc.), it plays a role in lipid transport in the blood plasma (Mahley et al. 1984). It exhibits antiatherogenic properties but clearly has additional biological functions (Ma et al. 2017). In mice, functional inactivation of the apolipoprotein E gene (*apoE* gene) induces athero-susceptibility and *apoE*^*−/−*^ mice develop significant lipid depositions in the arterial wall even when they are maintained on regular low-lipid chow diet (Song et al. 2012). In earlier studies, it has been reported that *apoE*^*−/−*^ mice have a higher risk for developing AD-related symptoms such as memory defects, tau protein hyperphosphorylation, leaky blood–brain barrier, and even Aβ deposits in the brain when compared with wild-type control animals (Lane-Donovan et al. 2016; Saul and Wirths 2017; Saroja et al. 2022). However, in more recent studies, the development of such symptoms could not be confirmed (Long and Holtzman 2019), and thus, the development of classical AD-related symptoms in *apoE*^−/−^ mice remains controversial.

In humans, the *APOE* gene is located in a central region (q13.32) of the long arm of chromosome 19. It involves 4 exons and 3 introns (Chawla et al. 2001). It is expressed at high levels in the liver, but also in peripheral organs, such as the lungs, kidneys, and adipocyte tissue and brain (Williams et al. 1985; Mahley 1988; Ang et al. 2008; Getz and Reardon 2009; Huang et al. 2009). In the central nervous system, the major source of the APOE protein is the astrocytes and the corresponding protein has been implicated in the cellular import of cholesterol and other lipids needed for basic cell functions. This lipid import is receptor-dependent and requires a functional low-density lipoprotein receptor (LDLR) (Liu et al. 2013). The human APOE protein consists of two distinct structural domains, which are interconnected by a flexible hinge region. The N-terminal domain (amino acid residues 1–167) folds into an antiparallel four-helix bundle. It carries the LDLR binding region between positions 136 and 150. The C-terminal domain (residues 206–299) consists of three alpha helices. It also involves a lipid-binding site that interacts with the helix bundle in the N-terminal domain (Momeni and Ferrari 2010; Martínez‐Oliván et al. 2014; Uddin et al. 2019).

**Fig. 1:**
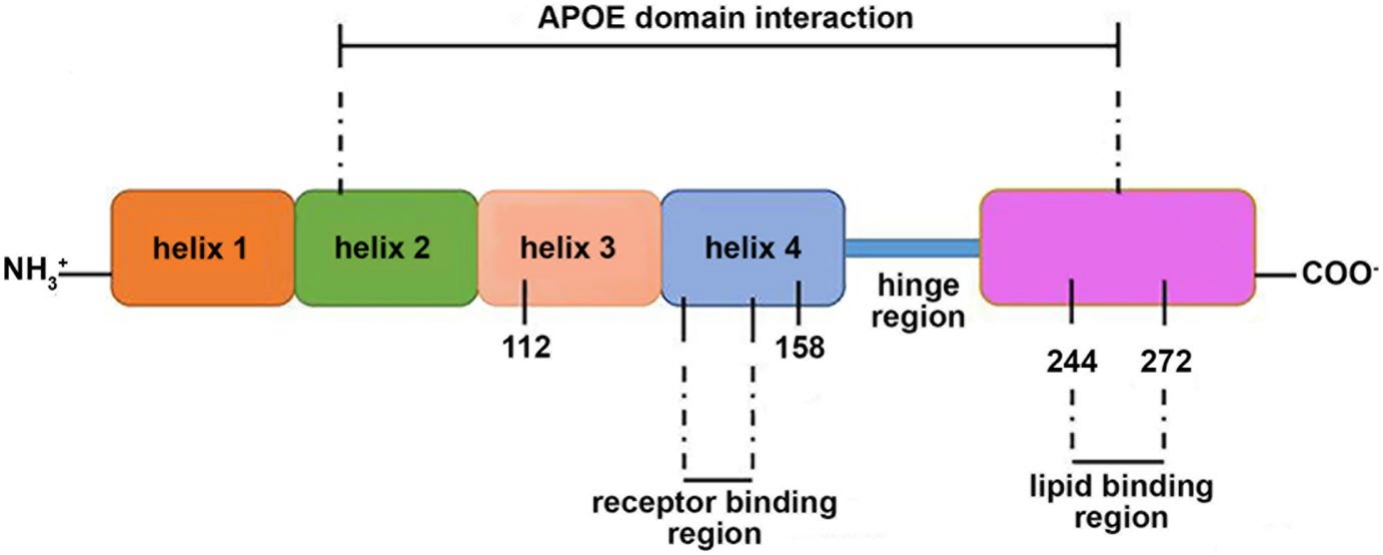
A schematic view of the apolipoprotein E structure. The APOE protein consists of an N-terminal and a C-terminal domain. The N-terminal domain involves four alpha helices and helix 4 carries the binding region for the LDL receptor. The C-terminal domain involves the lipid-binding subdomain localized between the amino acid residues 244–272. The lipid-binding subdomain has also been suggested as binding site for Aß peptides. The two domains are interconnected by a flexible hinge region, which ensures a high degree of structural flexibility for the protein. In the APOE2 protein, the amino acids 112 (helix 3) and 158 (helix 4) are occupied by two Cys residues. In APOE3, a Cys is localized at position 112 but a positively charged Arg is at position 158. The APOE4 protein carries two positively charged Arg residues in these positions. This image was modified from (Liu et al. 2013)

In humans, the apolipoprotein E gene (*APOE*) is present in three major polymorphic alleles, called *APOE2*, *APOE3*, and *APOE4*. These alleles encode three distinct protein isoforms (APOE2, APOE3, APOE4), which differ from each other by single amino acid exchanges. The *APOE2* allele encodes for a protein that carries Cys residues at positions 112 and 158 (Cys112, Cys158). In contrast, the *APOE3* allele encodes for a protein variant carrying a Cys only at position 112 but a positively charged Arg at position 158. The APOE4 protein involves two positively charged Arg residues at the respective positions (Weisgraber 1994; Liu et al. 2013). X-ray crystallographic analyses revealed that the presence of a Cys residue at position 112 induces hiding of the positively charged site chain of Arg61 between helices 2 and 3, which is the case for APOE2 and APOE3. On the other hand, a positively charged Arg at this position, which is present in the APOE4 protein, forms a salt bridge with Glu109 and this salt bridge causes exposure of the positively charged Arg61 on the protein surface. In that case (Fig. 1), Arg61 of the N-terminal domain (helix 2) interacts with the negatively charged site chain of Glu225 of the C-terminal domain and this salt bridge tightens the two APOE4 domains together (Phillips 2014). The postulated interdomain tightening reduces the water solubility of the protein and increases its aggregation behavior.

In the middle European population, 60% of the inhabitants carry two *APOE3* alleles at the *APOE* gene locus. In contrast, in 20–25% of the inhabitants, one *APOE3* allele and one *APOE4* allele are simultaneously present (heterozygosity) (Singh et al. 2006). Other *APOE* allele combinations, such as *APOE2* + *APOE3* (13%), *APOE4* + *APOE4* (1.7%), *APOE2* + *APOE4* (1.3%), or *APOE2* + *APOE2* (0.5%), occur less frequently. It should, however, be stressed that the occurrence frequency of the *APOE4* allele is higher in northern Europe but lower in the south (Noya and Capurso 1999). In Africa, the *APOE4* allele occurs more frequently, but in Asia, the *APOE4* allele is distributed with a lower frequency (Wang et al. 2021).

The 3D structure of the recombinant human APOE3 protein (PDB 2L7B) has been solved by NMR studies (Chen et al. 2011). In addition, there are a number of crystal structures for different APOE truncation constructs in the PDB database. The C-terminal domain of the human APOE3 protein presents a large exposed hydrophobic surface that likely initiates interactions with lipids, but can also bind Aß peptides. According to the interpretation of the X-ray coordinates by the authors, the unique topology of the APOE protein precisely regulates its tertiary structure to permit only one possible conformational adaptation upon lipid and/or Aß binding (Chen et al. 2011). It also provides a double security in preventing lipid-free and partially lipidated APOE from premature binding to APOE receptors during receptor biogenesis. This topology further ensures the optimal receptor-binding activity by the fully lipidated protein during lipoprotein transport in circulation and in the brain. Since lipids and Aß peptides compete for the same binding region, the degree of APOE lipidation modifies the affinity of Aß binding (Wisniewski and Drummond 2020). Since APOE4-containing lipoproteins are less lipidated, APOE4-Aβ complexes are less stable, resulting in reduced intracellular APOE4-Aβ levels and increased Aß accumulation (Tai et al. 2014). The binding affinity of both APOE3 and APOE4 for Aß peptides is similar (*K*_D_ of about 20 nM), but it greatly depends on the conformational state of the Aβ peptide used for the binding studies. Preferential binding was observed when the Aß peptides adopt a β-sheet conformation, but there was hardly any difference between APOE3 and APOE4 (Golabek et al. 1996). Consistent with these results, it has also been shown that both APOE3 and APOE4 interact with Aß peptides to form novel fibrillar structures. Interestingly, APOE4 forms these complex fibrils more avidly (Sanan et al. 1994).

### Cerebral Expression of APOE and Possible Biological Functions

As the genes encoding for the other apolipoproteins, the different *APOE* alleles are mainly expressed in the liver. To explore the expression of the mouse apoE lipoprotein in more detail, reporter mice were constructed, in which the enhanced green fluorescent protein (EGFP) was inserted into the *apoE* gene locus (EGFP-apoE mice). In these animals, the EGFP was highly expressed in hepatocytes, in peritoneal macrophages but also in a subset brain astrocytes (Xu et al. 2006). Although normal hippocampal neurons do not express EGFP, kainic acid treatment induced EGFP expression suggesting apoE is expressed in neurons in response to excitotoxic injury. Smooth muscle cells of large blood vessels and cells surrounding small vessels in the CNS did also express the reporter gene (Xu et al. 2006). Unfortunately, whether these findings can be translated into the human situation has not been explored in detail.

To address this point, in situ hybridization was carried out on paraffin-embedded and frozen brain sections from three nondemented controls and five AD patients. Using specifically designed antisense *APOE* probes specific in situ hybridization signals were detected glial cells but also in selected neurons in the cerebral cortex and in the hippocampus. In the hippocampus, a high density of APOE mRNA-positive neurons was detected in sectors CA1 to CA4 and the granule cell layer of the dentate gyrus (Xu et al. 1999). In the cerebellar cortex, APOE mRNA was seen only in Bergmann glial cells and scattered astrocytes but not in Purkinje cells or granule cell neurons. Taken together, these data demonstrated that the APOE mRNA is present at high amounts in human glial cells but also in certain types of neurons in the frontal cortex and the hippocampus of humans (Xu et al. 1999). When the APOE4 protein is expressed in neurons, it undergoes proteolysis, which results in the generation of neurotoxic fragments. These fragments cause mitochondrial dysfunction and rearrangements in the cytoskeleton. Interestingly, the APOE4 protein exhibits stronger neurotoxic effects than the APOE3 and APOE2 proteins and blocking the interactions between the APOE domains reversed the detrimental activities (Mahley and Huang 2012).

In macrophages, expression of the polymorphic APOE proteins is highly regulated on transcriptional and post-transcriptional levels (Larkin et al. 2000) and similar mechanisms might apply for neurons. Studying expression of the different APOE isoproteins in neuronal cells, a splicing variant of the APOE mRNA was detected that still carries intron-3 of the APOE gene. This splicing variant (APOE-I3) was detected in various neuronal cell lines and in primary neurons of mice and humans. Cell fractionation studies indicated that more than 98% of the APOE-I3 mRNA copies were not exported into the cytosol, and thus, they are not translated into functional proteins. In transfected primary neurons, APOE expression did increase dramatically when intron-3 was deleted from the transfection construct. These data and the results of additional challenging experiments suggested that neuronal expression of the *APOE* gene under normal conditions is prevented by the minimal nuclear export of the APOE-I3 mRNA. However, in response to cell injury, the APOE-I3 mRNA is converted to the mature APOE mRNA, which is rapidly exported into the cytosol and is then translated to the corresponding protein (Xu et al. 2008).

As other apolipoproteins, APOE regulates the cholesterol metabolism in the central nervous system and expression of the APOE4 variant modifies neuronal cholesterol homeostasis. In fact, APOE4 expression elevated endogenous cholesterol synthesis by upregulating the genes encoding for the cholesterol-biosynthesizing enzymes (Piccarducci et al. 2023). In contrast, the cellular concentrations of acetyl-CoA, which constitutes the major substrate of endogenous cholesterol biosynthesis, were reduced and this was suggested to impact acetylcholine biosynthesis (Piccarducci et al. 2023). However, it still remains a matter of discussion how exactly the dysregulated cholesterol homeostasis does impact cholinergic communication, neurotoxicity, and neuronal death.

### The Role of APOE4 in Aggregation of Amyloid-Beta Peptides

**Fig. 2:**
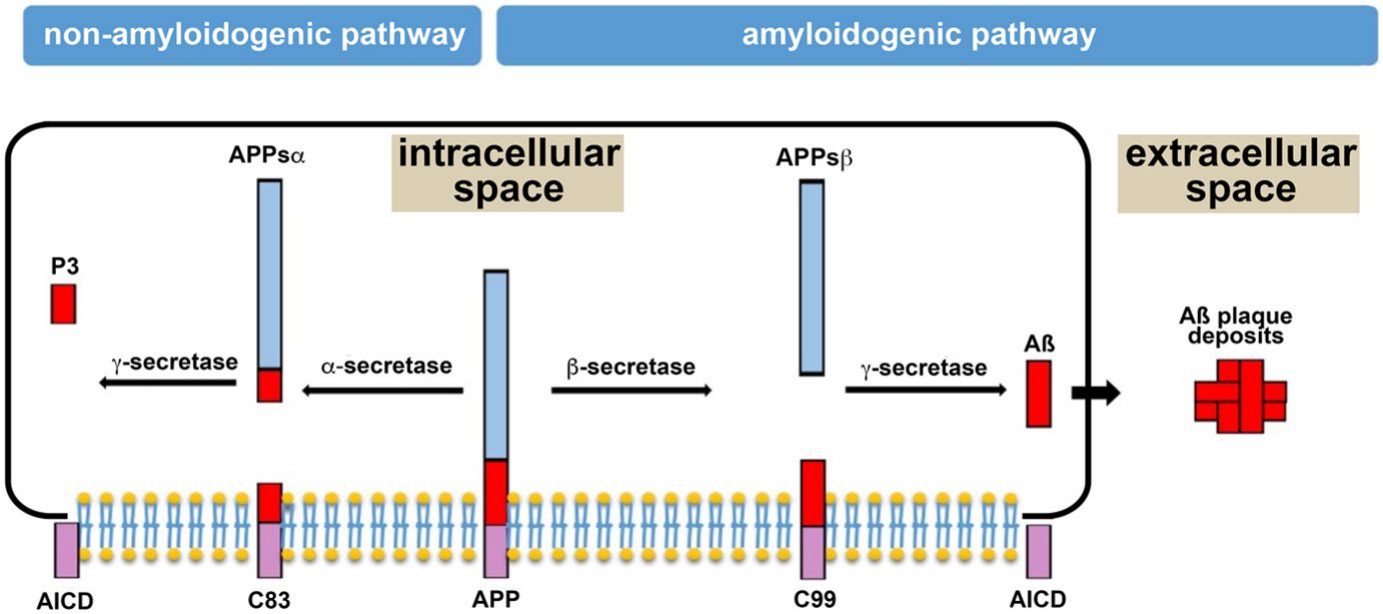
Proteolytic cleavage of the amyloid precursor protein (APP) via the non-amyloidogenic and the amyloidogenic pathways. Left side: non-amyloidogenic pathway. This pathway involves sequential proteolysis of the amyloid precursor protein (APP) by alpha-secretase and gamma-secretase, which results in the formation of the non-toxic and water-soluble fragments APPsα and P3. Since the cleavage site of gamma-secretase is located in the membrane-spanning domain of APP (AICD), a truncated version of this domain is left in the plasma membrane. This pathway is considered a protective mechanism against amyloid plaque formation. Right side: amyloidogenic pathway. In this pathway, the initial proteolysis of APP is catalyzed by beta-secretase yielding the water-soluble APPsß cleavage fragment. Next, gamma-secretase cleaves the C99 fragment, which is still anchored in the plasma membrane, within its membrane-spanning domain leaving a truncated version of AICD in the plasma membrane. Since gamma-secretase cleavage is not strictly regiospecific, several Aß peptides can be formed. Unfortunately, all Aβ peptides have the potential to aggregate, which leads to the formation of Aß plaques. This image was modified from (Sasmita 2019)

There are two pathological hallmarks in the pathogenesis of AD: (i) the formation of extracellular amyloid plaques in the brain and (ii) the formation of intracellular neurofibrillary tangles in neurons (Hardy and Selkoe 2002). Amyloid plaque formation commences with secretase-catalyzed (Zhang et al. 2011a, b) cleavage of the amyloid precursor protein (APP) that is abundantly expressed in the CNS (Aydin et al. 2012). It is present in the plasma membrane of neurons as transmembrane protein, but it was also detected in polarized epithelial cells and in circulating blood cells. It is metabolized proteolytically via two alternative routes (Fig. 2).

When metabolized via the non-amyloidogenic pathway, APP is first cleaved by alpha-secretase. Alternatively, in the amyloidogenic pathway, APP undergoes initial cleavage by beta-secretase. The C-terminal peptide (C99) of the beta-secretase reaction is further cleaved by gamma-secretase and so the full-length β-amyloid peptide (Aβ) is formed. Gamma-secretase cleavage takes place in the membrane-spanning domain of the APP protein. Since gamma-secretases do not exhibit absolute regiospecificity, several Aß peptides are formed, but Aß_40_ and Aß_42_ are dominant (Zheng and Koo 2006). These peptides are released as monomers into the extracellular space, but here they progressively aggregate. Interestingly, compared with Aß40, Aß42 is more prone to aggregation and exhibits a higher degree of neurotoxicity (El-Agnaf et al. 2000). Under physiological conditions, APP is preferentially metabolized via the non-amyloidogenic pathway and there is a stable equilibrium between the production of Aβ peptides and their clearance from the extracellular space (Evin and Weidemann 2002; Bandyopadhyay et al. 2007).

The *APOE* polymorphism impacts the efficiency of Aß plaque formation. In fact, APOE4 protein expression augments Aβ production by activating gamma-secretase (Lane-Donovan and Herz 2017) and by forming mixed peptide oligomers. The full-length APOE protein has a molecular weight of about 34 kDa, but it is cleaved in the brain yielding an APOE-18 kDa cleavage peptide (Mouchard et al. 2019). This peptide is neurotoxic by itself (Tolar et al. 1999), but it also forms even more toxic mixed peptide oligomers with Aß fragments (Manelli et al. 2007). Such mixed peptide oligomers are formed in much lower quantities when corresponding cleavage peptides of the APOE2 and APOE3 proteins are present (Mouchard et al. 2019). Employing transgenic mice expressing different human *APOE* alleles, it has been shown that APOE4-expressing animals show more efficient amyloidogenesis than mice expressing the APOE3 and APOE2 isoforms (Huynh et al. 2017). Moreover, experiments with *APOE-*inducible mouse models basically confirmed this observation (Liu et al. 2017a). Mixed APOE4-Aβ peptide complexes also occur in the cerebral vasculature. Here again, APOE4-Aß complexes were more abundantly present than APOE2-Aβ or APOE3-Aβ complexes (Martel et al. 1997; Kim et al. 2009).

### The Role of APOE4 Isoproteins for the Clearance of Aß Aggregates

The steady-state tissue concentrations of Aß in the brain are regulated not only by the extent of A $\beta$ formation (influx control) but also by its proteolytic clearance (efflux control). Although the efflux mechanisms have not been studied in detail, several proteins including APOE isoforms have been implicated in the clearing process (Castellano et al. 2011). Under pathological conditions, there is a shift of the alpha-secretase pathway (non-amyloidogenic) to the beta-gamma-secretase (amyloidogenic) pathway, which leads to the accumulation of Aβ within the extracellular space in the brain. In AD patients, the total levels of Aβ_42_ monomers in the cerebrospinal fluid decrease, but concentrations of oligomeric Aβ levels increase (Tai et al. 2013). Various studies indicated that the APOE4 protein speeds up Aβ aggregation but simultaneously attenuates Aβ clearance (Safieh et al. 2019).

The lipidation of the APOE protein in the central nervous system is an important process. Astrocytes synthesize the different APOE protein variants and release them into the extracellular space. Cholesterol and other lipids are also formed by these cells and are released via the ATP-binding cassette A1 (ABCA1) transporter. Extracellularly, lipid-laden APOE nanoparticles are formed (Hauser et al. 2011). These nanoparticles bind soluble A $\beta$ peptides and form insoluble APOE-A $\beta$ complexes. These lipidated complexes can be cleared from the extracellular space via receptor-mediated endocytosis by neurons and/or microglia cells, and three different cell surface receptors have been implicated in the clearing process: (i) low-density lipoprotein receptor (LDLR), (ii) low-density lipoprotein receptor-related protein-1 (LRP1), and (iii) heparin sulfate proteoglycan (HSPG). The efficiency of the clearing process depends on the composition of the APOE-Aß complexes, and APOE2-containing complexes are most effectively cleared (APOE2 > APOE3 >  > APOE4). In contrast, APOE4-containing complexes are rather resistant to clearance and will accumulate. A $\beta$ oligomers and mixed APOE-Aß complexes can cross the blood–brain barrier (BBB) via LRP1-dependent pathways to enter the blood stream. There, they are degraded by circulating proteases, such as insulin-degrading enzyme (IDE) or angiotensin-converting enzyme (ACE), but also by more unspecific proteases, such as neprilysin (Nep) (Kim et al. 2009; Dries et al. 2012).

Neprilysin, which has been implicated in cerebral clearance of A $\beta$ peptides (Pacheco-Quinto et al. 2013; Hüttenrauch et al. 2015), is a zinc-containing unspecific metalloprotease that is expressed in many cells and tissues (kidney, lungs, adipose tissue, bone immune cells, testis, and others). In the brain, neprilysin expression occurs less abundantly when compared with the kidney and lungs, but it has been detected in GABA-ergic and metabotropic glutamate 2/3 receptor-positive neurons (Fukami et al. 2002). In contrast, cholinergic neurons are apparently free of neprilysin (Fukami et al. 2002). The neprilysin protein is encoded by the *MME* (membrane metalloendopeptidase) gene, which is located in the central region of the long arm of chromosome 3. The enzyme cleaves a number of different biologically relevant peptides including bradykinin, angiotensin II, substance, and enkephalins. It also hydrolyzes A $\beta$ peptides. *MME*^−/−^ mice develop AD-like symptoms and subtle Aß deposits in the brain. Thus, neprilysin is an important ß-amyloid-degrading enzyme (Hafez et al. 2011).

**Fig. 3:**
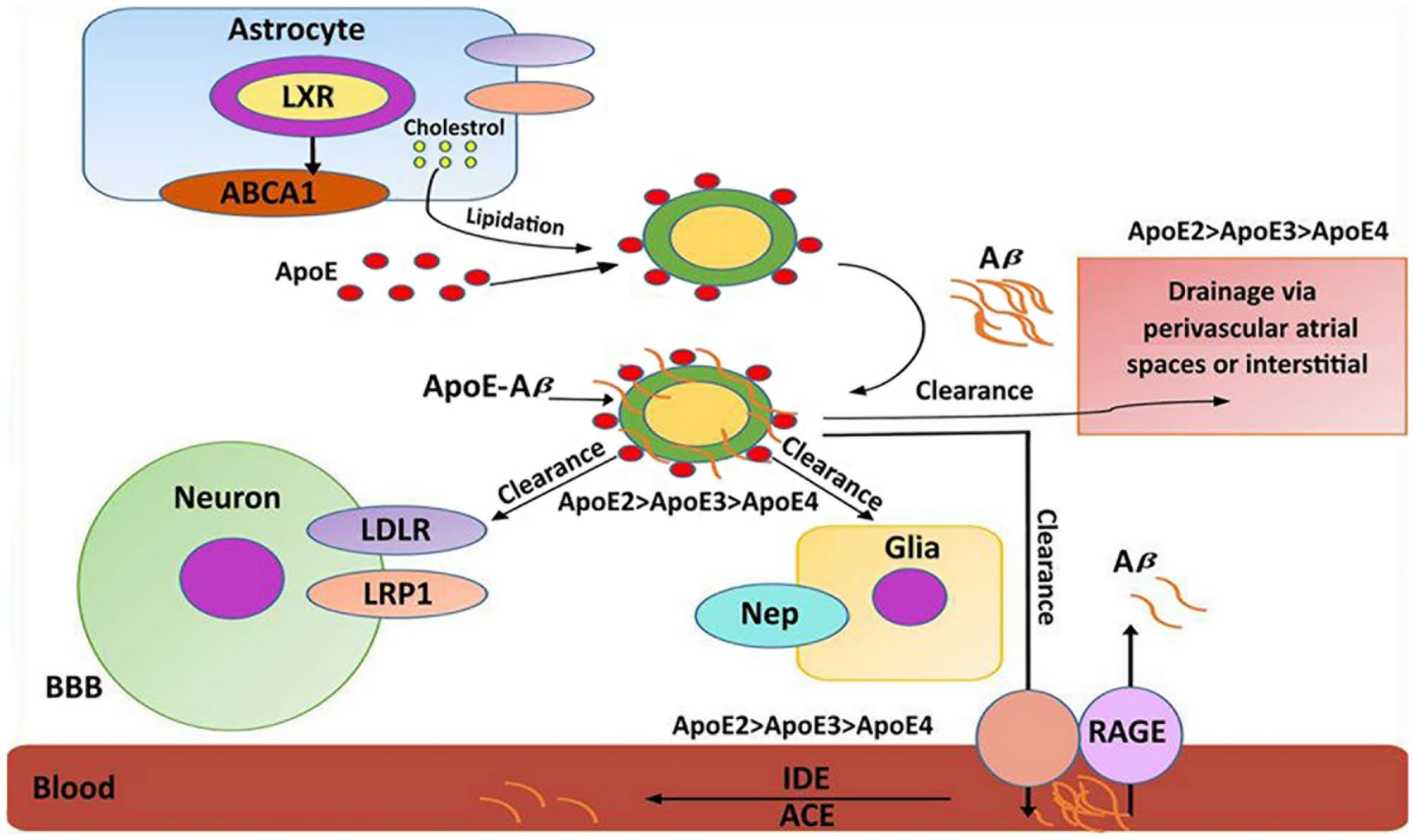
Clearance of Aβ peptides from the brain. There are three major pathways by which Aβ is cleared from the extracellular compartment of the brain: **i** extracellular proteolytic degradation, **ii** cellular uptake by neurons and glia cells and subsequent intracellular lysosomal degradation, and **iii** transfer from the extracellular space of the brain into the blood and proteolytic cleavage by circulating blood proteases. Abbreviations: LDLR, low-density lipoprotein receptor; LRP1, low-density lipoprotein receptor-related protein; HSPG, heparin sulfate proteoglycan; BBB, blood–brain barrier; ABCA1, ATP-binding cassette A1 transporter; LXRs, liver X receptors; IDE, insulin-degrading enzyme; ACE, angiotensin-converting enzyme; Nep, neprilysin; RAGE, receptor for advanced glycosylation end products. This image was modified from (Yoon and Jo 2012)

RAGE, which is also called AGER (advanced glycation end product receptor), is a cell surface receptor that interacts with A $\beta$ peptides and has been implicated in the clearance of A $\beta$ complexes from the brain. Although RAGE facilitates the transport of A $\beta$ across the BBB (beneficial process), it may also import Aß peptides from the circulation into the brain and thus may induce neuroinflammation (Montagne et al. 2020). Since A $\beta$ import is clearly detrimental for the brain, RAGE may play a dual role in the pathogenesis of AD (Yan et al. 2012). The processes involved in the clearing of Aß peptides are summarized in Fig. 3.

### APOE4 and Tau Protein Pathology

The second major morphological hallmark of AD is the neurofibrillary tangles (Ando et al. 2014). These tangles (NFTs) constitute fibrous protein structures formed within the cellular body of neurons but also in the dendritic spines. They consist of hyperphosphorylated isoforms of the tau protein, which aggregate intracellularly and thus impair neuronal function (Ando et al. 2014; Reitz and Mayeux 2014). In epidemiological studies, a statistical correlation was found between the presence of the *APOE4* allele and the concentration of tau proteins in the cerebrospinal fluid. Interestingly, this correlation showed a higher degree of statistical significance in females than in males (Hohman et al. 2018). Moreover, NFT formation appears to correlate more closely with the cognitive decline of AD patients than with the formation of amyloid plaques. Thus, NFT formation may be considered a more reliable diagnostic parameter to judge the clinical severity of AD than amyloid plaque formation (Giannakopoulos et al. 2003).

The tau proteins (tubulin-associated units) represent a family of water-soluble protein isoforms, which are encoded by the *MAPT* (microtubule-associated protein tau) gene. In humans, this gene is located in a central region (q21.31) of the long arm of chromosome 17 and is expressed in a large number of mammalian cells including neurons (Neve et al. 1986; Goedert et al. 1988). In other cells of the CNS (astrocytes, oligodendrocytes), the *MAPT* gene is only expressed at low levels. Tau proteins have been implicated as regulatory proteins in the formation of microtubules and play important roles in the structure and function of the cytoskeleton. In neurodegenerative diseases including AD (Morris et al. 2011), the water-soluble tau proteins are frequently hyperphosphorylated and form insoluble aggregates (neurofibrillary tangles). These tangles are neurotoxic and impair neuronal functions (Hampel et al. 2010).

Under physiological conditions, tau proteins usually do not occur as free cytosolic proteins but they associate with various binding partners such as microtubules, Src, and APOE. However, when phosphorylated, the tau proteins are released from their binding partners and lose their specific functions (Bhaskar et al. 2005; Morris et al. 2011). In transgenic mice, overexpressing APOE4 in neurons tau hyperphosphorylation was observed. These observations suggested that APOE4 may play a key role in the progression of neuronal defects related to AD (Brecht et al. 2004). Furthermore, neuronal proteolysis of APOE4 leads to the generation of truncated APOE fragments which trigger tau phosphorylation (Huang et al. 2001; Harris et al. 2003). In APOE4 transgenic mice, tau hyperphosphorylation was associated with activation of the extracellular signal-regulated kinase (ERK) and zinc ions apparently play a role in this process (Harris et al. 2004).

In other mouse models, the potential role of apoE isoproteins in tau phosphorylation is controversial. When the degree of tau phosphorylation was quantified in *apoE*^*−/−*^ mice, hyperphosphorylation was observed and this data suggested that expression of the *apoE* gene might downregulate tau phosphorylation (Genis et al. 1995). However, in a follow-up study, this observation could not be confirmed since the patterns of tau phosphorylation were similar when wild-type mice were compared with *apoE*^*−/−*^ animals. Here, the authors concluded that the lack of expression of the apoE proteins may not interfere with expression, distribution, and phosphorylation of tau proteins (Mercken and Brion 1995). The possible reasons for these controversial data remain unclear but it might be related to background problems of the employed *apoE*^*−/−*^ mice, to specificity problems of the employed antibodies, or to other technical reasons. When Aß oligomers were injected into the lateral ventricles of *apoE*^*−/−*^ mice and of corresponding wild-type controls, it was found that the degree of tau phosphorylation was higher in wild-type mice. Moreover, in vitro studies indicated APOE4-treated *apoE*^*−/−*^ neurons exhibited more phosphorylated tau proteins than APOE3- and APOE2-treated neurons. Taken together, these results suggest that APOE may facilitate tau phosphorylation in an isoform-specific way (Hou et al. 2020). However, the molecular basis for this observation has not been explored in this study.

To address this point, it was explored whether APOE treatment of neurons affected the catalytic activity of enzymes that have previously been implicated in tau phosphorylation, such as glycogen synthase kinase 3beta (GSK3ß), P35, and cyclin-dependent protein kinase 5 (CDK5). Treatment of primary neurons with APOE (2 µM) attenuated the cellular concentrations of phospho-GSK3ß, P35, and CDK5 and also decreased the levels of phosphorylated tau. The alteration of tau phosphorylation was blocked by an inhibitor of the low-density lipoprotein receptor family suggesting that the observed effects were due to a specific receptor-ligand interaction. From these data, the authors conclude that APOE isoproteins might modulate tau phosphorylation via several receptor-dependent phosphorylation pathways (Hoe et al. 2006). When the experiments were repeated at 100 nM APOE concentrations, tau phosphorylation was not affected. Since the APOE concentrations used in these experiments were rather high (2 µM), it remains to be shown whether these in vitro data are of any in vivo relevance.

### APOE4 and Gene Expression Regulation

All nucleated human cells involve the same nuclear genome, but only a subset of these genes is expressed at a given time point in a given cell type. The decision, which gene is expressed at a certain time point, is made by a complex regular network that is generally referred to as gene expression regulation (Pope and Medzhitov 2018). In these regulatory networks, expression of a certain gene product modifies the expression of other genes. In other words, expression of the APOE4 protein instead of the other APOE isoforms alters the expression of other genes. Although the heritability of AD is high, knowledge of the disease-associated genes and their expression regulation still remains limited. However, recent studies have revealed that changes in gene expression patterns of AD-related genes strongly impact neuronal function and neurodegeneration. In fact, activation and inactivation of transcription (Jiang et al. 2013; Yang et al. 2023) and translation factors (Oliveira and Klann 2022), expression of non-coding RNA species (Zhou et al. 2019), alternative splicing (Biamonti et al. 2021; Farhadieh and Ghaedi 2023), and epigenetic mechanisms (Sharma et al. 2020) may play a role in the pathogenesis of AD (Bagyinszky et al. 2020).

Transgenic mice expressing human APOE3 or APOE4 isoforms show markedly distinct gene expression patterns in different brain regions (Lattanzio et al. 2014). For instance, the steady-state mRNA concentrations encoding for the peptidyl-prolyl cis/trans isomerase (Pin1) were significantly higher in the hippocampus of APOE4-expressing mice than in the corresponding regions of APOE3-expressing control animals. In contrast, in the cortex of the entorhinal and parietal regions, lower expression levels were detected. Sirt1 levels were significantly reduced in the frontal cortex of APOE4 mice, and these alterations may play a role in APOE4-associated memory impairments. Moreover, in APOE4 mice, presenilin (PS) mRNA levels were reduced in the frontal cortex, which might affect APP processing. In contrast, cellular levels of the brain-derived neurotrophic factor (BDNF) did not differ between APOE3 and APOE4 mice in any analyzed brain region (Lattanzio et al. 2014). Taken together, these data show that transgenic expression of the APOE4 protein dysregulated the expression characteristics of the *Pin1*, *Sirt1*, and *PS1* genes in different brain areas and the observed differences might contribute to the increased AD vulnerability of APOE4 mice. However, it remains to be explored in the future whether or not similar expression regulation characteristics also occur in the brains of human AD patients.

Impairment of adult neurogenesis is part of AD in humans, and this pathophysiological aspect involves altered neuronal gene expression patterns. *Post-mortem* transcriptome analyses of human AD brains indicated an upregulation in the expression of neural progenitor and proliferation markers together with an antiparallel downregulation in the expression of later neurogenic markers (Gatt et al. 2019). Thus, the overall level of human adult neurogenesis is reduced during the later stages of AD which may be related to compromised maturation and integration of “new-born” neurons. However, the exact molecular mechanisms underlying these differences between normal and compromised neurogenesis remain to be explored in the future.

### APOE4 and Lipid Metabolism

#### APOE4 in Cholesterol, PUFAs, and Lipid-Driven Energy Metabolism

APOE is an antiatherogenic apolipoprotein (Greenow et al. 2005). In contrast to wild-type mice, *apoE*-deficient mice (*apoE*^−/−^ mice) are athero-susceptible and these animals are frequently used to explore the mechanistic basis of human atherogenesis (Ma et al. 2017). APOE is expressed in various tissues including liver, lung, brain, spleen, ovary, kidney, and adrenal gland (Mahley 1988), and its canonical function is to contribute to HDL-mediated reverse cholesterol transport. Via this mechanism, it is responsible for the removal of excessive cholesterol from the plasma membranes of peripheral cells. After cholesterol loading, APOE is transported to the liver, where the cholesterol is converted to bile acids and excreted with the feces (Mahley 1988). However, more than 80% of the bile acids secreted into the gut are subsequently re-absorbed into the blood (entero-hepatic circulation) (Roberts et al. 2002). In other words, enteral excretion of cholesterol is limited.

Alimentary intake of omega-3 polyunsaturated fatty acids, such as eicosapentaenoic acid (EPA) and docosahexaenoic acid (DHA), has been associated with a reduced risk for the development of LOAD (Troesch et al. 2020; Power et al. 2022). However, this link may not be functional in *APOE4* allele carriers. This conclusion was based on the finding that *APOE4* allele carriers beta-oxidize more omega-3 polyenoic fatty acids than *APOE2* or *APOE3* carriers. Consequently, *APOE4* allele carriers have fewer omega-3 polyenoic fatty acids available for introduction into the membrane lipids, which may reduce the fluidity of the neuron plasma membrane and for the formation of eicosanoids (see “Eicosanoids and APOE4 in the Pathogenesis of AD”) and/or endocannabinoids (see “Endocannabinoids and APOE4 in the Pathogenesis of AD”).

Mitochondria-associated membranes (MAM) represent a region of the endoplasmic reticulum (ER) that is reversibly connected with mitochondria. These membranes have been implicated in the import of lipids synthesized in the ER into the mitochondria, and thus, they are essential for regular mitochondrial function (Vance 2014). When astrocytes were treated with astrocyte-conditioned medium containing APOE4, communication of the ER with mitochondria was significantly improved when compared with APOE3-containing astrocyte-conditioned medium (Tambini et al. 2016). These data are consistent with the assumption that the pathogenesis of AD involves an upregulation of MAM functionality. Although the molecular basis for these effects has not been explored in detail, a possible mechanism includes dysregulation of protein phosphatase 2A. This enzyme has previously been reported to modulate MAM formation in hepatocytes, and recently, this data has been confirmed for SH-SY5Y human neuroblastoma cells. When protein phosphatase 2A was inhibited in these cells, MAM formation was modified and this alteration correlated with an elevated mitochondrial Ca^2+^ influx and with disrupted mitochondrial membrane potential (Chaiwijit et al. 2023). The authors concluded that protein phosphatase 2A may play an important role in the regulation of MAM formation in neuronal cells and thus in the pathogenesis of AD.

In a detailed lipidome study, the lipid composition of MAM and other subcellular fractions were explored using a cellular in vitro model of AD. For this purpose, the authors compared the neuroblastoma cell line N2A that overexpressed the familial Swedish APP mutant and corresponding control cells. In this in vitro AD model, a downregulation in the relative abundance of several phosphatidylcholine and phosphatidylethanolamine species was observed but the biological relevance of these findings remains unclear (Fernandes et al. 2024). MAM formation is a key process in autophagy, and dysregulated autophagy constitutes a hallmark in different types of neurodegenerative diseases such as AD and Parkinson’s disease (Kulkarni et al. 2024). Although the molecular bases of autophagy have been explored in detail (Glick et al. 2010; Mizushima and Komatsu 2011), it still remains unclear which of the different mechanisms are most important for AD and how exactly these mechanisms are triggered.

Dysregulated lipid metabolism of glia cells leads to the formation of lipid droplets, and these transient lipid storage organelles are considered early biomarkers of neurodegeneration (Liu et al. 2015). ApoE deficiency in mice induces hyperlipidemia, which leads to lipid deposition in the wall of the arteries (Jofre‐Monseny et al. 2008; Maeda 2011). Whether apoE deficiency also impacts the lipid metabolism of the brain is currently a major topic of discussion, but more detailed studies are required to understand the exact mechanism of APOE4 function in neuronal lipid metabolism (Liu et al. 2017b).

#### Eicosanoids and APOE4 in the Pathogenesis of AD

Eicosanoids and related compounds are lipid mediators, which are synthesized from arachidonic acid and other omega-3 and omega-6 PUFAs (Simopoulos 2002). As pleiotropic signaling molecules (Dimitrow 2009), they have been implicated in the regulation of neuronal function and thus may play a role in the pathogenesis of AD. Eicosanoids are biosynthesized via three different metabolic pathways (Biringer 2019):

(i) Prostaglandin G synthase (PTGS) pathway: the major end products of this metabolic pathway, which is more commonly known as the cyclooxygenase (COX) pathway, are prostaglandins, thromboxanes, and prostacyclins. These oxidized PUFA derivatives represent short-living endocrine, paracrine, or autocrine metabolites, which mainly exhibit their biological functions via binding to G-protein-coupled cell surface receptors. The key enzyme of this metabolic route is PTGS (COX), which is expressed in two different isoforms. PTGS-1 (COX-1) is constitutively expressed in many different cell types and is responsible for the formation of prostaglandins, regulating a large number of physiological processes such as gastroprotection (Langenbach et al. 1995; Kulmacz 1998), urine production (Dubois et al. 1998), thrombocyte aggregation (Yu et al. 2005), and pain perception (Fiorucci et al. 2001). In contrast, PTGS-2 (COX-2) is mainly expressed in inflammatory cells and plays an important role in the biosynthesis of pro-inflammatory mediators (Funk 2001). Selective PTGS-2 inhibitors (COXIBS) are frequently prescribed as anti-inflammatory drugs (Benelli et al. 2018). Although some COXIBS have adverse cardiovascular side effects (Cairns 2007), they are frequently prescribed as anti-inflammatory drugs. The two PTGS isoforms are encoded by two different homologous genes, which are located in a distal region of the long arm of chromosome 9 (PTGS-1) and in a central region of the long arm of chromosome 1 (PTGS-2).
(ii) Arachidonic acid lipoxygenase (ALOX) pathway: the human genome involves six functional *ALOX* genes (*ALOX5*, *ALOX15*, *ALOX15B*, *ALOX12*, *ALOX12B*, and *ALOXE3*). Except for the *ALOX5* gene, which is located in the centromere region of chromosome 10, all other *ALOX* genes have been mapped to a joint *ALOX* gene cluster on the short arm of chromosome 17. The encoded enzymes catalyze the dioxygenation of free and esterified PUFAs to the corresponding hydroperoxy compounds. In vivo, these lipid hydroperoxides are rapidly reduced by peroxidases to the corresponding alcohols, and thus, hydroxylated PUFAs are the first stable reaction product. When these hydroxy PUFAs are present in large quantities in the lipid bilayer of biomembranes, the membrane structure is disturbed and the cells or subcellular organelles may fall apart (Biringer 2019). Although the six different human ALOX isoforms share a high degree of structural similarity, their functional characteristics are very different and single knockout studies of mouse *Alox* genes suggested different biological functions for the different isoenzymes (Funk et al. 1996; Epp et al. 2007; Hallenborg et al. 2010a, b). In other words, the coding multiplicity of the ALOX isoforms is not an indication of functional redundancy. ALOX isoforms are also involved in the biosynthesis of anti-inflammatory and pro-resolving mediators such as lipoxins (Spite et al. 2009; Schebb et al. 2022), resolvins (Tjonahen et al. 2006), and maresins (Schäfer et al. 2023). Although the biological role of these ALOX metabolites has recently been challenged (Schebb et al. 2022; O’Donnell et al. 2023), they might play a regulatory role in neuroinflammation (Yao et al. 2005; Yang et al. 2010) and thus in the pathogenesis of AD. Among the different ALOX isoforms, ALOX5 and ALOX15 have been suggested to be of particular relevance (Czapski et al. 2016). The intracellular catalytic activity of ALOX isoforms depends on the cellular redox state and expression of the ALOX5 and ALOX15 genes is inversely regulated by cytokines (Spanbroek et al. 2001).
(iii) Cytochrome P450 pathway: cytochrome P450 isoforms are oxygen-activating enzymes and most of them function as monooxygenases (Hrycay and Bandiera 2012). For eicosanoid biosynthesis, they mainly catalyze epoxygenation of unsaturated fatty acids (Capdevila et al. 2000). These fatty acid epoxides are rapidly hydrolyzed, which leads to the formation of vicinolic dihydroxylated PUFAs. Alternatively, cytochrome P450 enzymes may also catalyze omega- and/or omega-1 oxidation of fatty acid, forming monohydroxylated fatty acid derivatives.

Eicosanoid biosynthesis via these three pathways has been implicated in the pathogenesis of AD, but the underlying molecular mechanisms remain largely elusive. Some eicosanoids exacerbate AD pathology, while others exhibit beneficial effects (Biringer 2019). Unfortunately, for the time being, there is no unifying concept that proves the putative relevance of either of these mediators or of their biosynthesizing enzymes for the pathogenesis of AD. A number of clinical trials have been carried out in AD patients using specific PTGS-2 inhibitors (COXIBS), but all the results obtained so far were negative (Firuzi and Praticò 2006). Except for the isoform-specific ALOX5 inhibitor zileuton (Carter et al. 1991), which has been approved for clinical use as an antiasthmatic drug in the US, there is no other clinically approved ALOX inhibitor available at the moment. Thus, for the time being, it is impossible to perform clinical studies testing the potential effects of isoform-specific ALOX inhibitors in AD patients. However, a number of different knock-out (Martens et al. 2002), (Dobrian et al. 2010), knock-in (Epp et al. 2007; Hallenborg et al. 2010a, b) (Marbach-Breitrück et al. 2021), and transgenic mice are currently available for both the PTGS and ALOX pathway. Unfortunately, most of these animals have not been tested in mouse models of AD. The lack of such in vivo experimental data may also be related to the fact that the currently available mouse models of AD are rather complex and do not mirror all major aspects of human AD (Yokoyama et al. 2022). Unfortunately, the eicosanoid metabolism of APOE2, APOE3, and APOE4 transgenic mice has not been studied in detail, and thus, it remains unclear how overexpression of these human alleles may impact systemic eicosanoid biosynthesis.

In humans, the APOE genotype modifies the plasma oxylipin response to omega-3 polyunsaturated fatty acid supplementation in healthy individuals (Plourde et al. 2009; Saleh et al. 2021). Since these fatty acids constitute substrates for the biosynthesis of eicosanoid and related oxylipins, the pattern of these lipid mediators should also be modified. Unfortunately, the authors have not explored whether this might also be the case in AD patients. On the other hand, post-mortem oxylipidome analyses in the dorsolateral prefrontal cortex suggested lower levels of omega-3 fatty acid-derived neuroprotectin D1 in patients suffering from AD dementia. Here, a significant interaction was observed between the presence of the APOE4 allele and the levels of both pro-inflammatory and pro-resolving oxylipins on readout parameters of cognitive performance and on plaque burden (Ebright et al. 2022). Furthermore, analysis of lipid metabolizing pathways suggested an activation of calcium-dependent phospholipase A2, 5-lipoxygenase, and soluble epoxide hydrolase (Ebright et al. 2022). Since these enzymes play a major role in eicosanoid metabolism, activation of eicosanoid biosynthesis in AD might be concluded. Unfortunately, in this experimental approach, the relative contribution of post-mortem alterations is hard to exclude.

#### Endocannabinoids and APOE4 in the Pathogenesis of AD

Cannabinoids are exogenous chemical substances that bind with high affinity to the major cannabinoid receptors (CB1, CB2) of the human body. They induce similar effects as tetrahydrocannabinol (THC), which is synthesized in large quantities by *Cannabis sativa* (Abyadeh et al. 2021). Although this plant produces up to 100 chemically distinct cannabinoids, the two major compounds are THC and cannabidiol (CBD), which are responsible for the psychogenic effects of cannabis (Hazekamp et al. 2010). Since THC induces strong psychoactive effects (Bhattacharyya et al. 2009) whereas CBD is mainly antipsychoactive, the mass ratio of these two chemicals is decisive for the psychogenic effect of a cannabis plant. CBD also reduces some other negative effects of THC such as anxiety (Crippa et al. 2004). Endocannabinoids are endogenous substances in the human body that also bind to CB1 and/or CB2 and induce similar psychogenic effects as THC. In the human body, two major endocannabinoids [anandamide (AEA) and 2-arachidonylglycerols (2-AG)] have been identified, and both of them carry an arachidonic acid residue. They have been implicated in neuronal function but also play a role in the regulation of other physiological processes such as pregnancy, cell development, immune-response, appetite regulation, pain sensation, mood, and memory. In the central nervous system, endocannabinoids play important roles in the control of movement and motor coordination, in learning and memory, and in emotion and motivation. Moreover, as endorphins, endocannabinoids function as endogenous analgesics (Kaur et al. 2016).

On the molecular level, endocannabinoids function as retrograde neurotransmitters. These compounds are synthesized by presynaptic neurons and subsequently released into the synaptic space. There, they bind to the CB receptors localized in the presynaptic membrane and downregulate the further release of other neurotransmitters. In other words, they prevent neuronal hyperactivity. Since the endocannabinoid system is highly expressed in the hippocampus and in the cortex, endocannabinoids have been associated with learning and memory. These cerebral functions are frequently defective in AD patients, and it has been suggested that AD patients carry a compromised endocannabinoid system (Basavarajappa et al. 2017). Moreover, recent findings in AD rodent disease models have shown that cannabinoids are capable of reducing amyloid plaque formation and stimulate hippocampal neurogenesis. Beneficial effects of cannabinoids on other dementia-related symptoms have also been reported in clinical trials. Accordingly, future studies should be focused on optimizing the therapeutic dosages and the time protocol for the use of cannabinoids as drugs in AD therapy (Coles et al. 2022).

Endocannabinoids are typically broken down by unspecific hydrolases, which include fatty acid amide hydrolase and monoacylglycerol lipase. However, they also serve as substrates for cyclooxygenases (Urquhart et al. 2015), cytochrome P450 isoforms, and lipoxygenases (Ivanov et al. 2021). These enzymes oxygenate the arachidonic acid backbone, which leads to the formation of an entirely novel array of bioactive lipids (Schwitter et al. 2023). The bioactivities of these oxygenated endocannabinoids are different from those of the non-oxygenated parent compounds, and thus, enzymatic oxygenation modifies the functionality of the endocannabinoid system, which contributes to the pathogenesis of AD (Abate et al. 2021).

ApoE binds to the cell surface receptor sortilin (Xu et al. 2018), which mediates uptake of apoE-bound lipids into neurons. Unfortunately, the significance of this pathway for the APOE4-related AD risk has not been explored in detail. Recently, it has been shown that sortilin directs the uptake and conversion of polyunsaturated fatty acids into endocannabinoids, which upregulate the expression of neuroprotective genes in the brain (Asaro et al. 2020). This signaling activity of sortilin requires APOE3, but is disrupted by binding of APOE4, which compromises neuronal endocannabinoid metabolism and action. The authors concluded that the APOE cell surface receptor sortilin facilitates neuroprotection which is disrupted in APOE4 carriers (Asaro et al. 2020).

#### Sphingolipids and APOE4 in the Pathogenesis of AD

Sphingolipids form a class of biologically active lipids that are important constituents of biomembranes (Loewith et al. 2019) but also play a role as lipid signaling molecules (Kleuser 2018). They have been implicated in the pathogenesis of AD (Czubowicz et al. 2019), but the pathophysiological mechanisms have not been explored in detail (Hannun and Obeid 2018). Disruption of sphingolipid metabolism can result in the accumulation of specific sphingolipid species including ceramides and sphingosine-1-phosphate (S1P), which have been implicated in AD (Han et al. 2011). Ceramides have been linked to increased Aß levels (Dehghan et al. 2022). Sphingolipids have also been implicated in the immune response and in neuroinflammation, which are prominent aspects of AD (Maceyka and Spiegel 2014). S1P upregulates activation and migration of cerebral microglia and thus contributes to chronic cerebral inflammation (Qi et al. 2022), which may exacerbate neurodegeneration in AD (Maceyka and Spiegel 2014).

As indicated above, sphingolipids are functionally relevant constituents of the plasma membranes of all mammalian cells including neurons. Since these lipids are usually devoid of polyunsaturated fatty acids, they reduce membrane fluidity (McGonigal et al. 2019). An increase in the sphingolipid content of a membrane reduces membrane fluidity making the membrane less flexible. In neurons, this may lead to neuronal dysfunction, which is a common feature of AD pathogenesis (Piccinini et al. 2010). Moreover, recent studies suggest that sphingolipids might directly impact tau pathology. Specific ceramides have been associated with tau hyperphosphorylation, which is a key step in the formation of NFTs (Randez-Gil et al. 2020). Sphingolipids are integral components of lipid rafts in cell membranes. Lipid rafts are known to be involved in the processing and accumulation of Aβ (Taylor and Hooper 2007). Disruptions in lipid rafts due to changes in sphingolipid composition could potentially lead to increased Aβ production and aggregation (Pham and Cheng 2022). Although elevated serum levels of sphingolipids have been identified as biomarker for AD (Agarwal and Khan 2020), a possible relation to the APOE4 expression remains to be explored. Nevertheless, recognizing the involvement of sphingolipids in the pathogenesis of AD could have preventive and therapeutic implications. Targeting sphingolipid metabolism and their downstream signaling pathways may offer new approaches to slow down or prevent the progression of AD (Hannun and Obeid 2018). However, the potential role of sphingolipids in the pathogenesis of AD is complex and evolves in a large number of areas of research. Understanding how these lipid molecules interact with other key factors in AD, such as Aβ, tau, and neuroinflammation, is essential for advancing our knowledge of the disease and developing effective treatments in the future.

### APOE4 and Neuroinflammation in the Pathogenesis of AD

Neuroinflammation is one of the major processes in the pathogenesis of AD (Lecca et al. 2022), and APOE has been characterized as an inducer of neuroinflammation. APOE4 plays a major role in the induction of the innate immune response in the brain of AD patients, but the molecular mechanisms are apparently very complex (Ophir et al. 2005; Cash et al. 2012; Dorey et al. 2014; Du et al. 2015). One of the major pro-inflammatory effects of APOE4 is activation of microglia, which subsequently induces neuroinflammation (Rodriguez et al. 2014; Li et al. 2015). In this context, miRNA146a, which is present in the brain in large amounts, has been implicated in APOE4-induced neuroinflammation. In fact, AD patients have higher levels of miRNA146a, which leads to chronic inflammation and involves inadequate negative feedback regulation (Lukiw et al. 2008; Teter et al. 2016). When neurons were incubated in vitro with different APOE isoforms, they secreted the classical pro-inflammatory cytokine interleukin-1ß and the extent of cytokine secretion was significantly higher when APOE4 was used instead of APOE3 (Guo et al. 2004; Dorey et al. 2014). When transgenic mice overexpressing alternatively either APOE4 or APOE3 were treated by intracerebroventricular injection of lipopolysaccharide (LPS) as inflammation inducer, expression patterns of inflammation-related gene products were similar, but the extent of pro-inflammatory gene expression was significantly elevated and more prolonged in APOE4 mice (54). Detailed clustering analysis of the expression patterns indicated predominant expression of genes carrying NF-κB response elements. Direct quantification of the expression of NF-κB-regulated genes revealed that the extent of activation was more pronounced in APOE4 mice when compared with APOE3 animals. These findings suggested that the higher degree of neuroinflammation in APOE4 mice may be related to dysregulation of NF-κB signaling (Ophir et al. 2005). In a medium-scale (some 2500 patients) epidemiological study, the impact of chronic low-grade peripheral inflammation in *APOE4* allele carriers was tested on the onset of AD (Tao et al. 2018). Elevated plasma levels of C-reactive protein (CRP) shortened the latency for the onset of clinical AD symptoms, and the authors concluded that treating chronic systemic inflammation based on genetic risk might be considered an effective prevention method for premature development of AD symptoms.

In addition to APOE4, the triggering receptor expressed on myeloid cells 2 (TREM2) has been genetically linked to AD (Golde et al. 2013). Although the molecular basis for the genetic association has not been explored in detail, it was suggested that APOE4 may directly bind to TREM2 and thus alter TREM2 signaling. APOE isoforms function as TREM2 agonists with an EC_50_ in the low nM range, but the differences between the APOE isoforms were not particularly impressive (Jendresen et al. 2017). Although the binding was displaced by an APOE-mimetic peptide, the lack of isoform specificity does not provide strong evidence for the involvement of these mechanisms in AD pathogenesis.

Neuroinflammation is a complex process and detrimental as well as beneficial neuroinflammatory phenotypes (NIP) have been reported (Tai et al. 2015). Although many studies have shown that APOE4 induces a detrimental phenotype of neuroinflammation, this overall effect might be related to a suppression of the beneficial mechanisms of neuroinflammation. APOE4 increases Aβ-induced pro-inflammatory receptor signaling (toll-like receptor 4-p38α), but it may also suppress beneficial receptor-mediated pathways (IL-4R nuclear receptor). Dysregulation in sphingolipid metabolism can lead to the buildup of distinct sphingolipid species, such as ceramides and sphingosine-1-phosphate (S1P), which have been associated with AD.

### APOE4 and Synaptic Plasticity in the Pathogenesis of AD

Interneuronal communication is one of the major functional features of the human brain, and this communication proceeds via synapses. The human brain involves some 10^14^ synapses and a single neuron carries between 1 and 200,000 of them. Synaptic connections facilitate the exchange of information not only between neurons but also between neurons and astrocytes (Perea et al. 2009). The activity of synapses is strengthened or weakened over time depending on the intensity of the flux of information, and this regulatory process is called synaptic plasticity. Since memory and learning are represented by interconnected neuronal circuits, synaptic plasticity is one of the most important neurochemical bases for these higher brain functions. There are several mechanisms to improve synaptic plasticity including alterations in the quantity of neurotransmitter release (Gaiarsa et al. 2002). ALOX polymorphism impacts synaptic plasticity. The regulation of axon or dendrite outgrowth, which is crucial for synaptic plasticity, is influenced by APOE isoforms. In general, APOE-containing lipoproteins protect neurons from apoptosis via low-density lipoprotein receptor-related protein-1 (LPRP1)-mediated pathways (Hayashi et al. 2007). However, APOE3 activates LRP1 signaling more effectively than APOE4 (Sen et al. 2012). Moreover, APOE isoforms show differential effects on neurite outgrowth. APOE3 activates dendrite outgrowth, whereas APOE4 inhibits this process (Mahley et al. 1996).

## Ferroptosis

As apoptosis and necroptosis, ferroptosis is a regulated suicide pathway (Galluzzi et al. 2018), but mechanistically, it must be separated from the other types of regulated cell death (Yang and Stockwell 2016). It has been implicated in physiological killing of tumor cells but also in the pathogenesis of different diseases with high socio-economic impact including neurodegeneration (Stockwell 2022; Sun et al. 2022). Ferroptosis involves three major metabolic hallmarks, which do not play major roles in other cellular suicide mechanisms:

(i) Excessive lipid peroxidation: oxidation of membrane phospholipids carrying polyunsaturated fatty acids (PUFAs) leads to the formation of highly reactive lipid peroxides, which are either reduced to less reactive hydroxy lipids by glutathione peroxidases or undergo secondary decomposition reactions leading to the formation of free lipid radicals. These radicals can subsequently react with other membrane constituents and induce a cascade of secondary oxidation reactions that impair the barrier function of membranes, but also lead to functional impairment of membrane-bound receptors, enzymes, and ion channels. As secondary products of radical-mediated lipid peroxidation, reactive aldehydes, such as malondialdehyde (MDA) and hydroxynonenal (HNE), are formed. These compounds may further react with amino groups of proteins and nucleic acids to cause structural and functional modification (Dixon et al. 2012).
(ii) Impairment of lipid peroxide reduction: under aerobic conditions, peroxidation of unsaturated lipids is a normal process, and excessive accumulation of lipid peroxides is usually prevented by hydroperoxy lipid-reducing enzymes. Glutathione-dependent and glutathione-independent reduction mechanisms have been described, and glutathione peroxidase 4 (GPX4) is one of the major players in controlling the cellular lipid peroxide tone. This enzyme reduces complex hydroperoxy lipids to less reactive alcohols at the expense of reduced glutathione (GSH) and/or alternative electron donors (Dixon et al. 2012). When GSH is used as a reductant, glutathione disulfide (GS-SG) is formed that needs to be back-reduced via the GSH-reductase reaction, and this pathway requires sufficient amounts of reduced NADPH_2_, which mainly originates from the oxidative pentose shunt (Saha et al. 1992). A deficiency in catalytically active GPX4 impairs the cellular reductive capacity, which results in uncontrolled intracellular accumulation of lipid hydroperoxides (Dixon and Stockwell 2019).
(iii) Intracellular iron deposition: biologically active transition metals such as iron, copper, cobalt, or manganese are capable of interacting with intracellular peroxides catalyzing the homolytic breakdown of the hydroperoxy bond (RO-OH). This reaction (Fenton reaction) forms free radical intermediates (alkoxy radicals and hydroxy radicals), which can induce secondary oxidation reactions which might lead to the dysfunction of biomolecules. In ferroptosis, accumulation of intracellular iron ions triggers an elevated production of free radicals, potentially surpassing the cellular reductive capacity. Under these conditions, the cellular redox state might irreversibly be disturbed, which drives the cells into ferroptotic cell death to avoid damage of surrounding cells and constituents of the extracellular matrix (Dixon et al. 2012).

### Ferroptosis and Alzheimer’s Disease

AD is a neurodegenerative disorder that is characterized by excessive iron accumulation in the brain (Küpper et al. 2017). Such iron deposits are found in the extracellular space, where the iron interacts with Aβ peptides (Perry et al. 2002) but also in the neurons. Until recently, cerebral iron deposition has mainly been discussed as inducer of oxidative stress (Castellani et al. 2007). This is still a valid interpretation, but in light of the recent findings that intracellular iron accumulation is a key process in ferroptotic signaling, alternative functional consequences of iron accumulation need to be considered. Neuronal ferroptosis has been suggested as a key process in the pathogenesis of AD, and the pathophysiological consequences of ferroptotic cell death in the development of AD have recently been reviewed (Chen et al. 2021b; Jakaria et al. 2021; Wang et al. 2022; Zhang et al. 2022; Zhao et al. 2023). In these papers, the authors also discussed novel therapeutic concepts targeting AD-related cerebral iron deposition to prevent the functional defects associated with ferroptotic cell death. However, for the time being, the usefulness of these innovative therapeutic strategies is still a matter of discussion and clinical studies must be carried out to test their effectiveness.

Sirtuins are highly conserved NAD^+^-dependent enzymes that have been implicated in the pathogenesis of AD and other age-related diseases. In humans, there are 7 different sirtuin isoforms (SIRT1-7), which are localized either in the cytosol, in the mitochondria, and/or in the nucleus (Lalla and Donmez 2013). Most of them exhibit deacetylase activities and regulate the expression and catalytic activity of a large number of gene products, which play important roles in metabolism, energy homeostasis, and longevity (Houtkooper et al. 2012). Overexpression of SIRT1 exhibits protective effects against the development of neurological symptoms in AD models, and thus, designing therapeutics based on SIRT1 activity might be useful to develop treatment methods for this disease (Lalla and Donmez 2013). In fact, AD patients might benefit from the consumption of SIRT1 activators but should avoid the intake of SIRT1 inhibitors. Thus, understanding and modulating SIRT1 activity might be crucial in AD management (Campagna et al. 2018). In contrast to APOE3, APOE4 significantly reduces Sirt1 expression and modifies the ratio of neuroprotective Sirt1 to neurotoxic Sirt2. It also triggers phosphorylation of Tau and APP and induces programmed cell death (Oliveira and Klann 2022). This data indicates that apoE4 impacts the delicate balance of sirtuin isoforms in the context of neuroprotection and opens innovative strategies for the treatment of AD. Moreover, APOE4 exhibits a higher binding affinity for Sirt1 when compared with APOE3 and APOE2 (Lima et al. 2020).

As indicated above, the antioxidative enzyme glutathione peroxidase 4 (GPX4) reduces hydroperoxy phospholipids and its deficiency induces ferroptosis (Yang et al. 2014). However, a lack of GPX4 expression and/or inhibition of the catalytic activity of this enzyme is not the only mechanism involved in ferroptosis. In fact, dysregulated iron homeostasis is also of pathophysiological relevance (Yang and Stockwell 2008; Gao et al. 2015). Together, intracellular iron accumulation and a lack of lipid peroxide-reducing capacity will induce oxidative stress and subsequent protein misfolding and aggregation (Ashraf and So 2020).

Since the brain is one of the most lipid-rich organs of the human body and since brain cells are rich in PUFAs, the susceptibility of the brain to lipid peroxidation is particularly high. Peroxidation of PUFAs by transition metals (Benedet and Shibamoto 2008) or lipid peroxidizing enzymes induces oxidative stress, which is counteracted by antioxidant enzymes (Savaskan et al. 2007; Cardoso et al. 2017). In AD brains, lipid peroxidation products such as MDA, acrolein, F_2_-isoprostanes, and 4-hydroxynoneal (HNE) have been detected at elevated levels in parallel with Aβ peptide plaques (Vinothkumar et al. 2017). In some studies, it has even been shown that Aβ peptides promote lipid peroxidation, although the underlying mechanisms have not been identified (Selley et al. 2002). Moreover, HNE-protein adducts were detected in large quantities in the brains of AD patients (Bruce-Keller et al. 1998; Liu et al. 2005; Roberts et al. 2012).

### APOE4 and Ferroptosis

Although the major function of APOE is its role in lipid transport (Mahley et al. 1984), it also impacts neuronal iron homeostasis (Kagerer et al. 2020). APOE4 modifies the iron affinity of apoferritin, which is a major intracellular iron-binding protein (Ayton et al. 2015a, b). In cells, iron is essential for a number of basic processes. It functions as a cofactor for oxidoreductases, and thus, it is essential for the functionality of the respiratory chain. Moreover, iron is needed for the synthesis of neurotransmitters (Singh et al. 2014). Thus, dysregulation of neuronal iron homeostasis affects neuronal communication, which is needed for regular brain functionality (Guo et al. 2018). Excessive neuronal iron loading impairs Aβ clearance via APOE-dependent mechanisms (Goodman 1953; Van Bergen et al. 2016). Furthermore, an iron response element (IRE) has been detected in the 5′-untranslated region of the human *APP* mRNA, and thus, expression of the APP protein appears to be upregulated by iron on the translational level. In other words, increased intracellular iron concentrations stimulate the expression of APP mRNA and thus may enhance Aβ formation (Rogers and Lahiri 2004). In contrast, elevated iron concentrations downregulate the expression of the furin protein, responsible for activating β-secretase and subsequently initiating the amyloidogenic pathway in amyloid precursor protein (APP) metabolism (Ward et al. 2014). Excess of intracellular iron deposition can induce amyloidogenesis and Aβ generation via the interaction of the ferritin light chain with the presenilin enhancer-2 (PEN2), a component of the gamma-secretase (Li et al. 2013). Ferritin itself can bind to Aβ peptides initiating Aβ fibril formation (Balejcikova et al. 2018). As described before, neurofibrillary tangles (NFT) are characteristic morphological substrates of AD. NFT deposits involve iron (Smith et al. 1997), and iron overload in neurons induces hyperphosphorylation of the tau protein (Shin et al. 2003).

Under normal conditions, the iron homeostasis of brain cells is well regulated. Fe^2+^ enters the brain by crossing the BBB via the divalent metal ion transporter 1 (DMT1). In brain cells, Fe^2+^ activates different types of protein kinases, such as glycogen synthase kinase-3β (GSK3β) and cyclin-dependent protein kinase 5 (CDK5). When activated, these protein kinases hyperphosphorylate tau proteins in a time-dependent manner, which initiates TNF formation (Guo et al. 2013). The initial steps of tau hyperphosphorylation are catalyzed by CDK5, whereas GSK3β is responsible for the later phosphorylation steps. Thus, the concerted interaction of the two protein kinases leads to TNF formation (Hanger et al. 2009; Guo et al. 2013).

APOE is a potent inhibitor of ferroptosis. It activates the PI3K/AKT pathway and inhibits autophagic degradation of ferritin and thus averts iron-dependent lipid peroxidation (Belaidi et al. 2022). Quantifying the iron content in *post-mortem* samples of inferior temporal brain cortex, it was found that the association of iron with clinical symptoms of AD was stronger in patients carrying the *APOE4* allele. Since protection against ferroptosis did not differ between APOE isoforms in vitro, other features of APOE4 carriers, such as a low abundance of APOE protein expression and/or higher levels of PUFAs, could mediate the higher risk of *APOE4* allele carriers for AD (Belaidi et al. 2022).

APOE4 also increases the risk of vascular dementia and atherosclerosis (Mahley et al. 2009; Rohn 2014), and thus, cerebral atherosclerosis may play an important role in the development of AD. When the ferroptosis inhibitor ferrostatin-1 (Fer-1) was administered to ApoE^−/−^ mice, the degree of lipid deposition in the artery walls was reduced. Moreover, Fer-1 normalized the expressions of ferroptosis indicators such as SLC7A11 and glutathione peroxidase 4 (GPX4). These data suggest that ferroptosis might play a role in atherogenesis (Bai et al. 2020) and that AD patients might benefit from the systemic application of ferroptosis inhibitors.

**Fig. 4:**
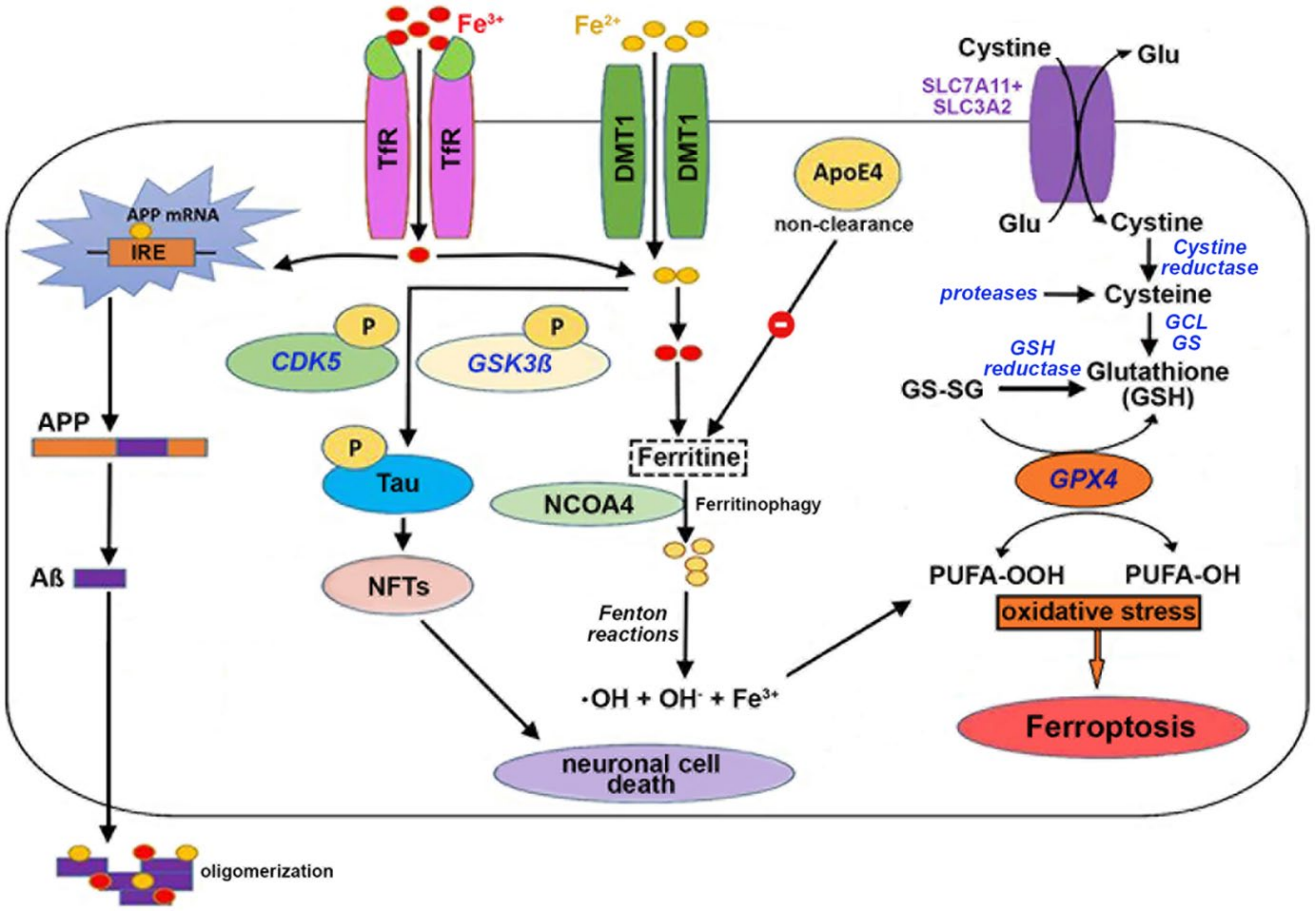
The interplay of the APOE4 protein, lipid peroxidation, and ferroptosis in the pathogenesis of Alzheimer’s disease. Iron ions cannot penetrate cellular membranes without the help of cell surface transporters. As in most other cells, neurons take up Fe^3+^ ions mainly via the transferrin receptor pathway (TfR). Alternatively, Fe^2+^ ions may enter neurons via the divalent metal ion transporter 1 (DMT1). Intracellularly, iron may function as ferrous (Fe^2+^) or ferric (Fe^3+^) ions. Ferrous iron may bind to the iron responsive element localized in the 5′-untranslated region of the APP mRNA, upregulating the expression of the APP gene and stimulating the formation of Aβ and its extracellular oligomerization. In other words, iron accumulation inside neurons upregulates amyloidogenesis. Furthermore, excessive presence of Fe^2+^ triggers phosphorylation of glycogen synthase kinase 3β (GSK3β) and cyclin-dependent protein kinase 5 (CDK5) and these reactions induce hyperphosphorylation of tau proteins and intracellular formation of neurofibrillary tangles (NTFs). NFTs trigger neuronal dysfunction and cell death. The redox-sensitive amino acid cysteine, which is an essential constituent of the glutathione (GSH)/glutathione disulfide (GS-SG) antioxidative defense system, enters neurons via the solute carrier 7A11 (SLC7A11) antiporter, which exchanges the amino acids glutamate (Glu) and cystine. Intracellularly, cystine is reduced to cysteine (Cys) by cystine reductase that uses NADH as electron donor. Cysteine may also originate from proteolytic breakdown of intracellular protein. Subsequently, Cys is used as substrate for glutathione (GSH) synthesis, which proceeds via two consecutive steps of amino acid transferase reaction. Both reactions are ATP-dependent and require gamma-glutamate-cysteine ligase (GCS) and glutathione synthase (GS) as catalyzing enzymes. Alternatively, GSH is formed from glutathione disulfide (GS-SG) via the glutathione reductase reaction, which employs NADPH as electron donor. Reduced glutathione (GSH) is the most frequently employed electron source for the GPX4 reaction, which reduces lipid hydroperoxides (PUFA-OOH) to the corresponding alcohols (PUFA-OH). When GSH is limiting hydroperoxy lipids accumulate and excessive iron deposition induces oxidative stress and ferroptosis. This image was modified from (Sun et al. 2022)

Ferritin is an intracellular iron storage protein. More than 50% of the molecular mass of iron-loaded ferritin is related to its iron content. In this protein, iron is present mainly as ferric hydroxide [Fe(OH)_3_]. When cellular ferritin levels exceed critical values, ferritin is autophaged via a process called ferritinophagy. This mechanism involves the cellular lysosomes and liberates free ionic iron. This iron subsequently accelerates ferroptosis (Mancias et al. 2014; Hou et al. 2016; Wang et al. 2016). The nuclear receptor coactivator 4 (NCOA4) facilitates the transport of dysfunctional iron-loaded ferritin to the autophagosome, orchestrating its subsequent lysosomal degradation (Mancias et al. 2014). The ferritin levels in the cerebrospinal fluid (CSF) are positively correlated with the CSF APOE4 levels, and APOE4 carriers have significantly elevated liquor levels than APOE3 carriers (Ayton et al. 2015a, b). Moreover, CSF ferritin concentration does negatively correlate with cognitive function (Ayton et al. 2018). In conclusion, iron metabolism and ferritinophagy are two key points in controlling ferroptotic cell death (Fig. 4), and thus, they are of pathophysiological relevance for the development of AD.

### Ferroptosis Inhibitors as Potential Anti-AD Drugs

Since ferroptosis has recently been implicated in the pathogenesis of AD (Jakaria et al. 2021; Sun et al. 2022), ferroptosis inhibitors may be considered as potential anti-AD drugs. The potential relevance of ferroptosis inhibitors for the treatment of different types neurological diseases including AD has previously been reviewed (Ratan 2020). Among the most promising ferroptosis inhibitors are bioactive natural compounds (Zhao et al. 2023) but also synthetic low molecular weight chemicals functioning as iron chelators (Bruzzese et al. 2023), inhibitors of enzymatic (Kakularam et al. 2022) and non-enzymatic lipid peroxidation (Yin et al. 2011), and general antioxidants (Zhang et al. 2022; Faraji et al. 2024). There are, however, a number of problems associated with systemic application of ferroptosis inhibitors, and two of these problems are briefly discussed below:

(i) Ferroptosis has originally been discovered as a special type of cell death in tumor biology, and several mechanisms contribute to their antitumor activity (Lei et al. 2022). Ferroptosis is a part of several tumor suppressor mechanisms (Jiang et al. 2015), and ferroptosis evasion contributes to tumor initiation, tumor progression, and metastasis (Ubellacker et al. 2020) as well as to therapeutic tumor resistance (Zhang et al. 2022). Some types of cancer are intrinsically susceptible to ferroptotic cell death, and this vulnerability could be therapeutically targetable in certain types of cancer (Zou et al. 2019). Moreover, in order to survive, some cancer cells strongly depend on antiferroptosis defense systems under metabolic stress and disruption of these defense mechanisms would be fatal to such cancer cells (Mao et al. 2021). Finally, ferroptosis is triggered by a number cancer therapies (Lei et al. 2020), and thus, ferroptosis inducers have a great therapeutic potential. On the other hand, ferroptosis inhibitors, which might slow down neuronal cell death during the pathogenesis of AD, are likely to induce malignant transformation, tumor progression, and metastasis as unwanted side effects, and thus, according to our current knowledge on the specificity of ferroptosis inhibitors, the use of such compounds as anti-AD drugs may not be justified.
(ii) Lipid peroxidation is a key process in ferroptosis and different lipoxygenase isoforms (Ivanov et al. 2015), in particular ALOX15 and ALOX5, have been implicated (Chen et al. 2021a, b; Stockwell 2022). In other words, ALOX inhibitors might interfere with ferroptotic signaling. However, ALOX isoforms have a number of functions outside ferroptosis (Haeggstrom and Funk 2011; Kuhn et al. 2015), and thus, there is the danger of unwanted side effects. For instance, ALOX15 plays a role in the maturational breakdown of mitochondria during the maturation of red blood cells (Schewe et al. 1986) and humanization of the reaction specificity of mouse Alox15 leads to dysregulated erythropoiesis (Reisch et al. 2023). Moreover, ALOX15 has also been implicated in the degradation of other intracellular organelles (van Leyen et al. 1998) and in a humber of additional physiological processes (Ford-Hutchinson 1991). Similarly, functional ALOX12, ALOX12B, and ALOXE3 are required for normal epidermal differentiation (Krieg and Furstenberger 2014) and functional inactivation of the corresponding genes (Johnson et al. 1999; Epp et al. 2007; Krieg et al. 2013) induces excessive epidermal water evaporation and thus leads to systemic dehydration. In other words, the application of lipoxygenase inhibitors to AD patients might interfere with a number of physiological processes and thus may induce a number of unwanted side effects.

## Conclusions and Perspectives

Alzheimer’s disease (AD) is one of the most prevalent neurodegenerative disorders, and many risk factors have been identified for this disease. It is characterized by premature neuronal death, but our mechanistic understanding of the molecular processes leading to cell death remained unclear for a long time. Epidemiological studies suggested that polymorphism at the APOE gene locus is one of the most relevant risk factors for AD, but how this genetic polymorphism induces cell death remained a mystery for many years. Today, we know that the polymorphism of the *APOE* gene, in particular the presence of the APOE4 allele, alters neuronal iron homeostasis leading to excessive intracellular iron deposition. These iron deposits induce oxidative stress, which leads to uncontrolled lipid peroxidation. Under normal conditions, the hydroperoxy lipids are rapidly reduced to the corresponding hydroxy derivatives by the catalytic activity of glutathione peroxidases. However, when the cellular reductive capacity is impaired, the hydroperoxy lipids are decomposed via free radical-mediated secondary reactions and products of these secondary reactions may trigger ferroptotic signaling leading to neuronal cell death. Pharmacological interference with the intracellular ferroptotic signaling is likely to prevent neuronal ferroptosis, and thus, AD patients might benefit from such therapeutic intervention. Unfortunately, for the moment, no neuron-specific ferroptosis inhibitors are available but the search for such compounds is underway. For the development of such inhibitors, it should always be kept in mind that intervention of ferroptotic signaling might be dangerous since ferroptosis has been implicated in killing cancer cells and thus the use of unspecific ferroptosis inhibitors might activate tumor development and metastasis.

## List of Abbreviations

According to the recommendations of the Nomenclature Committee of the International Union of Biochemistry and Molecular Biology, proteins should be named according to encoding genes as long as the genes mainly encode for a single protein. The names of genes or of corresponding alleles should be italicized (*APOE4*) whereas the encoded proteins should be named in normal face (APOE4). Human genes and proteins should be given in capital letters (*APOE4* for the gene, APOE4 for the protein), whereas the mouse orthologs should be labeled in small letters (*apoE4* for the mouse gene, apoE4 for the mouse protein). If the species are not defined, the human nomenclature should be used (capital letters).

Alzheimer disease, AD; Apolipoprotein E, APOE; beta-amyloid, Aβ; neurofibrillary tangles, NFT; early-onset form of AD, FAD; presenilin 1, *Psen1*; presenilin 2, *Psen2*; amyloid precursor protein, APP; Late onset-AD, LOAD; low-density-lipoprpotein-receptor, LDLR; ATP-binding assette A1, ABCA1; low-density lipoprotein receptor-related protein-1, LRP1; heparin sulphate proteoglycan, HSPG; blood-brain-barrier, BBB; Insulin-degrading enzyme, IDE; angiotensin converting enzyme, ACE; neprilysin, Nep; receptor for advanced glycosylation end products, RAGE; tau-proteins, tubulin associated units; microtubule associated protein tau, *MAPT*; extracellular signal-regulated kinase, ERK; eicosapentaenoic acid, EPA; docosahexaenoic acid, DHA; Mitochondria-associated membranes, MAM; endoplasmic reticulum, ER; Prostaglandin G synthase, PTGS; cyclooxygenase, COX; Arachidonic acid lipoxygenase, ALOX; major cannabinoid recepotors, CB; tetrahydrocannabinol, THC; cannabidiol, CBD; anandamide, AEA; 2-arachidonylglycerols, 2-AG; sphingosine-1-phosphate, S1P; C-reactive protein, CRP; triggering receptor expressed on myeloid cells 2, TREM2; neuroinflammatory phenotypes, NIP; vascular endothelial growth factor, VEGF; low-density lipoprotein receptor-related protein-1, LPRP1; mitochondrial permeability transition pore, mPTP; reactive oxygen species, ROS; Cyclophilin D, CypD; positron emission tomography, PET; cerebral metabolic rate for glucose, CMRgl; polyunsaturated fatty acids, PUFAs; malondialdehyde, MDA; hydroxynonenal, HNE; glutathione peroxidase 4, GPX4; glutathione, GSH; iron response element, IRE; presenilin enhancer-2, PEN2; divalent metal ion transporter 1, DMT1; glycogen synthase kinase-3β, GSK3β; cyclin-dependent protein kinase 5, CDK5; nuclear receptor coactivator 4, NCOA4; cerebro-spinal fluid, CSF; glutathione synthase GS; glutamate-cysteine ligase, GCL; enhanced green fluorescent protein, EGFP; PTGS-2 inhibitors, COXIBS; sirtuin, SIRT; solute carrier 7A11, SLC7A11; glutamate, Glu; cysteine, Cys.

## Supplementary Information

Below is the link to the electronic supplementary material.

Supplementary file1 (DOCX 2391 KB)

## Data Availability

No datasets were generated or analyzed during the current study.
