# Supplementary material for: Multiple Roles of Apolipoprotein E4 in Oxidative Lipid Metabolism and Ferroptosis During the Pathogenesis of Alzheimer’s Disease
Source: J Mol Neurosci. 2024 Jul 3;74(3):62. doi: 10.1007/s12031-024-02224-4 (PMC11222241; doi:10.1007/s12031-024-02224-4)
Supplement: Supplementary file 1 — Supplementary file1 (DOCX 2391 KB) [file 12031_2024_2224_MOESM1_ESM.docx]

**Multiple roles of apolipoprotein E4 in oxidative lipid metabolism and ferroptosis during the pathogenesis of Alzheimer’s disease.**

Parisa Faraji ^1,2^, Hartmut Kühn ^2^*, and Shahin Ahmadian ^1^*

^1^Institute of Biochemistry and Biophysics, University of Tehran, Tehran, Iran; [sh.ahmadian@ut.ac.ir](mailto:sh.ahmadian@ut.ac.ir); [Parisa.faraji@ut.ac.ir](mailto:Parisa.faraji@ut.ac.ir)

^2^Department of Biochemistry, Charité-Universitätsmedizin Berlin, Corporate Member of Freie Universität Berlin and Humboldt Universität zu Berlin, Charitéplatz 1, 10117 Berlin, Germany; [hartmut.kuehn@charite.de](mailto:hartmut.kuehn@charite.de); parisa.faraji@charite.de

**Key words:** neurodegeneration, proteostasis, lipid peroxidation, iron homeostasis, glutathione peroxidase 4, free radicals

*Correspondence: hartmut.kuehn@charite.de, Tel.:+49-30-450528040 and [sh.ahmadian@ut.ac.ir](mailto:sh.ahmadian@ut.ac.ir); Tel +98-66956983.

**Abstract (237 words)**

Alzheimer disease (AD) is the most prevalent neurodegenerative disease worldwide and has a great socio-economic impact. Modified oxidative lipid metabolism and dysregulated iron homeostasis have been implicated in the pathogenesis of this disorder but the detailed patho-physiological mechansims still remain unclear. Apolipoprotein E (APOE) is a lipid-binding protein that occurs in large quantities in human blood plasma and a polymorphism of the *APOE* gene locus has been identified as risk factors for AD. The human genome involves three major APOE alleles (*APOE2*, *APOE3*, *APOE4*), which encode for three subtly distinct apolipoprotein E isoforms (APOE2, APOE3, APOE4.) The canonic function of these apolipoproteins is lipid transport in blood and brain but *APOE4* allele carriers have a much higher risk for AD. In fact, about 60% of clinically diagnosed AD patients carry at least one *APOE4* allele in their genomes. Although the APOE4 protein has been implicated in patho-physiological key processes of AD, such as extracellular beta-amyloid (Aβ) aggregation, mitochondrial dysfunction, neuro-inflammation, formation of neurofibrillary tangles, modified oxidative lipid metabolism and ferroptotic cell death, the underlying molecular mechanisms are still not well understood. As for all mammalian cells, iron plays a crucial role in neuronal functions and dysregulation of iron homeostasis has also been implicated in the pathogenesis of AD. Imbalances in iron homeostasis and impairment of the hydroperoxy lipid-reducing capacity induce cellular dysfunction leading to neuronal ferroptosis. In this review, we summarize the current knowledge on *APOE4*-related oxidative lipid metabolism and the potential role of ferroptosis in the pathogenesis of AD. Pharmacological interference with these processes might offer innovative strategies for therapeutic interventions.

**Background**

Alzheimer's disease (AD) is the most prevalent neurodegenerative disorder worldwide. It is characterised by a number of clinically important symptoms such as cognitive malfunctions and loss of memory **(Teng 2024, Vejandla, Savani et al. 2024)**. The name AD was first introduced in 1906 by the German psychiatrist Alois Alzheimer. These days about 70% of all human dementia cases have been related to AD (Yamazaki, Zhao et al. 2019). AD is an age-related disorder and in older individuals its incidence is roughly doubling every 5-10 years **(Yamazaki, Zhao et al. 2019)**. For persons between 65 to 69 years of age a low incidence (0.6 %) has been reported **(Yamazaki, Zhao et al. 2019)**. In contrast, for individuls older than 85 years the incidence is dramatically (8.4%) higher **(Yamazaki, Zhao et al. 2019)**. The prevalence is also increasing with age rising from about 3% in the age group 65-74 years to almost 50% among individuals 85 years or older **(Yamazaki, Zhao et al. 2019)**. In 2020 AD affected about 50 million people in the world but this number is expected to grow because of the demographic changes. Globally, the number of adults of 65 years and older will reach about 973 million in 2030 **(Yamazaki, Zhao et al. 2019)** and thus, much more AD cases will be diagnosed. In other estimates (2023), the global number of persons with AD dementia, prodromal AD, and preclinical AD were 32, 69, and 315 million, respectively. Together they constituted 416 million across the AD continuum, or 22% of all persons aged 50 and above **(Gustavsson, Norton et al. 2023)**. Taking into account that advanced age is the most significant risk factor for AD, one cannot underestimate the socio-economic impact of this disorder **(Zhu and Sano 2006)**.

The most prominent morphological sign of AD is the extracellular deposition of beta-amyloid (Aβ) forming the characteristic amyloid plaques. In addition, intra-neuronal accumulation of hyperphosphorylated tau proteins occurs, which leads to the formation of neurofibrillary tangles (NFT) disturbing the functionality of the neuronal cytoskeleton **(Anand, Gill et al. 2014)**. Although NFT formation has also been reported in other neurodegenerative diseases it is considered a pathological hallmark of AD.

Considering the time-course of symptom development and the patho-genetic mechanisms, two major forms of AD can be distinguished. The early-onset form of AD (FAD), which represents less than 1% of all AD cases, is a genetic disease. It follows an autosomal dominant mode of inheritance and is usually fully developed before the age of 65 years **(Sherrington, Rogaev et al. 1995, Hardy and Selkoe 2002)**. In most cases, FAD is caused by mutations in two different genes: i) The first type of mutations are localised in the genes encoding for the proteins presenilin 1 (*Psen1*) and/or presenilin 2 (*Psen2*). The *Psen1* gene is located in a central region (q24.2-q24.3) of the long arm of chromosome 14. In contrast, the *Psen2* gene was mapped to the distal region (q42.13) of the long arm of chromosome 1. The corresponding proteins are essential for the catalytic activity of gamma-secretase, which plays an important role in the metabolism of the amyloid precursor protein (APP) **(Rogaev, Sherrington et al. 1995, Xu 2009)**. ii) The second type of mutations are localized in the *APP* gene. This gene is located in the central region (q21.3) of the long arm of chromosome 21. The patho-physiologically relevant mutations mainly occur in the region of the *APP* gene that encodes for the recognition sequences of the secreatase proteases. These enzymes hydrolytocally cleave APP forming A$\beta$ cleavage products. These proteolytic fragments are secreted into the extracellular space, aggregate and form the of amyloid plaques **(Zhang, Thompson et al. 2011).**

The major variant of AD is late-onset AD (LOAD), which usually affects patients with an age higher than 65 years **(Haass and Selkoe 2007, Huang and Mucke 2012)**. Although a number of genetic and environmental risk factors have been described for LOAD, the expression of the *APOE4* allele is the major one. More than 15% of all LOAD patients carry this dysfunctional allele at the *APOE* gene locus. Homozygous allele carriers have a 20-fold higher risk for LOAD when compared with carriers of other *APOE* allele combinations **(Corder, Saunders et al. 1993, Association 2018)**. While the precise mechanism by which the APOE4 protein contributes to the pathogenesis of LOAD remains a subject of ongoing debate, recent experiments involving genetically modified mice suggested a strong impairment in the sortilin-dependent neuronal uptake of APOE-lipids. This impairment may be caused by a compromised reshuffling of sortilin to the cellular membrane **(14)**, which reduces fatty acid-binding protein-7-dependent intracellular lipid signalling **(Asaro, Sinha et al. 2021)**.

A cellular hallmark in both forms of AD is premature cell death and investigations into the mechanisms of neuronal cell death have recently suggested that different types of cell death such as apoptosis **(D'Arcy 2019)**, necrosis **(Deroux, Madelon et al. 2022)**, and ferroptosis **(Tang, Chen et al. 2021)** may be involved. Ferroptosis is a non-apoptotic form of regulated cell death that is characterised by dysregulated iron homeostasis and uncontrolled lipid peroxidation **(Li, Cao et al. 2020)**. It has first been implicated in regression of tumour growth **(Tang, Zhu et al. 2020, He, Chen et al. 2021)** but also in neuronal cell death in neurodegenerative diseases **(Reichert, de Freitas et al. 2020, Bao, Pang et al. 2021)**.

Because of the complexity of AD **(Teng 2024)** and because of its socio-economic relevance **(Zhu and Sano 2006)** a large number of researchers have explored different aspects of this disease. Thus, writing a useful review on this disorder is rather challenging. In fact, a PubMed search (March 5th, 2024) with the key words “Alzheimer and review“ revealed 52,367 hits. If a similar search was performed with the key words “Alzheimer and review and APOE” some 1,820 hits were obtained. Finally, we repeated the search with the key words “Alzheimer and review and APOE and ferroptosis“ and here we found two papers **(Plascencia-Villa and Perry 2021, Wang, Li et al. 2024)**. When using the keywords “Alzheimer and review and APOE and ferroptotic“ we did not get any hits. We are well aware of the fact that such keyword-based database searches might be misleading because of several reasons but the low hit numbers encouraged us to write this review. Next, we had a closer look at the two identified papers to find out whether there are thematic ovelaps with our review. In **(Plascencia-Villa and Perry 2021)** preventive and therapeutic strategies for AD are discussed and in **(Wang, Li et al. 2024)** the function of sphingolipids in this disease is reviewed. However, neither of these references addressed the molecular relations between the APOE isoproteins, oxidative lipid metabolism and ferroptosis in the pathogenesis of AD.

**2. Apolipoprotein E and Alzheimer's Disease**

During the past 20 years, numerous large-scale epidemiological studies have indicated that the presence of an *APOE4* allele at the *APOE* gene locus is associated with an increased individual risk for the development of LOAD **(Mamun, Uddin et al. 2020, Sun, Wang et al. 2023)**. In fact, individuals carrying two *APOE4* alleles at this gene locus or an *APOE3+APOE4* allele combination have an increased risk for LOAD **(Serrano-Pozo, Das et al. 2021)**. At least one *APOE4* allele is present in 60% of LOAD patients **(Xian, Pohlkamp et al. 2018)** and the age for the onset of the disease is significantly reduced in *APOE4* allele carriers **(Huang and Mucke 2012)**. On the other hand, individuals carrying *APOE2* or *APOE3* allele combinations are at lower risk **(Zhang and Hong 2015)**. In Table 1 selected roles of APOE in the pathogenesis of AD and the corresponding patho-mechanisms are summarized. Most of them will be discussed in more datil in the text.

**2.1. Apolipoprotein E structures and functions**

Apolipoprotein E (APOE) is a small (34 kDa) protein that consists of 299 amino acids. As with other apolipoproteins (apoliproprotein A, apolipoprotein B48, apolipoprotein B100, apolipoprotein C, etc.) it plays a role in lipid transport in the blood plasma **(Mahley, Innerarity et al. 1984)**. It exhibits anti-atherogenic properties but clearly has additional biological functions **(Ma, Wang et al. 2017)**. In mice, functional inactivation of the apolipoprotein E gene (*apoE* gene) induces athero-susceptibility and *apoE^-/-^* mice develop significant lipid depositions in the arterial wall even when they are maintained on regular low-lipid chow diet **(Song, Tian et al. 2012)**. In earlier studies, it has been reported that *apoE^-/-^* mice have a higher risk for developing AD-related symptoms such as memory defects, tau protein hyperphosphorylation, leaky blood-brain barrier and even Aβ deposits in the brain when compared with wild-type control animals **(Lane-Donovan, Wong et al. 2016, Saul and Wirths 2017, Saroja, Gorbachev et al. 2022)**. However, in more recent studies, the development of such symptoms could not be confirmed **(Long and Holtzman 2019)** and thus, the development of classical AD-related symptoms in *apoE*^-/-^ mice remains controversial.

In humans, the *APOE* gene is located in a central region (q13.32) of the long arm of chromosome 19. It involves 4 exons and 3 introns **(Chawla, Boisvert et al. 2001)**. It is expressed at high levels in the liver, but also in peripheral organs, such as the lungs, kidneys and adipocyte tissue and brain **(Williams, Dawson et al. 1985, Mahley 1988, Ang, Cruz et al. 2008, Getz and Reardon 2009, Huang, Gu et al. 2009)**. In the central nervous system, the major source of the APOE protein are astrocytes and the corresponding protein has been implicated in the cellular import of cholesterol and other lipids needed for basic cell functions. This lipid import is receptor-dependent and requires a functional low-density lipoprpotein receptor (LDLR) **(Liu, Kanekiyo et al. 2013)**. The human APOE protein consists of two distinct structural domains, which are interconnected by a flexible hinge region. The N-terminal domain (amino acid residues 1-167) folds into an antiparallel four-helix bundle. It carries the LDLR binding region between positions 136-150. The C-terminal domain (residues 206-299) consists of three alpha helices. It also involves a lipid-binding site that interacts with the helix bundle in the N-terminal domain **(Momeni and Ferrari 2010, Martínez‐Oliván, Arias‐Moreno et al. 2014, Uddin, Kabir et al. 2019)**.

In humans, the apolipoprotein E gene (*APOE*) is present in three major polymorphic alleles, called *APOE2*, *APOE3* and *APOE4*. These alleles encode three distinct protein isoforms (APOE2, APOE3, APOE4), which differ from each other by single amino acid exchanges. The *APOE2* allele encodes for a protein that carries Cys residues at positions 112 and 158 (Cys112, Cys158). In contrast, the *APOE3* allele encodes for a protein variant carrying a Cys only at position 112 but a positively charged Arg at position 158. The APOE4 protein involves two positively charged Arg residues at the respective positions **(Weisgraber 1994, Liu, Kanekiyo et al. 2013)**. X-ray crystallographic analyses revealed that the presence of a Cys residue at position 112 induces hiding of the positively charged site chain of Arg61 between helix 2 and 3, which is the case for APOE2 and APOE3. On the other hand, a positively charged Arg at this position, which is present in the APOE4 isoprotein, forms a salt bridge with Glu109 and this salt bridge causes exposure of the positively charged Arg61 on the protein surface. In that case **(Figure 1)** Arg61 of the N-terminal domain (helix 2) interacts with the negatively charged site chain of Glu225 of the C-terminal domain and this salt bridge tightens the two APOE4 domains together **(Phillips 2014)**. The postulated interdomain tightening reduces the water solubility of the protein and increases its aggregation behavior.


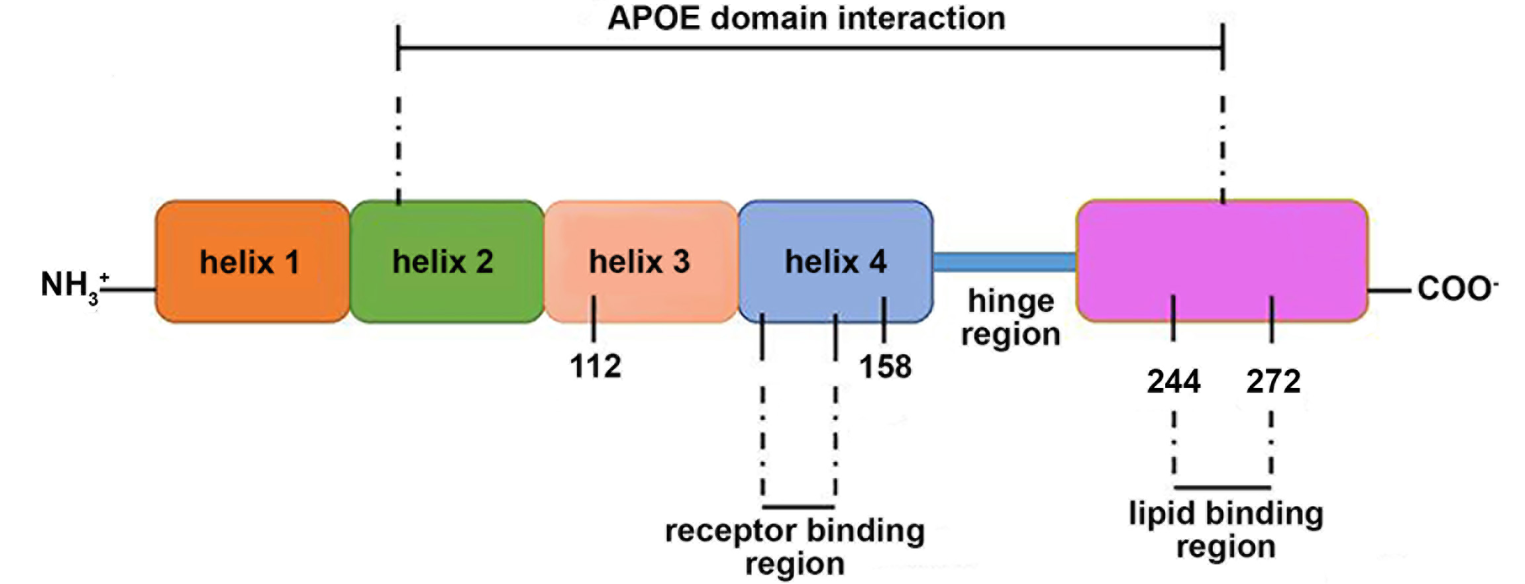


**Figure 1**. A **schematic view of the apolipoprotein E structure.** The APOE protein consists of an N-terminal and a C-terminal domain. The N-terminal domain involves four alpha helices and helix 4 carries the binding region for the LDL receptor. The C-terminal domain involves the lipid-binding subdomain localised between the amino acid residues 244-272. The lipid binding subdomain has also been suggested as binding site for Aß peptides. The two domains are interconnected by a flexible hinge region, which ensures a high degree of structural flexibility for the protein. In the APOE2 protein, the amino acids 112 (helix 3) and 158 (helix 4) are occupied by two Cys residues. In APOE3, a Cys is localised at position 112 but a positively charged Arg is at position 158. The APOE4 protein carries two positively charged Arg residues in these positions. This image was modified from **(Liu, Kanekiyo et al. 2013).**

In the middle European population, 60% of the inhabitants carry two *APOE3* alleles at the *APOE* gene locus. In contrast, in 20-25 % of the inhabitants, one *APOE3* allele and one *APOE4* allele are simultaneously present (heterozygosity) **(Singh, Singh et al. 2006)**. Other *APOE* allele combinations, such as *APOE2+APOE3* (13%), *APOE4+APOE4* (1.7%), *APOE2+APOE4* (1.3%) or *APOE2+APOE2* (0.5%) occur less frequently. It should, however, be stressed that the occurrence frequency of the *APOE4* allele is higher in northern Europe but lower in the south **(Noya and Capurso 1999)**. In Africa, the *APOE4* allele occurs more frequently, but in Asia the *APOE4* allele is distributed with a lower frequency **(Wang, Ge et al. 2021)**.

The 3D-structure of the recombinant human APOE3 protein (PDB 2L7B) has been solved by NMR-studies **(Chen, Li et al. 2011)**. In addition there are a number of crystal structures for different APOE truncation constructs in the PDB database. The C-terminal domain of the human APOE3 protein presents a large exposed hydrophobic surface that likely initiates interactions with lipids, but can also bind Aß peptides. According to the interpretation of the X-raycoordinates by the authors the unique topology of the APOE protein precisely regulates its tertiary structure to permit only one possible conformational adaptation upon lipid- and/or Aß-binding **(Chen, Li et al. 2011)**. It also provides a double security in preventing lipid-free and partially-lipidated APOE from premature binding to APOE receptors during receptor biogenesis. This topology further ensures the optimal receptor-binding activity by the fully lipidated protein during lipoprotein transport in circulation and in the brain. Since lipids and Aß peptides compete for the same binding region the degree of a APOE lipidation modifies the affinity of Aß binding **(Wisniewski and Drummond 2020)**. Since APOE4-containing lipoproteins are less lipidated, APOE4-Aβ complexes are less stable, resulting in reduced intracellular APOE4-Aβ levels and increased Aß accumulation **(Tai, Mehra et al. 2014)**. The binding affinity of both APOE3 and APOE4 for Aß peptides is similar (K_D_ of about 20 nM) but it greatly depends on the conformational state of the Aβ peptide used for the binding studies. Preferential binding was observed when the Aß peptides adopt a β-sheet conformation but there was hardly any difference between APOE3 and APOE4 **(Golabek, Soto et al. 1996)**. Consistent with these results it has also been shown that both APOE3 and APOE4 interact with Aß peptides to form novel monofibrillar structures. Interestingly, APOE4 forms these complex fibrils more avidly **(Sanan, Weisgraber et al. 1994)**.

**2.2. Cerebral expression of APOE and possible biological functions**

As the genes encoding for the other apolipoproteins the different *APOE* alleles are mainly expressed in the liver. To explore the expression of the mouse apoE lipoprotein in more detail reporter mice were constructed, in which the enhanced green fluorescent protein (EGFP) was inserted into the *apoE* gene locus (EGFP-apoE mice). In these animals the EGFP was highly expressed in hepatocytes, in peritoneal macrophages but also in a subset brain astrocytes **(Xu, Bernardo et al. 2006)**. Although normal hippocampal neurons do not express EGFP, kainic acid treatment induced EGFP expression suggesting apoE is expressed in neurons in response to excitotoxic injury. Smooth muscle cells of large blood vessels and cells surrounding small vessels in the CNS did also express the reporter gene **(Xu, Bernardo et al. 2006)**. Unfortunately, whether these findings can be translated into the human situation has not been explored in detail.

To address this point in situ hybridization was carried out on paraffin-embedded and frozen brain sections from three nondemented controls and five AD patients. Using specifically designed antisense *APOE* probes specific in situ hybridization signals were detected glial cells but also in selected neurons in the cerebral cortex and in the hippocampus. In hippocampus, a high density of APOE mRNA-positive neurons were detected in sectors CA1 to CA4 and the granule cell layer of the dentate gyrus **(Xu, Gilbert et al. 1999)**. In cerebellar cortex, APOE mRNA was seen only in Bergmann glial cells and scattered astrocytes but not in Purkinje cells or granule cell neurons. Taken together, these data demonstrated that the APOE mRNA is present at high amounts in human glial cells but also in certain types neurons in the frontal cortex and the hippocampus of humans **(Xu, Gilbert et al. 1999)**. When the APOE4 protein is expressed in neurons it undergoes proteolysis, which results in the generation of neurotoxic fragments. These fragments cause mitochondrial dysfunction and rearrangements in the cytoskeleton. Interestingly, the APOE4 protein exhibits stronger neurotoxic effects than the APOE3 and APOE2 proteins and blocking the interactions between the APOE-domains reversed the detrimental activities **(Mahley and Huang 2012)**.

In macrophages expression of the polymorphic apoE proteins is higly regulated on transcrioptional and post-transcriotional levels **(Larkin, Khachigian et al. 2000)** and similar mechnisms might apply for neurons. Studying expression of the different APOE isoproteins in neuronal cells, a splicing variant of the apoE mRNA was detected that still carries intron-3 of the APOE gene. This splicing variant (APOE-I3) was detected in various neuronal cell lines and in primary neurons of mice and humans. Cell fractionation studies indicated that more than 98% of the APOE-I3 mRNA copies were not exported into the cytososl and thus, they will not be translated into a functional proteins. In transfected primary neurons, APOE expression did increase dramatically when intron-3 was deleted from the transfection construct. These data and the results of additional challenging experiments suggested that neuronal expression of the *APOE* gene under normal conditions is prevented by the minimal nuclear export of the APOE-I3 mRNA. However, in response to cell injury the APOE-I3 mRNA is converted to the mature APOE mRNA, which is rapidly exported into the cytosol and is then translated to the corresponding protein **(Xu, Walker et al. 2008)**.

As other apolipoproteins APOE regulates the cholesterol metabolism in the central nervous system and expression of the APOE4 variant modifies neuronal cholesterol homeostasis. In fact, APOE4 expression elevated endogenous cholesterol synthesis by upregulating the genes encoding for the cholesterol-biosynthesizing enzmes **(Piccarducci, Giacomelli et al. 2023)**. In contrast, the cellular concentrations of acetyl-CoA, which constitutes the major substrate of endogenous cholesterol biosynthesis, were reduced and this was suggested to impact acetylcholine biosynthesis **(Piccarducci, Giacomelli et al. 2023)**. However, it still remains a matter of discussion how exactly the dysregulated cholesterol homeostasis does impact cholinergic communication, neurotoxicity and neuronal death.

**2.3. The role of APOE4 in aggregation of amyloid-beta peptides**

As indicated above, there are two pathological hallmarks in the pathogenesis of AD: i) the formation of extracellular amyloid plaques in the brain; and ii) the formation of intracellular neurofibrillary tangles in neurons **(Hardy and Selkoe 2002)**. Amyloid plaque formation commences with the cleavage of the amyloid precursor protein (APP), one of the most abundantly expressed proteins in the central nervous system **(Aydin, Weyer et al. 2012)**, by specific proteases known as secretases **(Zhang, Thompson et al. 2011)**. APP is present in the plasma membrane of neurons as a transmembrane protein, but it was also detected in the membranes of polarised epithelial cells and non-polarised circulating blood cells. It is metabolised proteolytically *via* two alternative routes called the amyloidogenic and the non-amyloidogenic pathways **(Figure 2)**.

When metabolized *via* the non-amyloidogenic pathway, APP is first cleaved by alpha-secretase and this limited proteolysis releases a soluble N-terminal fragment (sAPPalpha) as well as the C-terminal C83 peptide. After alpha-secretase catalyzed cleavage of APP, the amyloidogenic Aß peptides cannot be formed any more. In other words, after alpha secretase has cleaved APP the formation of the amyloidogenic Aß peptides may not be possible any more and thus, the non-amyloidogenic pathway protects from amyloid plaque formation. Alternatively, in the amyloidogenic pathway, APP undergoes initial cleavage by beta-secretase, resulting in the release of a smaller N-terminal fragment (sAPPbeta). The C-terminal peptide (C99) of the beta-secretase reaction is further cleaved by gamma-secretase and so the full-length β-amyloid peptide (Aβ) is formed. Gamma secretase cleavage takes place in the membrane-spanning domain of the APP protein. Since gamma-secretases do not exhibit absolute regio-specificity, several Aß peptides are created, but Aß_40_ and Aß_42_ are dominant **(Zheng and Koo 2006)**. These peptides are released as monomers into the extracellular space, but here they progressively aggregate, producing protofibrils, fibrils and finally amyloid plaques. Interestingly, compared with Aß40, Aß42 is more prone to aggregation and exhibits a higher degree of neurotoxicity **(El-Agnaf, Mahil et al. 2000)**. Under physiological conditions, the APP is preferentially metabolised *via* the non-amyloidogenic pathway and there is a stable equilibrium between the production of Aβ peptides and their clearance from the extracellular space **(Evin and Weidemann 2002, Bandyopadhyay, Goldstein et al. 2007)**.


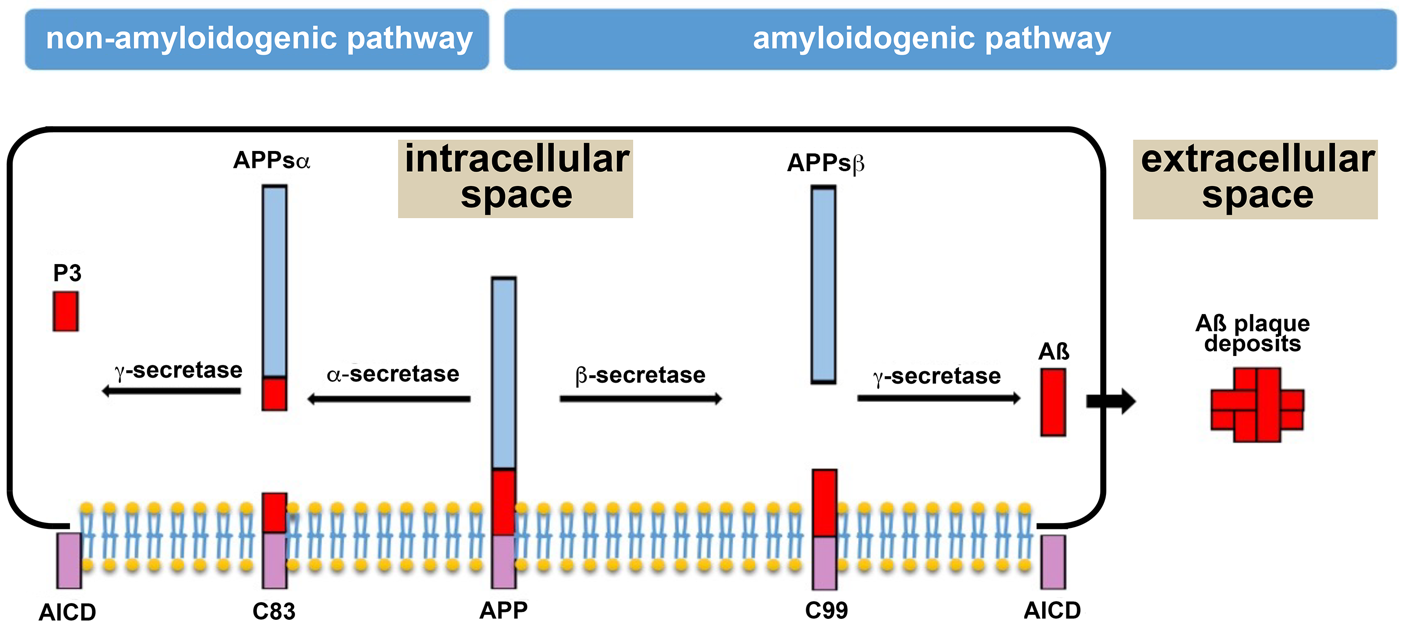


**Figure 2.** **Proteolytic cleavage of the amyloid precursor protein (APP) *via* the non-amyloidogenic and the amyloidogenic pathways.** Left side: Non-amyloidogenic pathway. This pathway involves sequential proteolysis of the amyloid precursor protein (APP) by alpha-secretase and gamma-secretase, which results in the formation of the non-toxic and water-soluble fragments APPsα and P3. Since the cleavage site of gamma secretase is located in the membrane-spanning domain of APP (AICD) a truncated version of this domain is left in the plasma membrane. This pathway is considered a protective mechanism against amyloid plaque formation. Right side: Amyloidogenic pathway. In this pathway, the initial proteolysis of APP is catalysed by beta-secretase yielding the water-soluble APPsß cleavage fragment. Next, gamma secretase cleaves the C99 fragment, which is still anchored in the plasma membrane, within its membrane-spanning domain leaving a truncated version of AICD in the plasma membrane. Since gamma-secretase cleavage is not strictly regio-specific, several Aß-peptides can be formed. Unfortunately, all Aβ peptides have the potential to aggregate, which leads to the formation of Aß plaques. This image was modified from **(Sasmita 2019)**.

The *APOE* polymorphism impacts the efficiency of Aß plaque formation. In fact, APOE4 protein expression augments Aβ production by elevating the catalytic activity of the gamma-secretase **(Lane-Donovan and Herz 2017)**. However, it does not only speed up the formation of Aß40-42 peptides, it also forms mixed peptide oligomers. The full-length APOE protein has a molecular weight of about 34 kDa but it is cleaved in the brain yielding an APOE-18kDa cleavage peptide **(Mouchard, Boutonnet et al. 2019)**. This peptide is neurotoxic by itself **(Tolar, Keller et al. 1999)** but it also forms even more toxic mixed peptide oligomers with Aß fragments **(Manelli, Bulfinch et al. 2007)**. Such mixed peptide oligomers are formed in much lower quantities when corresponding cleavage peptides of the APOE2 and APOE3 proteins are present **(Mouchard, Boutonnet et al. 2019)** and this observation is consistent with the detrimental role of the *APOE4* allele. Employing transgenic mice expressing different human *APOE* alleles, it has been shown that APOE4-expressing animals show more efficient Aß aggregation and amyloidogenesis than mice expressing the APOE3 and APOE2 isoforms **(Huynh, Davis et al. 2017)**. Moreover, experiments with *APOE-*inducible mouse models confirmed the observation that the APOE4 protein, but not the APOE3 protein accelerated A$\beta$ aggregation and thus, the formation of amyloid plaques **(Liu, Zhao et al. 2017)**. Interestingly, mixed APOE4-Aβ peptide complexes occur in brain tissue. However, they are also present in the cerebral vasculature impairing the perfusion of affected brain regions. Here again, APOE4-Aß complexes were more abundantly formed than APOE2-Aβ or APOE3-Aβ complexes **(Martel, Mackic et al. 1997, Kim, Basak et al. 2009)**.

**2.4. The role of APOE4 isoproteins for the clearance of Aß aggregates**

The steady-state tissue concentrations of Aß in the brain are regulated not only by the extent of A$\beta$ formation (influx control) but also by its proteolytic clearance (efflux control). Although the efflux mechanisms have not been studied in detail several proteins including APOE isoforms have been implicated in the clearing process **(Castellano, Kim et al. 2011)**. Under pathological conditions, there is a shift of the alpha-secretase pathway (non-amyloidogenic) to the beta- gamma-secretase (amyloidogenic) pathway, which leads to the accumulation of Aβ within the extracellular space in the brain. In AD patients, the total levels of Aβ_42_ monomers in the cerebro-spinal fluid decrease, but concentrations of oligomeric Aβ levels increase **(Tai, Bilousova et al. 2013)**. Various studies indicated that the APOE4 protein speeds up Aβ aggregation but simultaneously attenuates Aβ clearance **(Safieh, Korczyn et al. 2019)**.

The lipidation of the APOE protein in the central nervous system is an important process. Astrocytes synthesise the different APOE protein variants and release them into the extracellular space. Cholesterol and other lipids are also formed by these cells and are released *via* the ATP-binding assette A1 (ABCA1) transporter. Extracellularly, lipid-laden APOE nanoparticles are formed **(Hauser, Narayanaswami et al. 2011)**. These nanoparticles bind soluble A$\beta$ peptides and form insoluble APOE-A$\beta$ complexes. These lipidated complexes can be cleared from the extracellular space *via* receptor-mediated endocytosis by neurons and/or microglia cells and three different cell surface receptors have been implicated in the clearing process: i) low-density lipoprotein receptor (LDLR), ii) low-density lipoprotein receptor-related protein-1 (LRP1) and iii) heparin sulphate proteoglycan (HSPG). The efficiency of the clearing process depends on the composition of the APOE-Aß complexes and APOE2-containing complexes are most effectively cleared (APOE2 > APOE3 >> APOE4). In contrast, APOE4 containing complexes are rather resistant to clearence and will accumulate. A$\beta$ oligomers and mixed APOE-Aß complexes can cross the blood-brain-barrier (BBB) *via* LRP1-dependent pathways to enter the blood stream. There, they are degraded by circulating proteases, such as insulin-degrading enzyme (IDE) or angiotensin-converting enzyme (ACE), but
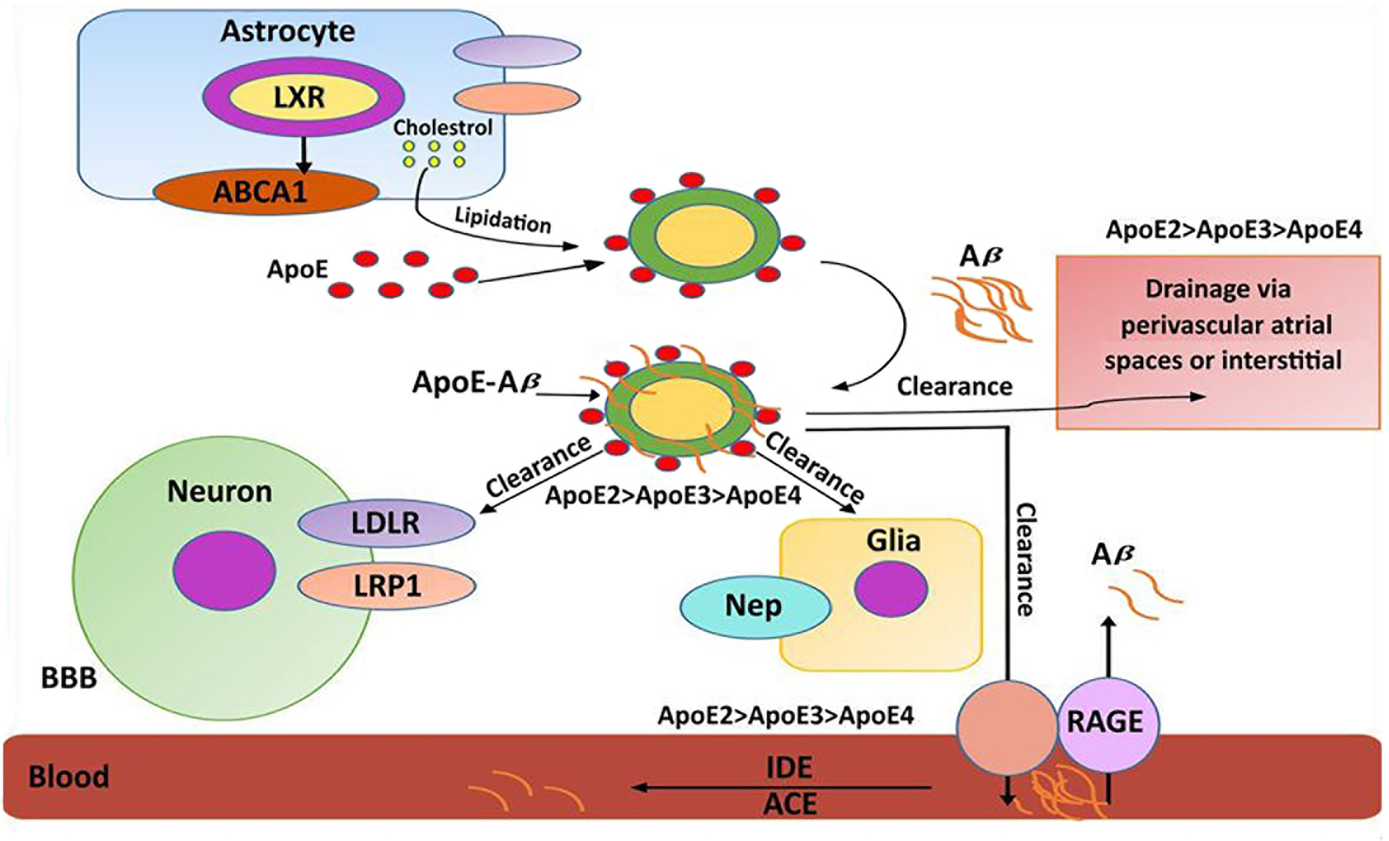
also by more unspecific proteases, such as neprilysin (Nep) **(Kim, Basak et al. 2009, Dries, Yu et al. 2012)**.

**Figure 3. Clearance of Aβ peptides from the brain.** There are three major pathways by which A$\beta$ is cleared from the extracellular compartment of the brain: i) extracellular proteolytic degradation; ii) Cellular uptake by neurons and glia cells and subsequent intracellular lysosomal degradation; iii) transfer from the extracellular space of the brain into the blood and proteolytic cleavage by circulating blood proteases. Abbreviations: LDLR, low-density lipoprotein receptor; LRP1, low-density lipoprotein receptor-related protein; HSPG, heparin sulphate proteoglycan; BBB, blood-brain barrier; ABCA1, ATP-binding cassette A1 transporter; LXRs, liver X receptors; IDE, insulin-degrading enzyme; ACE, angiotensin-converting enzyme; Nep, neprilysin; RAGE, receptor for advanced glycosylation end products. This image was modified from **(Yoon and Jo 2012)**.

Neprilysin, which has been implicated in cerebral clearance of A$\beta$ peptides **(Pacheco-Quinto, Herdt et al. 2013, Hüttenrauch, Baches et al. 2015)**, is a zinc-containing unspecific metalloprotease that is expressed in many cells and issues (kidney, lungs, adipose tissue, bone immune cells, testis and others). In the brain, nephrilysin expression occurs less abundantly when compared with the kidney and lungs, but it has been detected in GABA-ergic and metabotrophic hlutamate 2/3 receptor-positive neurons **(Fukami, Watanabe et al. 2002)**. In contrast, cholinergic neurons are apparently free of nephilysin **(Fukami, Watanabe et al. 2002)**. The nephrilysin protein is encoded by the *MME* (membrane metallo endopeptidase) gene, which is located in the central region of the long arm of chromosome 3. The enzyme cleaves a number of different biologically relevant peptides including bradykinin, angiotensin II, substance and enkephalins. It also hydrolyzes A$\beta$ peptides. *MME*^-/-^ mice develop AD-like symptoms and subtle Aß deposits in the brain. Thus, neprilysin is an important ß-amyloid-degrading enzyme **(Hafez, Huang et al. 2011)**.

RAGE, which is also called AGER (advanced glycation end product receptor), is a cell surface receptor that interacts with A$\beta$ peptides and has been implicated in the clearance of A$\beta$ complexes from the brain. Although RAGE facilitates the transport of A$\beta$ across the BBB (beneficial process), it may also import Aß peptides from the circulation into the brain and thus, may incduce neuro-inflammation. Since A$\beta$ import is clearly detrimental for the brain, RAGE may play a dual role in the pathogenesis of AD **(Yan, Chen et al. 2012)**. The processes involved in the clearing of Aß peptides are summarised in **Figure 3**.

**2.5. *APOE4* and tau protein pathology**

The second major morphological hallmark of AD are neurofibrillary tangles **(Ando, Laborde et al. 2014).** These tangles (NFTs) constitute fibrous protein structures formed within the cellular body of neurons but also in the dendritic spines. They consist of hyperphosphorylated isoforms of the tau protein, which aggregate intracellularly and thus, impair neuronal function **(Ando, Laborde et al. 2014, Reitz and Mayeux 2014)**. In epidemiological studies, a statistical correlation was found between the presence of the *APOE4* allele and the concentration of tau proteins in the cerebrospinal fluid. Interestingly, this correlation showed a higher degree of statistical significance in females than in males **(Hohman, Dumitrescu et al. 2018)**. Moreover, NFT formation appears to correlate more closely with the cognitive decline of AD patients than with the formation of amyloid plaques. Thus, NFT formation may be considered a more reliable diagnostic parameter to judge the clinical severity of AD than amyloid plaque formation **(Giannakopoulos, Herrmann et al. 2003)**.

The tau-proteins (tubulin-associated units) represent a family of water-soluble protein isoforms, which are encoded by the *MAPT* (microtubule-associated protein tau) gene. In humans, this gene is located in a central region (q21.31) of the long arm of chromosome 17 and is expressed in a large number of mammalian cells including neurons **(Neve, Harris et al. 1986, Goedert, Wischik et al. 1988)**. In other cells of the CNS (astrocytes, oligodendrocytes), the *MAPT* gene is only expressed at low levels. Tau proteins have been implicated as regulatory proteins in the formation of microtubules and play important roles in the structure and function of the cytoskeleton. In neurodegenerative diseases including AD **(Morris, Maeda et al. 2011)** the water-soluble tau proteins are frequently hyperphosphorylated and form insoluble aggregates (neurofibrillary tangles). These tangles are neurotoxic and impair neuronal functions **(Hampel, Blennow et al. 2010)**.

Under physiological conditions, tau proteins usually do not occur as free cytosolic proteins but they associate with various binding partners such as microtubules, Src and APOE. However, when phosphorylated the tau proteins are released from their binding partners and lose their specific functions **(Bhaskar, Yen et al. 2005, Morris, Maeda et al. 2011)**. In transgenic mice overexpressing APOE4 in neurons tau hyperphosphorylation was observed. This observations suggested that APOE4 may play a key role in the progression of neuronal defects related to AD **(Brecht, Harris et al. 2004)**. Furthermore, neuronal proteolysis of APOE4 leads to the generation of truncated APOE fragments which trigger tau phosphorylation **(Huang, Liu et al. 2001, Harris, Brecht et al. 2003)**. In APOE4 transgenic mice tau hyperphosphorylation was associated with activation of the extracellular signal-regulated kinase (ERK) and zinc ions apparently play a role in this process **(Harris, Brecht et al. 2004)**.

In other mouse models the potential role of apoE isoproteins in tau phosphorylation is controversial. When the degree of tau-phosphorylation was quantified in apoE^-/-^ mice hyperphosphorylation was observed and this data suggested that expression of apoE might down-regulate tau phosphorylation **(Genis, Gordon et al. 1995)**. However, in a follow-up study this observation could not be confirmed since the patterns of tau phosphorylation were similar when wildtype mice were compared with apoE^-/-^ animals. Here the autors concluded that the lack of expression of the apoE proteins may not interfere with expression, distribution and phosphorylation of tau proteins **(Mercken and Brion 1995)**. The possible reasons for these controversial data remain unclear but it might be related to background problems of the employed apoE^-/-^ mice, to specificity problems of the employed antibodies or to other technical reasons. When Aß oligomers were injected into the lateral ventricles of apoE^-/-^ mice and of corresponding wildtype controls it was found that the degree of tau-phosphorylation was higher in wildtype mice. Moreover, in vitro studies indicated APOE4 treated apoE^-/-^ neurons exhibited more phosphorylated tau proteins than APOE3- and APOE2-treated neurons. Taken together, these results suggest that APOE may facilitate tau phosphorylation in an isoform-specific way **(Hou, Han et al. 2020)**. However, the molecular basis for this observation has not been explored in this study.

To address this point it was explored whether APOE treatment of neurons affected the catalytic activity of enzymes that have previously been implicated in tau phosphorylation, such as glycogen synthase kinase 3beta (GSK3ß), P35 and cyclin-dependent protein kinase 5 (CDK5). Treatment of primary neurons with APOE (2 µM) attenuated the cellular concentrations of phospho-GSK3ß, P35 and CDK5 and also decreased the levels of phosphorylated tau. The alteration of tau phosphorylation was blocked by an inhibitor of the low-density lipoprotein receptor family suggesting that the observed effects were due to a specific receptor-ligand interactions. From these data the authors conclude that APOE isoproteins might modulate tau phosphorylation via several receptor-dependent phosphorylation pathway **(Hoe, Freeman et al. 2006)**. When the experiments were repeated at 100 nM APOE concentrations tau phosphorylation was not affected. Since the APOE concentrations used in these experiments were rather high (2 µM) it remains to be shown whether these in vitro data are of any in vivo relevance.

**2.6 APOE4 and gene expression regulation**

All nucleated human cells involve the same nuclear genome, but only a subset of these genes is expressed at a given time point in a given cell type. The decision, which gene is expressed at a certain time point is made by a complex regular network that is generally referred to as gene expression regulation **(Pope and Medzhitov 2018)**. In these regulatory networks expression of a certain gene product modifies the expression of other genes. In other words, expression of the APOE4 protein instead of the other APOE-isoforms alters the expression of other genes. Although the heritability of AD is high, knowledge of the disease-associated genes and their expression regulation still remains limited. However, recent studies have revealed that changes in gene expression patterns of AD related genes strongly impact neuronal function and neurodegeneration. In fact, activation and inactivation of transcription **(Jiang, Zhang et al. 2013, Yang, Zhang et al. 2023)** and translation factors **(Oliveira and Klann 2022)**, expression of non-coding RNA species **(Zhou, Zhao et al. 2019)**, alternative splicing **(Biamonti, Amato et al. 2021, Farhadieh and Ghaedi 2023)** and epigenetic mechanisms **(Sharma, Mehta et al. 2020)** may play a role in the pathogenesis of AD **(Bagyinszky, Giau et al. 2020)**.

Transgenic mice expressing human APOE3 or APOE4 isoforms show markedly distinct gene expression patterns in different brain regions **(Lattanzio, Carboni et al. 2014)**. For instance, the steady-state mRNA concentrations encoding for the peptidyl-prolyl cis/trans isomerase (Pin1) were significantly higher in the hippocampus of APOE4 expressing mice than in the corresponding regions of APOE3 expressing control animals. Contrast, in the cortex of the entorhinal and parietal regions, lower expression levels were detected. Sirt1 levels were significantly reduced in the frontal cortex of APOE4 mice and these alterations may play a role in APOE4-associated memory impairments. Moreover, in APOE4 mice, presenilin (PS) mRNA levels were reduced in the frontal cortex, which might affect APP processing. In contrast, cellular levels of the brain-derived neurotrophic factor (BDNF) did not differ between APOE3 and APOE4 mice in any analysed brain region **(Lattanzio, Carboni et al. 2014)**. Taken together, these data show that transgenic expression of the APOE4 protein dysregulated the expression characteristics of the *Pin1*, *Sirt1* and *PS1* genes in different brain areas and the observed differences might contribute to the increased AD vulnerability of APOE4 mice. However, it remains to be explored in the future whether or not similar expression regulation characteristics also occur in the brains of human AD patients.

Impairment of adult neurogenesis is part of AD in humans and this patho-physiological aspect involves altered neuronal gene expression patterns. *Post-mortem* transcriptome analyses of human AD brains indicated an upregulation in the expression of neural progenitor and proliferation markers together with an anti-prallel downregulation in the expression of later neurogenic markers **(Gatt, Lee et al. 2019)**. Thus, the overall level of human adult neurogenesis is reduced during the later stages of AD which may be related to compromised maturation and integration of “new-born” neurons. However, the exact molecular mechanisms underlying these differences between normal and compromised neurogenesis remain to be explored in the future.

**2.7. APOE4 and lipid metabolism**

**2.7.1. APOE4 in cholesterol, PUFAs and lipid-driven energy metabolism**

APOE is an anti-atherogenic apolipoprotein **(Greenow, Pearce et al. 2005)**. In contrast to wild-type mice, *apoE*-deficient mice (*Apoe*^-/-^ mice) are athero-susceptible and these animals are frequently used to explore the mechanistic basis of human atherogenesis **(Ma, Wang et al. 2017).** APOE is expressed in various tissues tissues, including liver, lung, brain, spleen, ovary, kidney, and adrenal gland **(Mahley 1988)** and ts canonical function is to contribute to HDL-mediated reverse cholesterol transport. *Via* this mechanism, it is responsible for the removal of excessive cholesterol from the plasma membranes of peripheral cells. After cholesterol loading, APOE is transported to the liver, where the cholesterol is converted to bile acids and excreted with the faces **(Mahley 1988)**. However, more than 80% of the bile acids secreted into the gut are subsequently re- absorbed into the blood (entero-hepatic circulation) **(Roberts, Magnusson et al. 2002)**. In other words, enteral excretion of cholesterol is limited.

Alimentary intake of omega-3 polyunsaturated fatty acids, such as eicosapentaenoic acid (EPA) and docosahexaenoic acid (DHA), has been associated with a reduced risk for the development of LOAD **(Troesch, Eggersdorfer et al. 2020, Power, Nolan et al. 2022)**. However, this link may not be functional in *APOE4* allele carriers. This conclusion was based on the finding that *APOE4* allele carriers beta-oxidise more omega-3 polyenoic fatty acids than *APOE2* or *APOE3* carriers. Consequently, *APOE4* allele carriers have fewer omega-3 polyenoic fatty acids available for introduction into the membrane lipids, which may reduce the fluidity of the neuron plasma membrane, or for the formation of eicosanoids (see 2.6.2.) and/or endocannabinoids (see 2.6.3.).

Mitochondria-associated membranes (MAM) represent a region of the [endoplasmic reticulum](https://en.wikipedia.org/wiki/Endoplasmic_reticulum) (ER) that is reversibly connected with [mitochondria](https://en.wikipedia.org/wiki/Mitochondria). These membranes have been implicated in the import of lipids synthesised in the ER into the mitochondria and thus, they are essential for regular mitochondrial function **(Vance 2014)**. When astrocytes were treated with astrocyte-conditioned medium containing APOE4 communication of the ER with mitochondria was significantly improved when compared with APOE3-containing astrocyte-conditioned medium **(Tambini, Pera et al. 2016)**. These data are consistent with the assumption that the pathogenesis of AD involves an upregulation of MAM functionality. However, how exactly an upregulated mitochondrial function may contribute to the pathogenesis of AD remains to be explored in the future.

Dysregulated lipid metabolism of glia cells leads to the formation of lipid droplets and these transient lipid storage organelles are considered early biomarkers of neurodegeneration **(Liu, Zhang et al. 2015)**. ApoE deficiency in mice induces hyperlipidemia, which leads to lipid deposition in the wall of the arteries **(Jofre‐Monseny, Minihane et al. 2008, Maeda 2011)**. Whether apoE deficiency also impacts the lipid metabolism of the brain is currently a major topic of diccussion, but more detailed studies are required to understand the exact mechanism of APOE4 function in neuronal lipid metabolism **(Liu, MacKenzie et al. 2017)**.

**2.7.2. Eicosanoids and APOE4 in the pathogenesis of AD**

Eicosanoids and related compounds are lipid mediators, which are synthesised from arachidonic acid and other omega-3 and omega-6 PUFAs **(Simopoulos 2002)**. As pleiotropic signalling molecules **(Dimitrow 2009)** they have been implicated in the regulation of neuronal function and thus, may play a role in the pathogenesis of AD. Eicosanoids are biosynthesized via three different metabolic pathways **(Biringer 2019)**:

i) Prostaglandin G synthase (PTGS) pathway: The major end products of this metabolic pathway, which is more commonly known as the cyclooxygenase (COX) pathway, are prostaglandins, thromboxanes and prostacyclins. These oxidised PUFA derivatives represent short-living endocrine, paracrine or autocrine metabolites, which mainly exhibit their biological functions *via* binding to G-protein-coupled cell surface receptors. The key enzyme of this metabolic route is PTGS (COX), which is expressed in two different isoforms. PTGS-1 (COX-1) is constitutively expressed in many different cell types and is responsible for the formation of prostaglandins, regulating a large number of physiological processes such as gastroprotection **(Langenbach, Morham et al. 1995, Kulmacz 1998)**, urine production **(Dubois, Abramson et al. 1998)**, thrombocyte aggregation **(Yu, Cheng et al. 2005)** and pain perception **(Fiorucci, Antonelli et al. 2001)**. In contrast, PTGS-2 (COX-2) is mainly expressed in inflammatory cells and plays an important role in the biosynthesis of pro-inflammatory mediators **(Funk 2001)**. Selective PTGS-2 inhibitors (COXIBS) are frequently prescribed as anti-inflammatory drugs **(Benelli, Venè et al. 2018)**. Although some COXIBS have adverse cardio-vascular side effects **(Cairns 2007)** they are frequently prescribed as anti-inflammatory drugs. The two PTGS-isoforms are encoded by two different homologous genes, which are located in a distal region of the long arm of chromosome 9 (PTGS-1) and in a central region of the long arm of chromosome 1 (PTGS-2).

ii) Arachidonic acid lipoxygenase (ALOX) pathway : The human genome involves six functional *ALOX* genes (*ALOX5, ALOX15, ALOX15B, ALOX12, ALOX12B* and *ALOXE3*). Except for the *ALOX5* gene, which is located in the centromere region of chromosome 10, all other *ALOX* genes have been mapped to a joint *ALOX* gene cluster on the short arm of chromosome 17. The encoded enzymes catalyse the dioxygenation of free and esterified PUFAs to the corresponding hydroperoxy compounds. *In vivo*, these lipid hydroperoxides are rapidly reduced by peroxidases to the corresponding alcohols and thus, hydroxylated PUFAs are the first stable reaction product. When these hydroxy PUFAs are present in large quantities in the lipid bilayer of biomembranes, the membrane structure is disturbed and the cells or subcellular organelles may fall apart **(Biringer 2019)**. Although the six different human ALOX-isoforms share a high degree of structural similarity, their functional characteristics are very different and single knockout studies of mouse *Alox* genes suggested different biological functions for the different isoenzymes **(Funk, Keeney et al. 1996, Epp, Fürstenberger et al. 2007, Hallenborg, Jørgensen et al. 2010, Hallenborg, Jørgensen et al. 2010)**. In other words, the coding multiplicity of the ALOX-isoforms is not an indication of functional redundancy. ALOX-isoforms are also involved in the biosynthesis of anti-inflammatory and pro-resolving mediators such as lipoxins **(Spite, Norling et al. 2009, Schebb, Kühn et al. 2022)**, resolvins **(Tjonahen, Oh et al. 2006)** and maresins **(Schäfer, Reisch et al. 2023)**. Although the biological role of these ALOX metabolites has recently been challenged **(Schebb, Kühn et al. 2022, O'Donnell, Schebb et al. 2023)** they might play a regulatory role in neuroinflammation **(Yao, Clark et al. 2005, Yang, Zhuo et al. 2010)** and thus, in the pathogenesis of AD. Among the different ALOX isoforms, ALOX5 and ALOX15 have been suggested to be of particular relevance **(Czapski, Czubowicz et al. 2016)**. The intracellular catalytic activity of ALOX-isoforms depends on the cellular redox state and expression of the ALOX5 and ALOX15 genes is inversely regulated by cytokines **(Spanbroek, Hildner et al. 2001)**.

iii) Cytochrome P450 pathway: Cytochrome P450 isoforms are oxygen-activating enzymes and most of them function as monooxygenases **(Hrycay and Bandiera 2012)**. For eicosanoid biosynthesis, they mainly catalyze epoxygenation of unsaturated fatty acids **(Capdevila, Falck et al. 2000)**. These fatty acid epoxides are rapidly hydrolyzed, which leads to the formation of vicinolic dihydroxylated PUFAs. Alternatively, cytochrome P450 enzymes may also catalyse omega- and/or omega-1 oxidation of fatty acid, forming monohydroxylated fatty acid derivatives.

Eicosanoid biosynthesis *via* these three pathways has been implicated in the pathogenesis of AD, but the underlying molecular mechanisms remain largely elusive. Some eicosanoids exacerbate AD pathology, while others exhibit beneficial effects **(Biringer 2019)**. Unfortunately, for the time being, there is no unifying concept that proves the principle importance of either of these mediators or of their biosynthesizing enzymes for the pathogenesis of AD. A number of clinical trials have been carried out in AD patients using specific PTGS-2 inhibitors (COXIBS), but all the results obtained so far were negative **(Firuzi and Praticò 2006)**. Except for the isoform-specific ALOX5 inhibitor zileuton **(Carter, Young et al. 1991)**, which has been approved for clinical use as an anti-asthmatic drug in the US, there is no other clinically approved ALOX inhibitor available at the moment. Thus, for the time being, it is impossible to perform clinical studies testing the potential effects of isoform-specific ALOX inhibitors in AD patients. However, a number of different knock-out **(Martens, Novotny et al. 2002), (Dobrian, Lieb et al. 2010)**, knock-in **(Epp, Fürstenberger et al. 2007, Hallenborg, Jørgensen et al. 2010) (Marbach-Breitrück, Rohwer et al. 2021)** and transgenic mice are currently available for both, the PTGS- and ALOX-pathway. Unfortunately, most of these animals have not been tested in mouse models of AD. The lack of such *in vivo* experimental data may also be related to the fact that the currently available mouse models of AD are rather complex and do not mirror all major aspects of human AD **(Yokoyama, Kobayashi et al. 2022)**. Unfortunately, the eicosanoid metabolism of APOE2, APOE3 and APOE4 transgenic mice has not been studies in detail and thus, it remains unclear how overexpression of these human alleles impact systemic eicosanoid biosynthesis.

**2.7.3. Cannabinoids, endocannabinoids and APOE4 in the pathogenesis of AD**

Cannabinoids are exogenous chemical substances that bind with high affinity to the major cannabinoid receptors (CB1, CB2) of the human body. They induce similar effects as tetrahydrocannabinol (THC), which is synthesised in large quantities by *Cannabis sativa* **(Abyadeh, Gupta et al. 2021)**. Although this plant produces up to 100 chemically distinct cannabinoids, the two major compounds are THC and cannabidiol (CBD), which are responsible for the psychogenic effects of cannabis **(Hazekamp, Fischedick et al. 2010)**. Since THC induces strong psychoactive effects **(Bhattacharyya, Fusar-Poli et al. 2009)** whereas CBD is mainly anti-psychoactive, the mass ratio of these two chemicals is decisive for the psychogenic effect of a cannabis plant. CBD also reduces some other negative effects of THC such as anxiety **(Crippa, Zuardi et al. 2004)**. Endocannobinoids are endogenous substances in the human body that also bind to CB1 and/or CB2 and induce similar psychogenic effects as THC. In the human body, two major endocannabinoids [anandamide (AEA) and 2-arachidonylglycerols (2-AG)] have been identified, and both of them carry an arachidonic acid residue. They have been implicated in neuronal function but also play a role in the regulation of other physiological processes such as [pregnancy](https://en.wikipedia.org/wiki/Pregnancy), cell development, the immuneresponse, apetide, [pain sensation](https://en.wikipedia.org/wiki/Nociception), [mood](https://en.wikipedia.org/wiki/Mood_(psychology)) and memory. In the central nervous system, endocannabinoids play important roles in the control of movement and motor coordination, in learning and memory, and in emotion and motivation. Moreover, as endorphins, endocannabinoids function as endogenous analgesics **(Kaur, Ambwani et al. 2016)**.

On the molecular level, endocannabinoids function as retrograde neurotransmitters. These compounds are synthesized by presynaptic neurons and subsequently released into the synaptic space. There, they bind to the CB receptors localised in the pre-synaptic membrane and down-regulate the further release of other neurotransmitters. In other words, they prevent neuronal hyperactivity. Since the endocannabinoid system is highly expressed in the hippocampus and in the cortex, endocannabinoids have been associated with learning and memory. These cerebral functions are frequently defective in AD patients, and it has been suggested that AD patients carry a compromised endocannabinoid system **(Basavarajappa, Shivakumar et al. 2017)**. Moreover, recent findings in AD rodent disease models have shown that cannabinoids are capable of reducing amyloid plaque formation and stimulate hippocampal neurogenesis . Beneficial effects of cannabinoids on other dementia-related symptoms have also been reported in clinical trials. Accordingly, future studies should be focused on optimising the therapeutic dosages and the time protocol for the use of cannabinoids as drugs in AD therapy **(Coles, Steiner-Lim et al. 2022)**.

Endocannabinoids are typically broken down by unspecific hydrolases, which include fatty acid amide hydrolase and monoacylglycerol lipase. However, they also serve as substrates for cyclooxygenases **(Urquhart, Nicolaou et al. 2015)**, cytochrome P450-isoforms and lipoxygenases **(Ivanov, Kakularam et al. 2021)**. As indicated above (2.5.2.), these enzymes oxygenate the arachidonic acid backbone, which leads to the formation of an entirely novel array of bioactive lipids **(Schwitter, Lutz et al. 2023)**. The bioactivities of these oxygenated endocannabinoids are different from those of the non-oxygenated parent compounds, and thus, enzymatic oxygenation modifies the functionality of the endocannabinoid system, which contributes to the pathogenesis of AD **(Abate, Uberti et al. 2021)**.

As for eicosanoid biosynthesis, the endocannabinoid metabolism of APOE2, APOE3 and APOE4 transgenic mice has not been studied and thus, it remains unclear how overexpression of these AD related gene products impact systemic endocannabinoid turnover.

**2.7.4. Sphingolipids and APOE4 in the pathogenesis of AD.**

Sphingolipids form a class of biologically active lipids that are important constituents of biomembranes **(Loewith, Riezman et al. 2019)** but also play a role as lipid signalling molecules **(Kleuser 2018)**. They have been implicated in the pathogenesis of AD **(Czubowicz, Jęśko et al. 2019)** but the patho-physiological mechanisms have not been explored in detail **(Hannun and Obeid 2018).** Disruption of sphingolipid metabolism can result in the accumulation of specific sphingolipid species including ceramides and sphingosine-1-phosphate (S1P), which have been implicated in AD **(Han, Rozen et al. 2011)**. Ceramides have been linked to increased Aß levels **(Dehghan, Pinto et al. 2022)**. Sphingolipids have also been implicated in the immune response and in neuroinflammation, which are prominent aspects of AD **(Maceyka and Spiegel 2014)**. S1P upregulates activation and migration of cerebral microglia and thus contributes to chronic cerebral inflammation **(Qi, Heng et al. 2022)**, which may exacerbate neurodegeneration in AD **(Maceyka and Spiegel 2014)**.

As indicated above, sphingolipids are functionally relevant constituents of the plasma membranes of all mammalian cells including neurons. Since these lipids are usually devoid of polyunsaturated fatty acids, they reduce membrane fluidity **(McGonigal, Barrie et al. 2019)**. An increase in the sphingolipid content of a membrane reduces membrane fluidity making the membrane less flexible. In neurons, this may lead to neuronal dysfunction, which is a common feature of AD pathogenesis **(Piccinini, Scandroglio et al. 2010)**. Moreover, recent studies suggest that sphingolipids might directly impact tau pathology. Specific ceramides have been associated with tau hyperphosphorylation, which is a key step in the formation of NFTs **(Randez-Gil, Bojunga et al. 2020)**. Sphingolipids are integral components of lipid rafts in cell membranes. Lipid rafts are known to be involved in the processing and accumulation of Aβ **(Taylor and Hooper 2007)**. Disruptions in lipid rafts due to changes in sphingolipid composition could potentially lead to increased Aβ production and aggregation **(Pham and Cheng 2022)**.

Recognising the involvement of sphingolipids in the pathogenesis of AD could have preventive and therapeutic implications. Targeting sphingolipid metabolism and their downstream signalling pathways may offer new approaches to slow down or prevent the progression of AD **(Hannun and Obeid 2018)**. However, the potential role of sphingolipids in the pathogenesis of AD is complex and evolves in a large number of areas of research. Understanding how these lipid molecules interact with other key factors in AD, such as Aβ, tau, and neuroinflammation, is essential for advancing our knowledge of the disease and developing effective treatments in the future.

**2.8. APOE4 and neuroinflammation in the pathogenesis of AD**

Neuroinflammation is one of the major processes in the pathogenesis of AD **(Lecca, Jung et al. 2022)** and APOE has been characterised as an inducer of neuroinflammation. APOE4 plays a major role in the induction of the innate immune response in the brain of AD patients, but the molecular mechanisms are apparently very complex **(Ophir, Amariglio et al. 2005, Cash, Kuhel et al. 2012, Dorey, Chang et al. 2014, Du, Jia et al. 2015)**. One of the major pro-inflammatory effects of APOE4 is activation of microglia, which subsequently induces neuroinflammation **(Rodriguez, Tai et al. 2014, Li, Montine et al. 2015)**. In this context, miRNA146a, which is present in the brain in large amounts, has been implicated in APOE4-induced neuroinflammation. In fact, AD patients have higher levels of miRNA146a, which leads to chronic inflammation and involves inadequate negative feedback regulation **(Lukiw, Zhao et al. 2008, Teter, LaDu et al. 2016)**. When neurons were incubated *in vitro* with different APOE isoforms, they secreted the classical pro-inflammatory cytokine interleukin-1ß and the extent of cytokine secretion was significantly higher when APOE4 was used instead of APOE3 **(Guo, LaDu et al. 2004, Dorey, Chang et al. 2014)**. When transgenic mice overexpressing alternatively either APOE4 or APOE3 were treated by intracerebroventricular injection of lipopolysaccharide (LPS) as inflammation inducer, expression patterns of inflammation-related gene products were similar, but the extent of pro-inflammatory gene expression was significantly elevated and more prolonged in APOE4 mice **(54)**. Detailed clustering analysis of the expression patterns indicated predominant expression of genes carrying NF-κB response elements. Direct quantification of the expression of NF-κB-regulated genes revealed that the extent of activation was more pronounced in APOE4 mice when compared with APOE3 animals. These findings suggested that the higher degree of neuroinflammation in APOE4 mice may be related to dysregulation of NF-κB signalling **(Ophir, Amariglio et al. 2005)**. In a medium-scale (some 2500 patients) epidemiological study, the impact of chronic low-grade peripheral inflammation in *APOE4* allele carriers was tested on the onset of AD **(Tao, Ang et al. 2018)**. Elevated plasma levels of C-reactive protein (CRP) shortened the latency for the onset of clinical AD symptoms, and the authors concluded that treating chronic systemic inflammation based on genetic risk might be considered an effective prevention method for premature development of AD symptoms.

In addition to APOE4, the triggering receptor expressed on myeloid cells 2 (TREM2) has been genetically linked to AD **(Golde, Streit et al. 2013).** Although the molecular basis for the genetic association has not been explored in detail, it was suggested that APOE4 may directly bind to TREM2 and thus alter TREM2 signaling. APOE isoforms function as TREM2 agonists with an EC_50_ in the low nM range, but the differences between the APOE isoforms were not particularly impressive **(Jendresen, Årskog et al. 2017)**. Although the binding was displaced by an APOE-mimetic peptide, the lack of isoform-specificity does not provide strong evidence for the involvement of these mechanisms in AD pathogenesis.

Neuroinflammation is a complex process and detrimental as well as beneficial neuroinflammatory phenotypes (NIP) have been reported **(Tai, Ghura et al. 2015)**. Although many studies have shown that APOE4 induces a detrimental phenotype of neuroinflammation, this overall effect might be related to a suppression of the beneficial mechanisms of neuroinflammation. APOE4 increases Aβ-induced pro-inflammatory receptor signalling (toll-like receptor 4-p38α), but it may also suppress beneficial receptor-mediated pathways (IL-4R nuclear receptor). Dysregulation in sphingolipid metabolism can lead to the buildup of distinct sphingolipid species, such as ceramides and sphingosine-1-phosphate (S1P), which have been associated with Alzheimer's disease (AD) .

**2.9. APOE4 and vascular integrity in the pathogenesis of AD**

In the brains of AD patients the functionality of the cerebral vasculature is dysregulated and *APOE4* carriers are particularly affected. APOE4 disrupts microvascular integrity, reduces cerebral blood flow, induces small vessel atherosclerosis and deteriorates amyloid angiopathy **(Mielke, Leoutsakos et al. 2011, Bell 2012, Tai, Thomas et al. 2016)**. Moreover, *APOE4* carriers have an increased risk for vascular dementia and generalised atherosclerosis **(Mahley, Weisgraber et al. 2009, Rohn 2014)**. From these observations, it can be concluded that AD patients might benefit from normalising the detrimental vascular effects induced by the *APOE4* allele **(Bell 2012)**. Although the molecular basis for the vascular alterations in the brains of AD patients is very complex, the accumulation of Aß in the cerebral arteries has frequently been reported **(Safieh, Korczyn et al. 2019)**. Vascular deposition of the Aß peptide does not only impair cerebral perfusion but also impairs the integrity of the blood-brain barrier **(Rannikmäe, Kalaria et al. 2014)**. It also induces activation of the coagulation cascade in the cerebral vasculature, leading to the formation of microthrombi **(Cortes-Canteli, Zamolodchikov et al. 2012)**.

**2.10. APOE4 and synaptic plasticity in the pathogenesis of AD**

Inter-neuronal communication is one of the major functional features of the human brain and this communication proceeds *via* synapses. The human brain involves some 10^14^ synapses and a single neuron carries between 1-200,000 of them. These communication organelles exchange information between neurons as well as between neurons and a[strocytes](https://en.wikipedia.org/wiki/Astrocyte) **(Perea, Navarrete et al. 2009)**. The activity of synapses is strengthened or weakened over time depending on the intensity of the flux of information and this regulatory process is called synaptic plasticity. Since memory and learning are represented by interconnected neuronal circuits, synaptic plasticity is one of the most important neurochemical bases for these higher brain functions. There are several mechanisms to improve synaptic plasticity including alterations in the quantity of neurotransmitter release **(Gaiarsa, Caillard et al. 2002)**. ALOX polymorphism impacts synaptic plasticity The regulation of axon or dendrite outgrowth, which is crucial for synaptic plasticity, is influenced by APOE isoforms. In general, APOE-containing lipoproteins protect neurons from apoptosis *via* low-density lipoprotein receptor-related protein-1 (LPRP1)-mediated pathways **(Hayashi, Campenot et al. 2007)**. However, APOE3 activates LRP1 signalling more effectively than APOE4 **(Sen, Alkon et al. 2012)**. Moreover, APOE isoforms show differential effects on neurite outgrowth. APOE3 activates dendrite outgrowth, whereas APOE4 inhibits this process **(Mahley, Nathan et al. 1996)**.

**3. Ferroptosis**

As apoptosis and necroptosis, ferroptosis is a regulated suicide pathway **(Galluzzi, Vitale et al. 2018)**, but mechanistically it must be separated from the other types of regulated cell death **(Yang and Stockwell 2016)**. It has been implicated in physiological killing of tumor cells but also in the pathogenesis of different dieseases with high socio-economic impact including neurodegeneration **(Stockwell 2022, Sun, Xia et al. 2022)**. Ferroptosis involves three major metabolic hallmarks, which do not play major roles in other cellular suicide mechanisms:

i) Excessive lipid peroxidation: Oxidation of membrane phospholipids carrying polyunsaturated fatty acids (PUFAs) leads to the formation of highly reactive lipid peroxides, which are either reduced to less reactive hydroxy lipids by glutathione peroxidases or undergo secondary decomposition reactions leading to the formation of free lipid radicals. These radicals can subsequently react with other membrane constituents and induce a cascade of secondary oxidation reactions that impair the barrier function of membranes, but also lead to functional impairment of membrane-bound receptors, enzymes, and ion channels. As secondary products of radical-mediated lipid peroxidation, reactive aldehydes, such as malondialdehyde (MDA) and hydroxynonenal (HNE), are formed. These compounds may further react with amino groups of proteins and nucleic acids to cause structural and functional modification **(Dixon, Lemberg et al. 2012)**.

ii) Impairment of lipid peroxide reduction: Under aerobic conditions, peroxidation of unsaturated lipids is a normal process, and excessive accumulation of lipid peroxides is usually prevented by hydroperoxy lipid reducing enzymes. Glutathione-dependent and glutathione-independent reduction mechanisms have been described and glutathione peroxidase 4 (GPX4) is one of the major players in controlling the cellular lipid peroxide tone. This enzyme reduces complex hydroperoxy lipids to less reactive alcohols at the expense of reduced glutathione (GSH) and/or alternative electron donors **(Dixon, Lemberg et al. 2012)**. When GSH is used as a reductant, glutathione disulfide (GS-SG) is formed that needs to be back-reduced *via* the GSH-reductase reaction, and this pathway requires sufficient amounts of reduced NADPH_2_, which mainly originates from the oxidative pentose shunt **(Saha, Stoll et al. 1992)**. A deficiency in catalytically active GPX4 impairs the cellular reductive capacity, which results in uncontrolled intracellular accumulation of lipid hydroperoxides **(Dixon and Stockwell 2019)**.

iii) Intracellular iron deposition: Biologically active transition metals such as iron, copper, cobalt, or manganese are capable of interacting with intracellular peroxides catalysing the homolytic breakdown of the hydroperoxy bond (RO-OH). This reaction (Fenton reaction) forms free radical intermediates (alkoxy radicals and hydroxy radicals), which can induce secondary oxidation reactions which might lead to the dysfunction of biomolecules. In ferroptosis accumulation of intracellular iron ions triggers an elevated production of free radicals, potentially surpassing the cellular reductive capacity. Under these conditions, the cellular redox state might irreversibly be disturbed, which drives the cells into ferroptotic cell death to avoid damage of surrounding cells and constituents of the extracellular matrix **(Dixon, Lemberg et al. 2012)**.

**3.1.** **Ferroptosis and Alzheimer's disease**

AD is a neurodegenerative disorder that is characterised by excessive iron accumulation in the brain **(Küpper, Levin et al. 2017)**. Such iron deposits are found in the extracellular space, where the iron interacts with Aβ-peptides **(Perry, Nunomura et al. 2002)** but also in the neurons. Until recently, cerebral iron deposition has mainly been discussed as inducer of oxidative stress **(Castellani, Moreira et al. 2007)**. This is still a valid interpretation, but in light of the recent findings that intracellular iron accumulation is a key process in ferroptotic signalling, alternative functional consequences of iron accumulation need to be considered. Neuronal ferroptosis has been suggested as a key process in the pathogenesis of AD, and the patho-physiological consequences of ferroptotic cell death in the development of AD have recently been reviewed **(Chen, Jiang et al. 2021, Jakaria, Belaidi et al. 2021, Wang, Wang et al. 2022, Zhang, Wang et al. 2022, Zhao, Yang et al. 2023)**. In these papers, the authors also discussed novel therapeutic concepts targeting AD-related cerebral iron deposition to prevent the functional defects associated with ferroptotic cell death. However, for the time being, the usefulness of these innovative therapeutic strategies is still a matter of discussion and clinical studies must be carried out to test their effectiveness.

Sirtuins are highly conserved NAD^+^-dependent enzymes that have been implicated in the pathogenesis of AD and other age-related diseases. In humans, there are 7 different sirtuin isoforms (SIRT1-7), which are localised either in the cytosol, in the mitochondria, and/or in the nucleus **(Lalla and Donmez 2013)**. Most of them exhibit deacetylase activities and regulate the expression and catalytic activity of a large number of gene products, which play important roles in metabolism, energy homeostasis and longevity **(Houtkooper, Pirinen et al. 2012)**. Overexpression of SIRT1 exhibits protective effects against the development of neurological symptoms in AD models, and thus, designing therapeutics based on SIRT1 activity might be useful to develop treatment methods for this disease **(Lalla and Donmez 2013)**. In fact, AD patients might benefit from the consumption of SIRT1 activators but should avoid the intake of SIRT1 inhibitors. Thus, understanding and modulating SIRT1 activity might be crucial in AD management **(Campagna, Spilman et al. 2018)**. In contrast to APOE3, APOE4 significantly reduces Sirt1 expression and modifies the ratio of neuroprotective Sirt1 to neurotoxic Sirt2. It also triggers phosphorylation of Tau and APP and induces programmed cell death **(Oliveira and Klann 2022)**. This data indicates that apoE4 impacts the delicate balance of sirtuin isoforms in the context of neuroprotection and opens innovative strategies for the treatment of AD. Moreover, APOE4 exhibits a higher binding affinity for Sirt1 when compared with APOE3 and APOE2 **(Lima, Hacke et al. 2020)**.

As indicated above, the anti-oxidative enzyme glutathione peroxidase 4 (GPX4) reduces hydroperoxy phospholipids and its deficiency induces ferroptosis **(Yang, SriRamaratnam et al. 2014).** However, a lack of GPX4 expression and/or inhibition of the catalytic activity of this enzyme is not the only mechanism involved in ferroptosis. In fact, dysregulated iron homeostasis is also of patho-physiological relevance **(Yang and Stockwell 2008, Gao, Monian et al. 2015)**. Together, intracellular iron accumulation and a lack of lipid peroxide-reducing capacity will induce oxidative stress and subsequent protein misfolding and aggregation **(Ashraf and So 2020)**.

Since the brain is one of the most lipid-rich organs of the human body and since brain cells are rich in PUFAs, the susceptibility of the brain to lipid peroxidation is particularly high. Peroxidation of PUFAs by transition metals **(Benedet and Shibamoto 2008)** or lipid peroxidizing enzymes induces oxidative stress, which is counteracted by antioxidant enzymes **(Savaskan, Borchert et al. 2007, Cardoso, Hare et al. 2017)**. In AD brains, lipid peroxidation products such as MDA, acrolein, F_2_-isoprostanes, and 4-hydroxynoneal (HNE) have been detected at elevated levels in parallel with Aβ-peptide plaques **(Vinothkumar, Kedharnath et al. 2017)**. In some studies, it has even been shown that Aβ-peptides promote lipid peroxidation, although the underlying mechanisms have not been identified **(Selley, Close et al. 2002)**. Moreover, HNE-protein adducts were detected in large quantities in the brains of AD patients **(Bruce-Keller, Li et al. 1998, Liu, Smith et al. 2005, Roberts, Ryan et al. 2012)**.

**3.2. APOE4 and ferroptosis**

Although the major function of APOE is its role in lipid transport **(Mahley, Innerarity et al. 1984)** it also impacts neuronal iron homeostasis **(Kagerer, van Bergen et al. 2020)**. APOE4 modifies the iron affinity of apoferritin, which is a major intracellular iron-binding protein **(Ayton, Faux et al. 2015)**. In cells, iron is essential for a number of basic processes. It functions as a cofactor for oxidoreductases, and thus, it is essential for the functionality of the respiratory chain. Moreover, iron is needed for the synthesis of neurotransmitters **(Singh, Haldar et al. 2014)**. Thus, dysregulation of neuronal iron homeostasis affect neuronal communication, which is needed for regular brain functionality **(Guo, Liu et al. 2018)**. Excessive neuronal iron loading impairs Aβ clearance *via* APOE-dependent mechanisms **(GOODMAN 1953, Van Bergen, Li et al. 2016)**. Furthermore, an iron response element (IRE) has been detected in the 5´-untranslated region of the human *APP* mRNA, and thus, expression of the APP protein appears to be up-regulated by iron on the translational level. In other words, increased intracellular iron concentrations stimulate the expression of APP mRNA and thus, may enhance Aβ formation **(Rogers and Lahiri 2004)**. In contrast, elevated iron concentrations downregulate the expression of the furin protein, responsible for activating β-secretase and subsequently initiating the amyloidogenic pathway in amyloid precursor protein (APP) metabolis **(Ward, Zucca et al. 2014)**. Excess of intracellular iron deposition can induce amyloidogenesis and Aβ generation *via* the interaction of the ferritin light chain with the presenilin enhancer-2 (PEN2), a component of the gamma-secretase **(Li, Liu et al. 2013)**. Ferritin itself can bind to Aβ peptides initiating Aβ fibril formation **(Balejcikova, Siposova et al. 2018)**. As described before, neurofibrillary tangles (NFT) are characteristic morphological substrates of AD. NFT deposits involve iron **(Smith, Harris et al. 1997)** and iron overload in neurons induces hyperphosphorylation of the tau protein **(Shin, Kruck et al. 2003)**.

Under normal conditions, the iron homeostasis of brain cells is well regulated. Fe^2+^ enters the brain by crossing the BBB via the divalent metal ion transporter 1 (DMT1). In brain cells, Fe^2+^ activates different types of protein kinases, such as glycogen synthase kinase-3β (GSK3β) and cyclin-dependent protein kinase 5 (CDK5). When activated, these protein kinases hyperphosphorylate tau proteins in a time-dependent manner, which initiates TNF formation **(Guo, Wang et al. 2013)**. The initial steps of tau-hyperphosphorylation are catalysed by CDK5, whereas GSK3β is responsible for the later phosphorylation steps. Thus, the concerted interaction of the two protein kinases leads to TNF formation **(Hanger, Anderton et al. 2009, Guo, Wang et al. 2013)**.


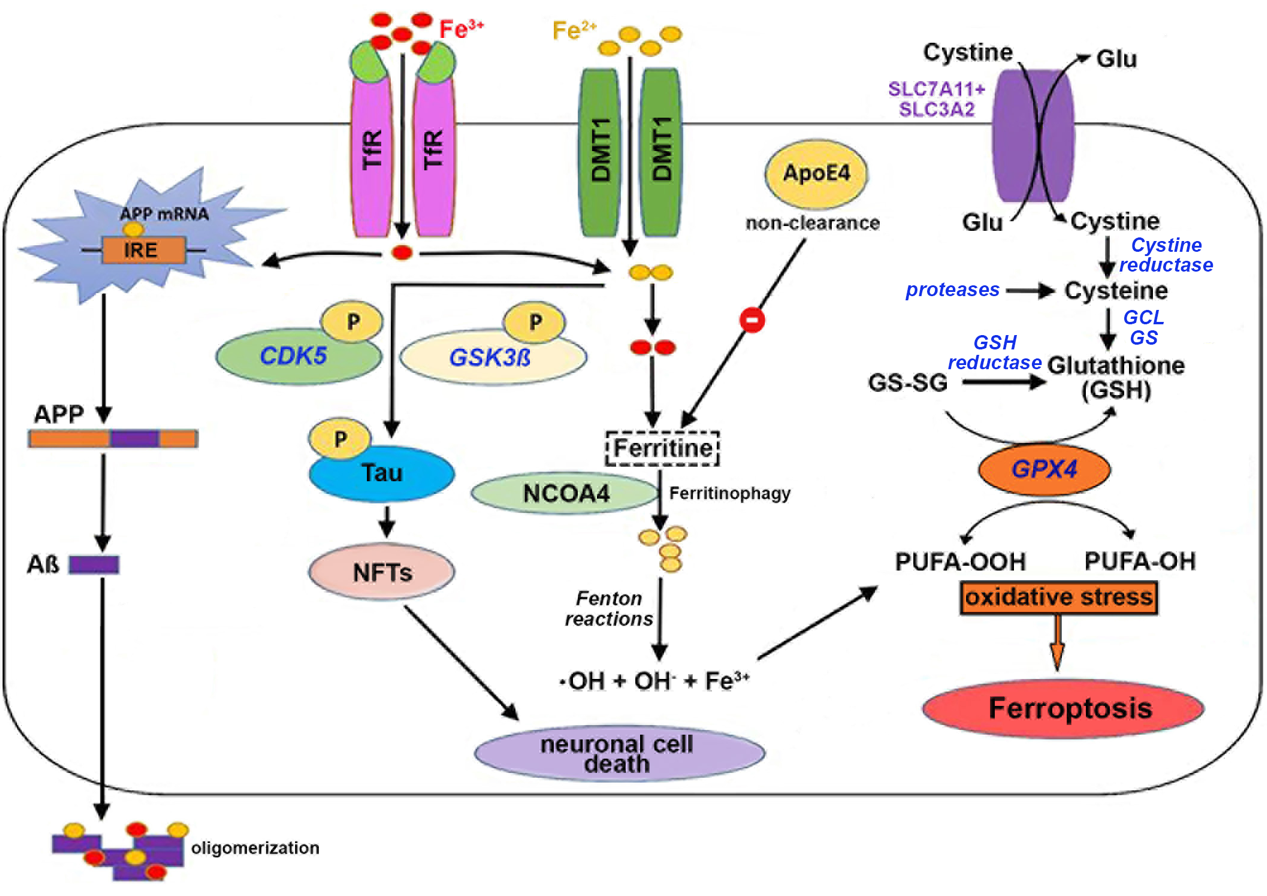


**Figure 4. The interplay of the APOE4 protein, lipid peroxidation and ferroptosis in the pathogenesis of Alzheimer's disease.** Iron ions cannot penetrate cellular membranes without the help of cell surface transporters. As in most other cells, neurons take up Fe^3+^ ions mainly *via* the transferrin receptor pathway (TfR). Alternatively, Fe^2+^ ions may enter neurons *via* the divalent metal ion transporter 1 (DMT1). Intracellularly, iron may function as ferrous (Fe^2+^) or ferric (Fe^3+^) ions. Ferrous iron may bind to the iron responsive element localised in the 5´-untranslated region of the APP mRNA, upregulating the expression of the APP gene and stimulating the formation of Aβ and its extracellular oligomerization. In other words, iron accumulation inside neurons upregulates amyloidogenesis. Furthermore, excessive presence of Fe^2+^ triggers phosphorylation of glycogen synthase kinase 3β (GSK3β) and cyclin-dependent protein kinase 5 (CDK5) and these reactions induce hyperphosphorylation of tau proteins and intracellular formation of neurofibrillary tangles (NTFs). NFTs trigger neuronal dysfunction and cell death. The redox-sensitive amino acid cysteine, which is an essential constituent of the glutatione (GSH) / glutathione disulfide (GS-SG) anti-oxidative defence system, enters neurons via the solute carrier 7A11 (SLC7A11) antiporter, which exchanges the amino acids glutamate (Glu) and cystine. Intracellularly, cystine is reduced to cysteine (Cys) by cystine reductase that uses NADH as electron donor. Cysteine may also originated from proteolytic breakdown of intracellular protein. Subsequently, Cys is used as substrate for glutathione (GSH) synthesis, which proceeds via two consecutive steps of amino acid transferase reaction. Both reactions are ATP-dependent and require gamma-glutamate-cysteine ligase (GCS) and glutathione synthase (GS) as catalyszing enzymes. Alternatively, GSH is formed from glutathione disulfide (GS-SG) via the glutathione reductase reaction, which employs NADPH as electron donor. Reduced glutathione (GSH) is the most frequently employed electron source for the GPX4 reaction, which reduces lipid hydroperoxides (PUFA-OOH) to the corresponding alcohols (PUFA-OH). When GSH is limiting hydroperoxy lipids accumulate and excessive iron deposition induces oxidative stress and ferroptosis. This image was modified from **(Sun, Xia et al. 2022)**.

APOE is a potent inhibitor of ferroptosis. It activates the PI3K/AKT pathway and inhibits autophagic degradation of ferritin and thus averts iron-dependent lipid peroxidation **(Belaidi, Masaldan et al. 2022)**. Quantifying the iron content in *post-mortem* samples of inferior temporal brain cortex, it was found that the association of iron with clinical symptoms of AD was stronger in patients carrying the *APOE4* allele. Since protection against ferroptosis did not differ between APOE isoforms in vitro, other features of APOE4 carriers, such as a low abundance of APOE protein expression and/or higher levels of PUFAs, could mediate the higher risk of *APOE4* allele carriers for AD **(Belaidi, Masaldan et al. 2022).**

APOE4 also increases the risk of vascular dementia and atherosclerosis **(Mahley, Weisgraber et al. 2009, Rohn 2014)** and thus, cerebral atherosclerosis may play an important role in the development of AD. When the ferroptosis inhibitor ferrostatin-1 (Fer-1) was administered to ApoE^-/-^ mice, the degree of lipid deposition in the artery walls was reduced. Moreover, Fer-1 normalised the expressions of ferroptosis indicators such as SLC7A11 and glutathione peroxidase 4 (GPX4). These data suggest that ferroptosis might play a role in atherogenesis **(Bai, Li et al. 2020)** and that AD patients might benefit from the systemic application of ferroptosis inhibitors.

Ferritin is an intracellular iron storage protein. More than 50% of the molecular mass of iron-loaded ferritin is related to its iron content. In this protein, iron is present mainly as ferric hydroxide [Fe(OH)_3_]. When cellular ferritin levels exceed critical values, ferritin autophages *via* a process called ferritinophagy. This mechanism involves the cellular lysosomes and liberates free ionic iron. This iron subsequently accelerates ferroptosis **(Mancias, Wang et al. 2014, Hou, Xie et al. 2016, Wang, Chang et al. 2016)**. The nuclear receptor coactivator 4 (NCOA4) facilitates the transport of dysfunctional iron-loaded ferritin to the autophagosome, orchestrating its subsequent lysosomal degradation **(Mancias, Wang et al. 2014)**. The ferritin levels in the cerebro-spinal fluid (CSF) are positively correlated with the CSF APOE4 levels and APOE4 carriers have significantly elevated liquor levels than APOE3 carriers **(Ayton, Faux et al. 2015)**. Moreover, CSF ferritin concentration does negatively correlate with cognitive function **(Ayton, Diouf et al. 2018)**. In conclusion, iron metabolism and ferritinophagy are two key points in controlling ferroptotic cell death **(Figure 4)**.

CONCLUSIONs and perspectives

Alzheimer’s disease (AD) is one of the most prevalent neurodegenerative disorders and many risk factors have been identified for this disease. It is characterised by premature neuronal death, but our mechanistic understanding of the molecular processes leading to cell death remained unclear for a long time. Epidemiological studies suggested that polymorphism at the APOE gene locus is one of the most relevant risk factors for AD, but how this genetic polymorphism induces cell death remained a mystery for many years. Today, we know that the polymorphism of the *APOE* gene, in particular the presence of the APOE4 allele, alters neuronal iron homeostasis leading to excessive intracellular iron deposition. These iron deposits induce oxidative stress, which leads to uncontrolled lipid peroxidation. Under normal conditions the hydroperoxy lipids are rapidly reduced to the corresponding hydroxy derivatives by the catalytic activity of glutathione peroxidases. However, when the cellular reductive capacity is impaired the hydroperoxy lipids are decomposed via free-radical mediated secondary reactions and products of these secondary rections may trigger ferroptotic signaling leading to neuronal cell death. Pharmacological interference with the intracellular ferroptotic signaling is likely to prevent neuronal ferroptosis and thus, AD patients might benefit from such therapeutic intervention. Unfortunately, for the moment no neuron-specific ferroptosis inhibitors are available but the search for such compounds is underway. Developing such inhibitor it should always be kept in mind that the use of unspecific ferroptosis inhibitors, which are currently available (ferrostatin, liproxstatin), might be dangerous since ferroptosis has been implicated in killing cancer cells and thus, the use of unspecific ferroptosis inhibitors might activate tumor development and metastasis.

**List of abbreviations**

According to the recommendations of the Nomenclature Committee of the International Union of Biochemistry and Molecular Biology proteins should be named according to encoding genes as long as the genes mainly encode for a single protein. The names of genes or of corresponding alleles should be italized (*APOE4*) whereas the encoded proteins should be named in normal face (APOE4). Human genes and proteins should be given in capital letters (*APOE4* for the gene, APOE4 for the protein), whereas the mouse orthologs should be labeled in small letters (*apoE4* for the mouse gene, apoE4 for the mouse protein). If the species are not defined the human nomenclature should be used (capital letters)

Alzheimer disease, AD; Apolipoprotein E, APOE; beta-amyloid, Aβ; neurofibrillary tangles, NFT; early-onset form of AD, FAD; presenilin 1, *Psen1*; presenilin 2, *Psen2*; amyloid precursor protein, APP; Late onset-AD, LOAD; low-density-lipoprpotein-receptor, LDLR; ATP-binding assette A1, ABCA1; low-density lipoprotein receptor-related protein-1, LRP1; heparin sulphate proteoglycan, HSPG; blood-brain-barrier, BBB; Insulin-degrading enzyme, IDE; angiotensin converting enzyme, ACE; neprilysin, Nep; receptor for advanced glycosylation end products, RAGE; tau-proteins, tubulin associated units; microtubule associated protein tau, *MAPT*; extracellular signal-regulated kinase, ERK; eicosapentaenoic acid, EPA; docosahexaenoic acid, DHA; Mitochondria-associated membranes, MAM; [endoplasmic reticulum](https://en.wikipedia.org/wiki/Endoplasmic_reticulum), ER; Prostaglandin G synthase, PTGS; cyclooxygenase, COX; Arachidonic acid lipoxygenase, ALOX; major cannabinoid recepotors, CB; tetrahydrocannabinol, THC; cannabidiol, CBD; anandamide, AEA; 2-arachidonylglycerols, 2-AG; sphingosine-1-phosphate, S1P; C-reactive protein, CRP; triggering receptor expressed on myeloid cells 2, TREM2; neuroinflammatory phenotypes, NIP; vascular endothelial growth factor, VEGF; low-density lipoprotein receptor-related protein-1, LPRP1; mitochondrial permeability transition pore, mPTP; reactive oxygen species, ROS; Cyclophilin D, CypD; positron emission tomography, PET; cerebral metabolic rate for glucose, CMRgl; polyunsaturated fatty acids, PUFAs; malondialdehyde, MDA; hydroxynonenal, HNE; glutathione peroxidase 4, GPX4; glutathione, GSH; iron response element, IRE; presenilin enhancer-2, PEN2; divalent metal ion transporter 1, DMT1; glycogen synthase kinase-3β, GSK3β; cyclin-dependent protein kinase 5, CDK5; nuclear receptor coactivator 4, NCOA4; cerebro-spinal fluid, CSF; GS, glutathione synthase; GCL, glutamate-cysteine ligase.

**Declarations**

*a) Ethics approval*: Not applicable (this is a review paper)

*b) Consent of publication:* Not applicable (this is a review paper*)*

*c) Data availability:*All data refered to in this review paper are publically available.

*d) Competing interests:* The authors declare that they have no conflicts of interest with the content of this paper.

*e) Funding:* There was no extramural funding for this paper.

*f) Autor contribution:* PF drafted the original ms and all authors (PF, HK, SA) contributed to the final version. PF structured the original figures and HK revised them.

*g) Acknowledgement:* Not applicable

h) *Author information*: PF is a graduate student of the University of Teheran who spend a 12 moths sabbatical in the lab of HK at the Department of Biochemistry, Charite - University Medicine Berlin to complete her PhD thesis.

| **Effects of apoE4** | **Mechanism of action** | **Type of study** |
| --- | --- | --- |
| Aβ metabolism | APOE4 induces Aβ plaque formation (Koistinaho, Lin et al. 2004, Fernandez, Hamby et al. 2019). | In vitro,  Clinical trials |
|  | APOE4 reduces monomeric Aβ (Huynh, Davis et al. 2017), causes decrease in Aβ clearance (Safieh, Korczyn et al. 2019) and activates gamma-secretase (Lane-Donovan and Herz 2017). | In vivo |
|  | APOE4 induces formation of APOE-fragment/Aβ heterodimers (Mouchard, Boutonnet et al. 2019) review and Aβ complexes in brain vasculature (Martel, Mackic et al. 1997, Kim, Basak et al. 2009). | In vivo |
| Tau phosphorylation | APOE4 enhances the phosphorylation of tau (Brecht, Harris et al. 2004) | In vivo |
|  | APOE4 reduces monomeric Aβ (Huynh, Davis et al. 2017), causes decrease in Aβ clearance (Safieh, Korczyn et al. 2019) and activates gamma-secretase (Lane-Donovan and Herz 2017). | In vivo |
|  | APOE4 proteolysis fragments induce hyperphosphorylation of tau proteins (Huang, Liu et al. 2001, Harris, Brecht et al. 2003). | In vitro  In vivo |
|  | APOE4 interacts directly with tau in the cytoplasm to induce its hyperphosphorylation (Harris, Brecht et al. 2004) | In vivo |
| Lipid metabolism | APOE4 increases the production of phospholipids and cholesteryl esters by MAM functions (Tambini, Pera et al. 2016). | In vitro |
|  | APOE4 deficiency causes the lack of lipid transport in the brain (Carter, Letronne et al. 2017). | In vivo |
| Neuroinflammation | APOE4 induces the expression of IL-1β, more than APOE3 (Guo, LaDu et al. 2004, Dorey, Chang et al. 2014). | In vitro  In vivo |
|  | APOE4 pro-inflammatory role is stronger than APOE3 (Guo, LaDu et al. 2004). | In vivo |
|  | APOE4 causes the activation of the inflammatory receptor and the suppression of the anti-inflammatory receptor (Li, Montine et al. 2015) | In vitro |
|  | APOE4 increase inflammation (Ophir, Amariglio et al. 2005, Cash, Kuhel et al. 2012, Dorey, Chang et al. 2014, Rodriguez, Tai et al. 2014, Du, Jia et al. 2015, Li, Montine et al. 2015) and neuro-inflammation (Lukiw, Zhao et al. 2008, Teter, LaDu et al. 2016). | In vitro, in vivo  Clinical trials  in vitro |
| Vascular integrity/function | APOE4 increases the risk of vascular dementia and atherosclerosis (Mahley, Weisgraber et al. 2009, Rohn 2014). | In vivo |
|  | APOE4 carriers show cerebrovascular dysfunction (Mielke, Leoutsakos et al. 2011, Bell 2012, Tai,(Montagne, Nation et al. 2020). | Meta analyze  Clinical trials |
|  | APOE4 induces accumulation of Aβ, fibrin and fibrinogen in the neuro-vasculature of AD patients (Cortes-Canteli, Zamolodchikov et al. 2012, Rannikmäe, Kalaria et al. 2014). | In vivo |
| Insulin and VEGF signaling | APOE4 disrupts insulin metabolism in neurons (Ong, Chan et al. 2014, Traversy, Zhao, Liu et al. 2017). | In vivo  Clinical trials |
| Synaptic plasticity | APOE4 inhibits neurite outgrowth in some cases (Nathan, Jiang et al. 2002). | In vitro |
|  | APOE3 activates LRP1, APOE receptor, heparin sulfate proteoglycan more than APOE4 (Hayashi, Campenot et al. 2007). | Clinical trials |
| Mitochondrial function | APOE4 fragments reduce electron transport and the function of ATP synthase (Huang and Mucke 2012, Liu, Kanekiyo et al. 2013). | Clinical trials |
|  | Expression of mitochondrial respiratory complexes I, IV, and V are down-regulated in APOE4 carriers (Chang, ran Ma et al. 2005, Chen, Ji et al. 2011). | In vivo |
|  | APOE4 leads to mitochondrial dysfunction and enhanced levels of ROS production (Liang, Hang et al. 2021). | In vivo and In vitro |

|  |  |
| --- | --- |

References

Abate, G., D. Uberti and S. Tambaro (2021). "Potential and Limits of Cannabinoids in Alzheimer's Disease Therapy." Biology (Basel) **10**(6).

Abyadeh, M., V. Gupta, J. A. Paulo, V. Gupta, N. Chitranshi, A. Godinez, D. Saks, M. Hasan, A. Amirkhani, M. McKay, G. H. Salekdeh, P. A. Haynes, S. L. Graham and M. Mirzaei (2021). "A Proteomic View of Cellular and Molecular Effects of Cannabis." Biomolecules **11**(10).

Anand, R., K. D. Gill and A. A. Mahdi (2014). "Therapeutics of Alzheimer's disease: Past, present and future." Neuropharmacology **76**: 27-50.

Ando, K., Q. Laborde, A. Lazar, D. Godefroy, I. Youssef, M. Amar, A. Pooler, M.-C. Potier, B. Delatour and C. Duyckaerts (2014). "Inside Alzheimer brain with CLARITY: senile plaques, neurofibrillary tangles and axons in 3-D." Acta neuropathologica **128**(3): 457-459.

Ang, L. S., R. P. Cruz, A. Hendel and D. J. Granville (2008). "Apolipoprotein E, an important player in longevity and age-related diseases." Exp Gerontol **43**(7): 615-622.

Asaro, A., R. Sinha, M. Bakun, O. Kalnytska, A. S. Carlo-Spiewok, T. Rubel, A. Rozeboom, M. Dadlez, B. Kaminska, E. Aronica, A. R. Malik and T. E. Willnow (2021). "ApoE4 disrupts interaction of sortilin with fatty acid-binding protein 7 essential to promote lipid signaling." J Cell Sci **134**(20).

Ashraf, A. and P.-W. So (2020). "Spotlight on ferroptosis: iron-dependent cell death in alzheimer’s disease." Frontiers in Aging Neuroscience **12**: 196.

Association, A. s. (2018). "2018 Alzheimer's disease facts and figures." Alzheimer's & Dementia **14**(3): 367-429.

Aydin, D., S. W. Weyer and U. C. Müller (2012). "Functions of the APP gene family in the nervous system: insights from mouse models." Experimental brain research **217**: 423-434.

Ayton, S., I. Diouf and A. I. Bush (2018). "Evidence that iron accelerates Alzheimer’s pathology: a CSF biomarker study." J Neurol Neurosurg Psychiatry **89**(5): 456-460.

Ayton, S., N. G. Faux and A. I. Bush (2015). "Ferritin levels in the cerebrospinal fluid predict Alzheimer’s disease outcomes and are regulated by APOE." Nature communications **6**(1): 1-9.

Ayton, S., N. G. Faux, A. I. Bush, M. W. Weiner, P. Aisen, R. Petersen, C. R. Jack Jr, W. Jagust, J. Q. Trojanowki and A. W. Toga (2015). "Ferritin levels in the cerebrospinal fluid predict Alzheimer’s disease outcomes and are regulated by APOE." Nature communications **6**: 6760.

Bagyinszky, E., V. V. Giau and S. A. An (2020). "Transcriptomics in Alzheimer's Disease: Aspects and Challenges." Int J Mol Sci **21**(10).

Bai, T., M. X. Li, Y. F. Liu, Z. T. Qiao and Z. W. Wang (2020). "Inhibition of ferroptosis alleviates atherosclerosis through attenuating lipid peroxidation and endothelial dysfunction in mouse aortic endothelial cell." Free Radical Biology and Medicine **160**: 92-102.

Balejcikova, L., K. Siposova, P. Kopcansky and I. Safarik (2018). "Fe (II) formation after interaction of the amyloid β-peptide with iron-storage protein ferritin." Journal of biological physics **44**(3): 237-243.

Bandyopadhyay, S., L. Goldstein, D. Lahiri and J. Rogers (2007). "Role of the APP non-amyloidogenic signaling pathway and targeting α-secretase as an alternative drug target for treatment of Alzheimer's disease." Current medicinal chemistry **14**(27): 2848-2864.

Bao, W. D., P. Pang, X. T. Zhou, F. Hu, W. Xiong, K. Chen, J. Wang, F. Wang, D. Xie, Y. Z. Hu, Z. T. Han, H. H. Zhang, W. X. Wang, P. T. Nelson, J. G. Chen, Y. Lu, H. Y. Man, D. Liu and L. Q. Zhu (2021). "Loss of ferroportin induces memory impairment by promoting ferroptosis in Alzheimer's disease." Cell Death Differ **28**(5): 1548-1562.

Basavarajappa, B. S., M. Shivakumar, V. Joshi and S. Subbanna (2017). "Endocannabinoid system in neurodegenerative disorders." J Neurochem **142**(5): 624-648.

Belaidi, A. A., S. Masaldan, A. Southon, P. Kalinowski, K. Acevedo, A. T. Appukuttan, S. Portbury, P. Lei, P. Agarwal, S. E. Leurgans, J. Schneider, M. Conrad, A. I. Bush and S. Ayton (2022). "Apolipoprotein E potently inhibits ferroptosis by blocking ferritinophagy." Mol Psychiatry.

Bell, R. D. (2012). "The imbalance of vascular molecules in Alzheimer's disease." Journal of Alzheimer's Disease **32**(3): 699-709.

Benedet, J. and T. Shibamoto (2008). "Role of transition metals, Fe (II), Cr (II), Pb (II), and Cd (II) in lipid peroxidation." Food chemistry **107**(1): 165-168.

Benelli, R., R. Venè and N. Ferrari (2018). "Prostaglandin-endoperoxide synthase 2 (cyclooxygenase-2), a complex target for colorectal cancer prevention and therapy." Transl Res **196**: 42-61.

Bhaskar, K., S.-H. Yen and G. Lee (2005). "Disease-related modifications in tau affect the interaction between Fyn and Tau." Journal of Biological Chemistry **280**(42): 35119-35125.

Bhattacharyya, S., P. Fusar-Poli, S. Borgwardt, R. Martin-Santos, C. Nosarti, C. O'Carroll, P. Allen, M. L. Seal, P. C. Fletcher, J. A. Crippa, V. Giampietro, A. Mechelli, Z. Atakan and P. McGuire (2009). "Modulation of mediotemporal and ventrostriatal function in humans by Delta9-tetrahydrocannabinol: a neural basis for the effects of Cannabis sativa on learning and psychosis." Arch Gen Psychiatry **66**(4): 442-451.

Biamonti, G., A. Amato, E. Belloni, A. Di Matteo, L. Infantino, D. Pradella and C. Ghigna (2021). "Alternative splicing in Alzheimer's disease." Aging Clin Exp Res **33**(4): 747-758.

Biringer, R. G. (2019). "The Role of Eicosanoids in Alzheimer's Disease." Int J Environ Res Public Health **16**(14).

Biringer, R. G. (2019). "The role of eicosanoids in Alzheimer’s disease." International Journal of Environmental Research and Public Health **16**(14): 2560.

Brecht, W. J., F. M. Harris, S. Chang, I. Tesseur, G.-Q. Yu, Q. Xu, J. D. Fish, T. Wyss-Coray, M. Buttini and L. Mucke (2004). "Neuron-specific apolipoprotein e4 proteolysis is associated with increased tau phosphorylation in brains of transgenic mice." Journal of Neuroscience **24**(10): 2527-2534.

Bruce-Keller, A. J., Y.-J. Li, M. A. Lovell, P. J. Kraemer, D. S. Gary, R. R. Brown, W. R. Markesbery and M. P. Mattson (1998). "4-Hydroxynonenal, a product of lipid peroxidation, damages cholinergic neurons and impairs visuospatial memory in rats." Journal of Neuropathology & Experimental Neurology **57**(3): 257-267.

Cairns, J. A. (2007). "The coxibs and traditional nonsteroidal anti-inflammatory drugs: a current perspective on cardiovascular risks." Can J Cardiol **23**(2): 125-131.

Campagna, J., P. Spilman, B. Jagodzinska, D. S. Bai, A. Hatami, C. N. Zhu, T. Bilousova, M. Jun, C. J. Elias, J. Pham, G. Cole, M. J. LaDu, M. E. Jung, D. E. Bredesen and V. John (2018). "A small molecule ApoE4-targeted therapeutic candidate that normalizes sirtuin 1 levels and improves cognition in an Alzheimer's disease mouse model." Scientific Reports **8**.

Capdevila, J. H., J. R. Falck and R. C. Harris (2000). "Cytochrome P450 and arachidonic acid bioactivation. Molecular and functional properties of the arachidonate monooxygenase." J Lipid Res **41**(2): 163-181.

Cardoso, B. R., D. J. Hare, A. I. Bush and B. R. Roberts (2017). "Glutathione peroxidase 4: a new player in neurodegeneration?" Molecular Psychiatry **22**(3): 328-335.

Carter, A. Y., F. Letronne, N. F. Fitz, A. Mounier, C. M. Wolfe, K. N. Nam, V. L. Reeves, H. Kamboh, I. Lefterov and R. Koldamova (2017). "Liver X receptor agonist treatment significantly affects phenotype and transcriptome of APOE3 and APOE4 Abca1 haplo-deficient mice." PloS one **12**(2): e0172161.

Carter, G. W., P. R. Young, D. H. Albert, J. Bouska, R. Dyer, R. L. Bell, J. B. Summers and D. W. Brooks (1991). "5-lipoxygenase inhibitory activity of zileuton." J Pharmacol Exp Ther **256**(3): 929-937.

Cash, J. G., D. G. Kuhel, J. E. Basford, A. Jaeschke, T. K. Chatterjee, N. L. Weintraub and D. Y. Hui (2012). "Apolipoprotein E4 impairs macrophage efferocytosis and potentiates apoptosis by accelerating endoplasmic reticulum stress." Journal of Biological Chemistry **287**(33): 27876-27884.

Castellani, R. J., P. I. Moreira, G. Liu, J. Dobson, G. Perry, M. A. Smith and X. Zhu (2007). "Iron: the Redox-active center of oxidative stress in Alzheimer disease." Neurochemical research **32**(10): 1640-1645.

Castellano, J. M., J. Kim, F. R. Stewart, H. Jiang, R. B. DeMattos, B. W. Patterson, A. M. Fagan, J. C. Morris, K. G. Mawuenyega and C. Cruchaga (2011). "Human apoE isoforms differentially regulate brain amyloid-β peptide clearance." Science translational medicine **3**(89): 89ra57-89ra57.

Chawla, A., W. A. Boisvert, C.-H. Lee, B. A. Laffitte, Y. Barak, S. B. Joseph, D. Liao, L. Nagy, P. A. Edwards and L. K. Curtiss (2001). "A PPARγ-LXR-ABCA1 pathway in macrophages is involved in cholesterol efflux and atherogenesis." Molecular cell **7**(1): 161-171.

Chen, J., Q. Li and J. Wang (2011). "Topology of human apolipoprotein E3 uniquely regulates its diverse biological functions." Proc Natl Acad Sci U S A **108**(36): 14813-14818.

Chen, K., X. B. Jiang, M. X. Wu, X. M. Cao, W. D. Bao and L. Q. Zhu (2021). "Ferroptosis, a Potential Therapeutic Target in Alzheimer's Disease." Frontiers in Cell and Developmental Biology **9**.

Coles, M., G. Z. Steiner-Lim and T. Karl (2022). "Therapeutic properties of multi-cannabinoid treatment strategies for Alzheimer's disease." Front Neurosci **16**: 962922.

Corder, E. H., A. M. Saunders, W. J. Strittmatter, D. E. Schmechel, P. C. Gaskell, G. Small, A. D. Roses, J. Haines and M. A. Pericak-Vance (1993). "Gene dose of apolipoprotein E type 4 allele and the risk of Alzheimer's disease in late onset families." Science **261**(5123): 921-923.

Cortes-Canteli, M., D. Zamolodchikov, H. J. Ahn, S. Strickland and E. H. Norris (2012). "Fibrinogen and altered hemostasis in Alzheimer's disease." Journal of Alzheimer's Disease **32**(3): 599-608.

Crippa, J. A., A. W. Zuardi, G. E. Garrido, L. Wichert-Ana, R. Guarnieri, L. Ferrari, P. M. Azevedo-Marques, J. E. Hallak, P. K. McGuire and G. Filho Busatto (2004). "Effects of cannabidiol (CBD) on regional cerebral blood flow." Neuropsychopharmacology **29**(2): 417-426.

Czapski, G. A., K. Czubowicz, J. B. Strosznajder and R. P. Strosznajder (2016). "The Lipoxygenases: Their Regulation and Implication in Alzheimer's Disease." Neurochem Res **41**(1-2): 243-257.

Czubowicz, K., H. Jęśko, P. Wencel, W. J. Lukiw and R. P. Strosznajder (2019). "The Role of Ceramide and Sphingosine-1-Phosphate in Alzheimer's Disease and Other Neurodegenerative Disorders." Mol Neurobiol **56**(8): 5436-5455.

D'Arcy, M. S. (2019). "Cell death: a review of the major forms of apoptosis, necrosis and autophagy." Cell Biol Int **43**(6): 582-592.

Dehghan, A., R. C. Pinto, I. Karaman, J. Huang, B. R. Durainayagam, M. Ghanbari, A. Nazeer, Q. Zhong, S. Liggi, L. Whiley, R. Mustafa, M. Kivipelto, A. Solomon, T. Ngandu, T. Kanekiyo, T. Aikawa, C. I. Radulescu, S. J. Barnes, G. Graça, E. Chekmeneva, S. Camuzeaux, M. R. Lewis, M. R. Kaluarachchi, M. A. Ikram, E. Holmes, I. Tzoulaki, P. M. Matthews, J. L. Griffin and P. Elliott (2022). "Metabolome-wide association study on ABCA7 indicates a role of ceramide metabolism in Alzheimer's disease." Proc Natl Acad Sci U S A **119**(43): e2206083119.

Deroux, A., A. Madelon, B. Colombe, M. Lugosi and L. Bouillet (2022). "[An alarming necrosis]." Rev Med Interne **43**(12): 752-754.

Dimitrow, P. P. (2009). "Pleiotropic, cardioprotective effects of omega-3 polyunsaturated fatty acids." Mini Reviews in Medicinal Chemistry **9**(9): 1030-1039.

Dixon, S. J., K. M. Lemberg, M. R. Lamprecht, R. Skouta, E. M. Zaitsev, C. E. Gleason, D. N. Patel, A. J. Bauer, A. M. Cantley and W. S. Yang (2012). "Ferroptosis: an iron-dependent form of nonapoptotic cell death." Cell **149**(5): 1060-1072.

Dixon, S. J. and B. R. Stockwell (2019). "The hallmarks of ferroptosis." Annual Review of Cancer Biology **3**: 35-54.

Dobrian, A. D., D. C. Lieb, Q. Ma, J. W. Lindsay, B. K. Cole, K. Ma, S. K. Chakrabarti, N. S. Kuhn, S. D. Wohlgemuth, M. Fontana and J. L. Nadler (2010). "Differential expression and localization of 12/15 lipoxygenases in adipose tissue in human obese subjects." Biochem Biophys Res Commun **403**(3-4): 485-490.

Dorey, E., N. Chang, Q. Y. Liu, Z. Yang and W. Zhang (2014). "Apolipoprotein E, amyloid-beta, and neuroinflammation in Alzheimer’s disease." Neuroscience bulletin **30**(2): 317-330.

Dries, D. R., G. Yu and J. Herz (2012). "Extracting β-amyloid from Alzheimer's disease." Proceedings of the National Academy of Sciences **109**(9): 3199-3200.

Du, Z., H. Jia, J. Liu, X. Zhao and W. Xu (2015). "Effects of three hydrogen-rich liquids on hemorrhagic shock in rats." journal of surgical research **193**(1): 377-382.

Dubois, R. N., S. B. Abramson, L. Crofford, R. A. Gupta, L. S. Simon, L. B. Van De Putte and P. E. Lipsky (1998). "Cyclooxygenase in biology and disease." The FASEB journal **12**(12): 1063-1073.

El-Agnaf, O. M., D. S. Mahil, B. P. Patel and B. M. Austen (2000). "Oligomerization and toxicity of β-amyloid-42 implicated in Alzheimer's disease." Biochemical and biophysical research communications **273**(3): 1003-1007.

Epp, N., G. Fürstenberger, K. Müller, S. de Juanes, M. Leitges, I. Hausser, F. Thieme, G. Liebisch, G. Schmitz and P. Krieg (2007). "12R-lipoxygenase deficiency disrupts epidermal barrier function." The Journal of cell biology **177**(1): 173-182.

Evin, G. and A. Weidemann (2002). "Biogenesis and metabolism of Alzheimer’s disease Aβ amyloid peptides." Peptides **23**(7): 1285-1297.

Farhadieh, M. E. and K. Ghaedi (2023). "Analyzing alternative splicing in Alzheimer's disease postmortem brain: a cell-level perspective." Front Mol Neurosci **16**: 1237874.

Fiorucci, S., E. Antonelli and A. Morelli (2001). "Mechanism of non-steroidal anti-inflammatory drug-gastropathy." Dig Liver Dis **33 Suppl 2**: S35-43.

Firuzi, O. and D. Praticò (2006). "Coxibs and Alzheimer's disease: should they stay or should they go?" Ann Neurol **59**(2): 219-228.

Fukami, S., K. Watanabe, N. Iwata, J. Haraoka, B. Lu, N. P. Gerard, C. Gerard, P. Fraser, D. Westaway and P. S. George-Hyslop (2002). "Aβ-degrading endopeptidase, neprilysin, in mouse brain: synaptic and axonal localization inversely correlating with Aβ pathology." Neuroscience research **43**(1): 39-56.

Funk, C. D. (2001). "Prostaglandins and leukotrienes: advances in eicosanoid biology." Science **294**(5548): 1871-1875.

Funk, C. D., D. S. Keeney, E. H. Oliw, W. E. Boeglin and A. R. Brash (1996). "Functional expression and cellular localization of a mouse epidermal lipoxygenase." J Biol Chem **271**(38): 23338-23344.

Gaiarsa, J. L., O. Caillard and Y. Ben-Ari (2002). "Long-term plasticity at GABAergic and glycinergic synapses: mechanisms and functional significance." Trends Neurosci **25**(11): 564-570.

Galluzzi, L., I. Vitale, S. A. Aaronson, J. M. Abrams, D. Adam, P. Agostinis, E. S. Alnemri, L. Altucci, I. Amelio and D. W. Andrews (2018). "Molecular mechanisms of cell death: recommendations of the Nomenclature Committee on Cell Death 2018." Cell Death & Differentiation **25**(3): 486-541.

Gao, M., P. Monian, N. Quadri, R. Ramasamy and X. Jiang (2015). "Glutaminolysis and transferrin regulate ferroptosis." Molecular cell **59**(2): 298-308.

Gatt, A., H. Lee, G. Williams, S. Thuret and C. Ballard (2019). "Expression of neurogenic markers in Alzheimer's disease: a systematic review and metatranscriptional analysis." Neurobiol Aging **76**: 166-180.

Genis, I., I. Gordon, E. Sehayek and D. M. Michaelson (1995). "Phosphorylation of tau in apolipoprotein E-deficient mice." Neurosci Lett **199**(1): 5-8.

Getz, G. S. and C. A. Reardon (2009). "Apoprotein E as a lipid transport and signaling protein in the blood, liver, and artery wall." J Lipid Res **50 Suppl**(Suppl): S156-161.

Giannakopoulos, P., F. Herrmann, T. Bussière, C. Bouras, E. Kövari, D. Perl, J. Morrison, G. Gold and P. Hof (2003). "Tangle and neuron numbers, but not amyloid load, predict cognitive status in Alzheimer’s disease." Neurology **60**(9): 1495-1500.

Goedert, M., C. Wischik, R. Crowther, J. Walker and A. Klug (1988). "Cloning and sequencing of the cDNA encoding a core protein of the paired helical filament of Alzheimer disease: identification as the microtubule-associated protein tau." Proceedings of the National Academy of Sciences **85**(11): 4051-4055.

Golabek, A. A., C. Soto, T. Vogel and T. Wisniewski (1996). "The interaction between apolipoprotein E and Alzheimer's amyloid beta-peptide is dependent on beta-peptide conformation." Journal of Biological Chemistry **271**(18): 10602-10606.

Golde, T. E., W. J. Streit and P. Chakrabarty (2013). "Alzheimer's disease risk alleles in TREM2 illuminate innate immunity in Alzheimer's disease." Alzheimers Res Ther **5**(3): 24.

GOODMAN, L. (1953). "Alzheimer's disease: a clinico-pathologic analysis of twenty-three cases with a theory on pathogenesis." The Journal of nervous and mental disease **118**(2): 97-130.

Greenow, K., N. J. Pearce and D. P. Ramji (2005). "The key role of apolipoprotein E in atherosclerosis." Journal of molecular medicine **83**: 329-342.

Guo, C., J.-L. Liu, Y.-G. Fan, Z.-S. Yang and Z.-Y. Wang (2018). "Iron and Alzheimer's disease: from pathogenesis to therapeutic implications." Frontiers in neuroscience **12**: 632.

Guo, C., P. Wang, M.-L. Zhong, T. Wang, X.-S. Huang, J.-Y. Li and Z.-Y. Wang (2013). "Deferoxamine inhibits iron induced hippocampal tau phosphorylation in the Alzheimer transgenic mouse brain." Neurochemistry international **62**(2): 165-172.

Guo, L., M. J. LaDu and L. J. Van Eldik (2004). "A dual role for apolipoprotein E in neuroinflammation." Journal of Molecular Neuroscience **23**(3): 205-212.

Gustavsson, A., N. Norton, T. Fast, L. Frolich, J. Georges, D. Holzapfel, T. Kirabali, P. Krolak-Salmon, P. M. Rossini, M. T. Ferretti, L. Lanman, A. S. Chadha and W. M. van der Flier (2023). "Global estimates on the number of persons across the Alzheimer's disease continuum." Alzheimers Dement **19**(2): 658-670.

Haass, C. and D. J. Selkoe (2007). "Soluble protein oligomers in neurodegeneration: lessons from the Alzheimer's amyloid β-peptide." Nature reviews Molecular cell biology **8**(2): 101.

Hafez, D., J. Y. Huang, A. M. Huynh, S. Valtierra, E. Rockenstein, A. M. Bruno, B. Lu, L. DesGroseillers, E. Masliah and R. A. Marr (2011). "Neprilysin-2 is an important β-amyloid degrading enzyme." The American journal of pathology **178**(1): 306-312.

Hallenborg, P., C. Jørgensen, R. K. Petersen, S. Feddersen, P. Araujo, P. Markt, T. Langer, G. Furstenberger, P. Krieg and A. Koppen (2010). "Epidermis-type lipoxygenase 3 regulates adipocyte differentiation and peroxisome proliferator-activated receptor γ activity." Molecular and cellular biology.

Hallenborg, P., C. Jørgensen, R. K. Petersen, S. Feddersen, P. Araujo, P. Markt, T. Langer, G. Furstenberger, P. Krieg, A. Koppen, E. Kalkhoven, L. Madsen and K. Kristiansen (2010). "Epidermis-type lipoxygenase 3 regulates adipocyte differentiation and peroxisome proliferator-activated receptor gamma activity." Mol Cell Biol **30**(16): 4077-4091.

Hampel, H., K. Blennow, L. M. Shaw, Y. C. Hoessler, H. Zetterberg and J. Q. Trojanowski (2010). "Total and phosphorylated tau protein as biological markers of Alzheimer’s disease." Experimental gerontology **45**(1): 30-40.

Han, X., S. Rozen, S. H. Boyle, C. Hellegers, H. Cheng, J. R. Burke, K. A. Welsh-Bohmer, P. M. Doraiswamy and R. Kaddurah-Daouk (2011). "Metabolomics in early Alzheimer's disease: identification of altered plasma sphingolipidome using shotgun lipidomics." PLoS One **6**(7): e21643.

Hanger, D. P., B. H. Anderton and W. Noble (2009). "Tau phosphorylation: the therapeutic challenge for neurodegenerative disease." Trends in molecular medicine **15**(3): 112-119.

Hannun, Y. A. and L. M. Obeid (2018). "Sphingolipids and their metabolism in physiology and disease." Nat Rev Mol Cell Biol **19**(3): 175-191.

Hardy, J. and D. J. Selkoe (2002). "The amyloid hypothesis of Alzheimer's disease: progress and problems on the road to therapeutics." science **297**(5580): 353-356.

Harris, F. M., W. J. Brecht, Q. Xu, R. W. Mahley and Y. Huang (2004). "Increased tau phosphorylation in apolipoprotein E4 transgenic mice is associated with activation of extracellular signal-regulated kinase modulation by Zinc." Journal of Biological Chemistry **279**(43): 44795-44801.

Harris, F. M., W. J. Brecht, Q. Xu, I. Tesseur, L. Kekonius, T. Wyss-Coray, J. D. Fish, E. Masliah, P. C. Hopkins and K. Scearce-Levie (2003). "Carboxyl-terminal-truncated apolipoprotein E4 causes Alzheimer's disease-like neurodegeneration and behavioral deficits in transgenic mice." Proceedings of the National Academy of Sciences **100**(19): 10966-10971.

Hauser, P. S., V. Narayanaswami and R. O. Ryan (2011). "Apolipoprotein E: from lipid transport to neurobiology." Progress in lipid research **50**(1): 62-74.

Hayashi, H., R. B. Campenot, D. E. Vance and J. E. Vance (2007). "Apolipoprotein E-containing lipoproteins protect neurons from apoptosis via a signaling pathway involving low-density lipoprotein receptor-related protein-1." Journal of Neuroscience **27**(8): 1933-1941.

Hazekamp, A., J. T. Fischedick, M. Díez, A. Lubbe and R. L. Ruhaak (2010). "3.24—Chemistry of Cannabis." Comprehensive natural products II **3**: 1033-1084.

He, F., Z. Chen, W. Deng, T. Zhan, X. Huang, Y. Zheng and H. Yang (2021). "Development and validation of a novel ferroptosis-related gene signature for predicting prognosis and immune microenvironment in head and neck squamous cell carcinoma." Int Immunopharmacol **98**: 107789.

Hoe, H. S., J. Freeman and G. W. Rebeck (2006). "Apolipoprotein E decreases tau kinases and phospho-tau levels in primary neurons." Mol Neurodegener **1**: 18.

Hohman, T. J., L. Dumitrescu, L. L. Barnes, M. Thambisetty, G. Beecham, B. Kunkle, K. A. Gifford, W. S. Bush, L. B. Chibnik and S. Mukherjee (2018). "Sex-specific association of apolipoprotein e with cerebrospinal fluid levels of tau." JAMA neurology **75**(8): 989-998.

Hou, T. T., Y. D. Han, L. Cong, C. C. Liu, X. Y. Liang, F. Z. Xue and Y. F. Du (2020). "Apolipoprotein E Facilitates Amyloid-beta Oligomer-Induced Tau Phosphorylation." J Alzheimers Dis **74**(2): 521-534.

Hou, W., Y. Xie, X. Song, X. Sun, M. T. Lotze, H. J. Zeh III, R. Kang and D. Tang (2016). "Autophagy promotes ferroptosis by degradation of ferritin." Autophagy **12**(8): 1425-1428.

Houtkooper, R. H., E. Pirinen and J. Auwerx (2012). "Sirtuins as regulators of metabolism and healthspan." Nature Reviews Molecular Cell Biology **13**(4): 225-238.

Hrycay, E. G. and S. M. Bandiera (2012). "The monooxygenase, peroxidase, and peroxygenase properties of cytochrome P450." Arch Biochem Biophys **522**(2): 71-89.

Huang, Y., X. Q. Liu, T. Wyss-Coray, W. J. Brecht, D. A. Sanan and R. W. Mahley (2001). "Apolipoprotein E fragments present in Alzheimer's disease brains induce neurofibrillary tangle-like intracellular inclusions in neurons." Proceedings of the National Academy of Sciences **98**(15): 8838-8843.

Huang, Y. and L. Mucke (2012). "Alzheimer mechanisms and therapeutic strategies." Cell **148**(6): 1204-1222.

Huang, Z. H., D. Gu and T. Mazzone (2009). "Role of adipocyte-derived apoE in modulating adipocyte size, lipid metabolism, and gene expression in vivo." Am J Physiol Endocrinol Metab **296**(5): E1110-1119.

Hüttenrauch, M., S. Baches, J. Gerth, T. A. Bayer, S. Weggen and O. Wirths (2015). "Neprilysin deficiency alters the neuropathological and behavioral phenotype in the 5XFAD mouse model of Alzheimer's disease." Journal of Alzheimer's Disease **44**(4): 1291-1302.

Huynh, T.-P. V., A. A. Davis, J. D. Ulrich and D. M. Holtzman (2017). "Apolipoprotein E and Alzheimer’s disease: the influence of apolipoprotein E on amyloid-β and other amyloidogenic proteins." Journal of lipid research **58**(5): 824-836.

Ivanov, I., K. R. Kakularam, E. V. Shmendel, M. Rothe, P. Aparoy, D. Heydeck and H. Kuhn (2021). "Oxygenation of endocannabinoids by mammalian lipoxygenase isoforms." Biochimica et Biophysica Acta (BBA)-Molecular and Cell Biology of Lipids **1866**(6): 158918.

Jakaria, M., A. A. Belaidi, A. I. Bush and S. Ayton (2021). "Ferroptosis as a mechanism of neurodegeneration in Alzheimer's disease." Journal of Neurochemistry **159**(5): 804-825.

Jendresen, C., V. Årskog, M. R. Daws and L. N. Nilsson (2017). "The Alzheimer's disease risk factors apolipoprotein E and TREM2 are linked in a receptor signaling pathway." J Neuroinflammation **14**(1): 59.

Jiang, W., Y. Zhang, F. Meng, B. Lian, X. Chen, X. Yu, E. Dai, S. Wang, X. Liu, X. Li, L. Wang and X. Li (2013). "Identification of active transcription factor and miRNA regulatory pathways in Alzheimer's disease." Bioinformatics **29**(20): 2596-2602.

Jofre‐Monseny, L., A. M. Minihane and G. Rimbach (2008). "Impact of apoE genotype on oxidative stress, inflammation and disease risk." Molecular nutrition & food research **52**(1): 131-145.

Kagerer, S. M., J. M. van Bergen, X. Li, F. C. Quevenco, A. F. Gietl, S. Studer, V. Treyer, R. Meyer, P. A. Kaufmann and R. M. Nitsch (2020). "APOE4 moderates effects of cortical iron on synchronized default mode network activity in cognitively healthy old‐aged adults." Alzheimer's & Dementia: Diagnosis, Assessment & Disease Monitoring **12**(1): e12002.

Kaur, R., S. R. Ambwani and S. Singh (2016). "Endocannabinoid System: A Multi-Facet Therapeutic Target." Curr Clin Pharmacol **11**(2): 110-117.

Kim, J., J. M. Basak and D. M. Holtzman (2009). "The role of apolipoprotein E in Alzheimer's disease." Neuron **63**(3): 287-303.

Kleuser, B. (2018). "The Enigma of Sphingolipids in Health and Disease." Int J Mol Sci **19**(10).

Kulmacz, R. J. (1998). "Cellular regulation of prostaglandin H synthase catalysis." FEBS letters **430**(3): 154-157.

Küpper, C., J. Levin and T. Klopstock (2017). "Eisen im alternden Gehirn." Neuroradiologie Scan **7**(02): 129-142.

Lalla, R. and G. Donmez (2013). "The role of sirtuins in Alzheimer's disease." Front Aging Neurosci **5**: 16.

Lane-Donovan, C. and J. Herz (2017). "The ApoE receptors Vldlr and Apoer2 in central nervous system function and disease." Journal of lipid research **58**(6): 1036-1043.

Lane-Donovan, C., W. M. Wong, M. S. Durakoglugil, C. R. Wasser, S. Jiang, X. Xian and J. Herz (2016). "Genetic Restoration of Plasma ApoE Improves Cognition and Partially Restores Synaptic Defects in ApoE-Deficient Mice." J Neurosci **36**(39): 10141-10150.

Langenbach, R., S. G. Morham, H. F. Tiano, C. D. Loftin, B. I. Ghanayem, P. C. Chulada, J. F. Mahler, C. A. Lee, E. H. Goulding and K. D. Kluckman (1995). "Prostaglandin synthase 1 gene disruption in mice reduces arachidonic acid-induced inflammation and indomethacin-induced gastric ulceration." Cell **83**(3): 483-492.

Larkin, L., L. M. Khachigian and W. Jessup (2000). "Regulation of apolipoprotein E production in macrophages (review)." Int J Mol Med **6**(3): 253-258.

Lattanzio, F., L. Carboni, D. Carretta, R. Rimondini, S. Candeletti and P. Romualdi (2014). "Human apolipoprotein E4 modulates the expression of Pin1, Sirtuin 1, and Presenilin 1 in brain regions of targeted replacement apoE mice." Neuroscience **256**: 360-369.

Lecca, D., Y. J. Jung, M. T. Scerba, I. Hwang, Y. K. Kim, S. Kim, S. Modrow, D. Tweedie, S. C. Hsueh, D. Liu, W. Luo, E. Glotfelty, Y. Li, J. Y. Wang, Y. Luo, B. J. Hoffer, D. S. Kim, R. A. McDevitt and N. H. Greig (2022). "Role of chronic neuroinflammation in neuroplasticity and cognitive function: A hypothesis." Alzheimers Dement **18**(11): 2327-2340.

Li, J., F. Cao, H. L. Yin, Z. J. Huang, Z. T. Lin, N. Mao, B. Sun and G. Wang (2020). "Ferroptosis: past, present and future." Cell Death Dis **11**(2): 88.

Li, X., Y. Liu, Q. Zheng, G. Yao, P. Cheng, G. Bu, H. Xu and Y.-w. Zhang (2013). "Ferritin light chain interacts with PEN-2 and affects γ-secretase activity." Neuroscience letters **548**: 90-94.

Li, X., K. S. Montine, C. D. Keene and T. J. Montine (2015). "Different mechanisms of apolipoprotein E isoform–dependent modulation of prostaglandin E2 production and triggering receptor expressed on myeloid cells 2 (TREM2) expression after innate immune activation of microglia." The FASEB Journal **29**(5): 1754.

Li, X., K. S. Montine, C. D. Keene and T. J. Montine (2015). "Different mechanisms of apolipoprotein E isoform–dependent modulation of prostaglandin E2 production and triggering receptor expressed on myeloid cells 2 (TREM2) expression after innate immune activation of microglia." The FASEB Journal **29**(5): 1754-1762.

Liang, T., W. Hang, J. Chen, Y. Wu, B. Wen, K. Xu, B. Ding and J. Chen (2021). "ApoE4 (Δ272–299) induces mitochondrial‐associated membrane formation and mitochondrial impairment by enhancing GRP75-modulated mitochondrial calcium overload in neuron." Cell & Bioscience **11**: 1-14.

Lima, D., A. C. M. Hacke, J. Inaba, C. A. Pessôa and K. Kerman (2020). "Electrochemical detection of specific interactions between apolipoprotein E isoforms and DNA sequences related to Alzheimer's disease." Bioelectrochemistry **133**.

Liu, C.-C., T. Kanekiyo, H. Xu and G. Bu (2013). "Apolipoprotein E and Alzheimer disease: risk, mechanisms and therapy." Nature Reviews Neurology **9**(2): 106.

Liu, C.-C., T. Kanekiyo, H. Xu and G. Bu (2013). "Apolipoprotein E and Alzheimer disease: risk, mechanisms and therapy." Nature Reviews Neurology **9**(2): 106-118.

Liu, C.-C., N. Zhao, Y. Fu, N. Wang, C. Linares, C.-W. Tsai and G. Bu (2017). "ApoE4 accelerates early seeding of amyloid pathology." Neuron **96**(5): 1024-1032. e1023.

Liu, L., K. R. MacKenzie, N. Putluri, M. Maletić-Savatić and H. J. Bellen (2017). "The glia-neuron lactate shuttle and elevated ROS promote lipid synthesis in neurons and lipid droplet accumulation in glia via APOE/D." Cell metabolism **26**(5): 719-737. e716.

Liu, L., K. Zhang, H. Sandoval, S. Yamamoto, M. Jaiswal, E. Sanz, Z. Li, J. Hui, B. H. Graham and A. Quintana (2015). "Glial lipid droplets and ROS induced by mitochondrial defects promote neurodegeneration." Cell **160**(1-2): 177-190.

Liu, Q., M. A. Smith, J. Avilá, J. DeBernardis, M. Kansal, A. Takeda, X. Zhu, A. Nunomura, K. Honda and P. I. Moreira (2005). "Alzheimer-specific epitopes of tau represent lipid peroxidation-induced conformations." Free Radical Biology and Medicine **38**(6): 746-754.

Loewith, R., H. Riezman and N. Winssinger (2019). "Sphingolipids and membrane targets for therapeutics." Curr Opin Chem Biol **50**: 19-28.

Long, J. M. and D. M. Holtzman (2019). "Alzheimer Disease: An Update on Pathobiology and Treatment Strategies." Cell **179**(2): 312-339.

Lukiw, W. J., Y. Zhao and J. G. Cui (2008). "An NF-κB-sensitive micro RNA-146a-mediated inflammatory circuit in Alzheimer disease and in stressed human brain cells." Journal of Biological Chemistry **283**(46): 31315-31322.

Ma, A., J. Wang, L. Yang, Y. An and H. Zhu (2017). "AMPK activation enhances the anti-atherogenic effects of high density lipoproteins in apoE(-/-) mice." J Lipid Res **58**(8): 1536-1547.

Ma, A., J. Wang, L. Yang, Y. An and H. Zhu (2017). "AMPK activation enhances the anti-atherogenic effects of high density lipoproteins in apoE−/− mice." Journal of lipid research **58**(8): 1536-1547.

Maceyka, M. and S. Spiegel (2014). "Sphingolipid metabolites in inflammatory disease." Nature **510**(7503): 58-67.

Maeda, N. (2011). "Development of Apolipoprotein E–Deficient Mice." Arteriosclerosis, thrombosis, and vascular biology **31**(9): 1957-1962.

Mahley, R. W. (1988). "Apolipoprotein E: cholesterol transport protein with expanding role in cell biology." Science **240**(4852): 622-630.

Mahley, R. W. (1988). "Apolipoprotein E: cholesterol transport protein with expanding role in cell biology." Science **240**(4852): 622-630.

Mahley, R. W. and Y. Huang (2012). "Apolipoprotein e sets the stage: response to injury triggers neuropathology." Neuron **76**(5): 871-885.

Mahley, R. W., T. L. Innerarity, S. C. Rall, Jr. and K. H. Weisgraber (1984). "Plasma lipoproteins: apolipoprotein structure and function." J Lipid Res **25**(12): 1277-1294.

Mahley, R. W., B. P. Nathan and R. E. Pitas (1996). "Apolipoprotein E. Structure, function, and possible roles in Alzheimer's disease." Ann N Y Acad Sci **777**: 139-145.

Mahley, R. W., K. H. Weisgraber and Y. Huang (2009). "Apolipoprotein E: structure determines function, from atherosclerosis to Alzheimer's disease to AIDS." Journal of lipid research **50**(Supplement): S183-S188.

Mamun, A. A., M. S. Uddin, M. F. Bin Bashar, S. Zaman, Y. Begum, I. J. Bulbul, M. S. Islam, M. S. Sarwar, B. Mathew, M. S. Amran, G. Md Ashraf, M. N. Bin-Jumah, S. A. Mousa and M. M. Abdel-Daim (2020). "Molecular Insight into the Therapeutic Promise of Targeting APOE4 for Alzheimer's Disease." Oxid Med Cell Longev **2020**: 5086250.

Mancias, J. D., X. Wang, S. P. Gygi, J. W. Harper and A. C. Kimmelman (2014). "Quantitative proteomics identifies NCOA4 as the cargo receptor mediating ferritinophagy." Nature **509**(7498): 105.

Manelli, A. M., L. C. Bulfinch, P. M. Sullivan and M. J. LaDu (2007). "Aβ42 neurotoxicity in primary co-cultures: effect of apoE isoform and Aβ conformation." Neurobiology of aging **28**(8): 1139-1147.

Marbach-Breitrück, E., N. Rohwer, C. Infante-Duarte, S. Romero-Suarez, D. Labuz, H. Machelska, L. Kutzner, N. H. Schebb, M. Rothe, P. Reddanna, K. H. Weylandt, L. H. Wieler, D. Heydeck and H. Kuhn (2021). "Knock-In Mice Expressing a 15-Lipoxygenating Alox5 Mutant Respond Differently to Experimental Inflammation Than Reported Alox5(-/-) Mice." Metabolites **11**(10).

Martel, C. L., J. B. Mackic, E. Matsubara, S. Governale, C. Miguel, W. Miao, J. G. McComb, B. Frangione, J. Ghiso and B. V. Zlokovic (1997). "Isoform‐Specific Effects of Apolipoproteins E2, E3, and E4 on Cerebral Capillary Sequestration and Blood‐Brain Barrier Transport of Circulating Alzheimer's Amyloid β." Journal of neurochemistry **69**(5): 1995-2004.

Martens, H., J. Novotny, J. Oberstrass, T. L. Steck, P. Postlethwait and W. Nellen (2002). "RNAi in Dictyostelium: the role of RNA-directed RNA polymerases and double-stranded RNase." Mol Biol Cell **13**(2): 445-453.

Martínez‐Oliván, J., X. Arias‐Moreno, A. Velazquez‐Campoy, O. Millet and J. Sancho (2014). "LDL receptor/lipoprotein recognition: endosomal weakening of ApoB and ApoE binding to the convex face of the LR 5 repeat." The FEBS journal **281**(6): 1534-1546.

McGonigal, R., J. A. Barrie, D. Yao, M. McLaughlin, M. E. Cunningham, E. G. Rowan and H. J. Willison (2019). "Glial Sulfatides and Neuronal Complex Gangliosides Are Functionally Interdependent in Maintaining Myelinating Axon Integrity." J Neurosci **39**(1): 63-77.

Mercken, L. and J. P. Brion (1995). "Phosphorylation of tau protein is not affected in mice lacking apolipoprotein E." Neuroreport **6**(17): 2381-2384.

Mielke, M. M., J.-M. Leoutsakos, J. T. Tschanz, R. C. Green, Y. Tripodis, C. D. Corcoran, M. C. Norton and C. G. Lyketsos (2011). "Interaction between vascular factors and the APOE ε4 allele in predicting rate of progression in Alzheimer's disease." Journal of Alzheimer's Disease **26**(1): 127-134.

Momeni, P. and R. Ferrari (2010). "Genetic and blood biomarkers of Alzheimer’s disease." The Open Nuclear Medicine Journal **2**: 12-24.

Montagne, A., D. A. Nation, A. P. Sagare, G. Barisano, M. D. Sweeney, A. Chakhoyan, M. Pachicano, E. Joe, A. R. Nelson and L. M. D’Orazio (2020). "APOE4 leads to blood–brain barrier dysfunction predicting cognitive decline." Nature **581**(7806): 71-76.

Morris, M., S. Maeda, K. Vossel and L. Mucke (2011). "The many faces of tau." Neuron **70**(3): 410-426.

Mouchard, A., M.-C. Boutonnet, C. Mazzocco, N. Biendon and N. Macrez (2019). "ApoE-fragment/Aβ heteromers in the brain of patients with Alzheimer’s disease." Scientific reports **9**(1): 3989.

Mouchard, A., M.-C. Boutonnet, C. Mazzocco, N. Biendon, N. Macrez and N.-C. N. Network (2019). "ApoE-fragment/Aβ heteromers in the brain of patients with Alzheimer’s disease." Scientific Reports **9**(1): 3989.

Neve, R. L., P. Harris, K. S. Kosik, D. M. Kurnit and T. A. Donlon (1986). "Identification of cDNA clones for the human microtubule-associated protein tau and chromosomal localization of the genes for tau and microtubule-associated protein 2." Molecular Brain Research **1**(3): 271-280.

Noya, R. and A. Capurso (1999). "Decreased frequency of apolipoprotein E e4 allele from Northern to Southern Europe in Alzheimer's disease patients and centenarians." Neuroscience Letters **277**: 53-56.

O'Donnell, V. B., N. H. Schebb, G. L. Milne, M. P. Murphy, C. P. Thomas, D. Steinhilber, S. L. Gelhaus, H. Kühn, M. H. Gelb, P. J. Jakobsson, I. A. Blair, R. C. Murphy, B. A. Freeman, A. R. Brash and G. A. FitzGerald (2023). "Failure to apply standard limit-of-detection or limit-of-quantitation criteria to specialized pro-resolving mediator analysis incorrectly characterizes their presence in biological samples." Nat Commun **14**(1): 7172.

Oliveira, M. M. and E. Klann (2022). "eIF2-dependent translation initiation: Memory consolidation and disruption in Alzheimer's disease." Semin Cell Dev Biol **125**: 101-109.

Ophir, G., N. Amariglio, J. Jacob-Hirsch, R. Elkon, G. Rechavi and D. M. Michaelson (2005). "Apolipoprotein E4 enhances brain inflammation by modulation of the NF-kappaB signaling cascade." Neurobiol Dis **20**(3): 709-718.

Ophir, G., N. Amariglio, J. Jacob-Hirsch, R. Elkon, G. Rechavi and D. M. Michaelson (2005). "Apolipoprotein E4 enhances brain inflammation by modulation of the NF-κB signaling cascade." Neurobiology of disease **20**(3): 709-718.

Pacheco-Quinto, J., A. Herdt, C. B. Eckman and E. A. Eckman (2013). "Endothelin-converting enzymes and related metalloproteases in Alzheimer's disease." Journal of Alzheimer's Disease **33**(s1): S101-S110.

Perea, G., M. Navarrete and A. Araque (2009). "Tripartite synapses: astrocytes process and control synaptic information." Trends Neurosci **32**(8): 421-431.

Perry, G., A. Nunomura, K. Hirai, X. Zhu, M. Prez, J. Avila, R. J. Castellani, C. S. Atwood, G. Aliev and L. M. Sayre (2002). "Is oxidative damage the fundamental pathogenic mechanism of Alzheimer’s and other neurodegenerative diseases?" Free Radical Biology and Medicine **33**(11): 1475-1479.

Pham, T. and K. H. Cheng (2022). "Exploring the binding kinetics and behaviors of self-aggregated beta-amyloid oligomers to phase-separated lipid rafts with or without ganglioside-clusters." Biophys Chem **290**: 106874.

Phillips, M. C. (2014). "Apolipoprotein E isoforms and lipoprotein metabolism." IUBMB life **66**(9): 616-623.

Piccarducci, R., C. Giacomelli, M. S. Bertilacchi, A. Benito-Martinez, N. Di Giorgi, S. Daniele, G. Signore, S. Rocchiccioli, M. Vilar, L. Marchetti and C. Martini (2023). "Apolipoprotein E epsilon4 triggers neurotoxicity via cholesterol accumulation, acetylcholine dyshomeostasis, and PKCepsilon mislocalization in cholinergic neuronal cells." Biochim Biophys Acta Mol Basis Dis **1869**(7): 166793.

Piccinini, M., F. Scandroglio, S. Prioni, B. Buccinnà, N. Loberto, M. Aureli, V. Chigorno, E. Lupino, G. DeMarco and A. Lomartire (2010). "Deregulated sphingolipid metabolism and membrane organization in neurodegenerative disorders." Molecular neurobiology **41**: 314-340.

Plascencia-Villa, G. and G. Perry (2021). "Preventive and Therapeutic Strategies in Alzheimer's Disease: Focus on Oxidative Stress, Redox Metals, and Ferroptosis." Antioxid Redox Signal **34**(8): 591-610.

Pope, S. D. and R. Medzhitov (2018). "Emerging Principles of Gene Expression Programs and Their Regulation." Molecular Cell **71**(3): 389-397.

Power, R., J. M. Nolan, A. Prado-Cabrero, W. Roche, R. Coen, T. Power and R. Mulcahy (2022). "Omega-3 fatty acid, carotenoid and vitamin E supplementation improves working memory in older adults: A randomised clinical trial." Clin Nutr **41**(2): 405-414.

Qi, Y. Y., X. Heng, Z. Y. Yao, S. Y. Qu, P. Y. Ge, X. Zhao, S. J. Ni, R. Guo, N. Y. Yang, Q. C. Zhang and H. X. Zhu (2022). "Involvement of Huanglian Jiedu Decoction on Microglia with Abnormal Sphingolipid Metabolism in Alzheimer's Disease." Drug Des Devel Ther **16**: 931-950.

Randez-Gil, F., L. Bojunga, F. Estruch, J. Winderickx, M. Del Poeta and J. A. Prieto (2020). "Sphingolipids and Inositol Phosphates Regulate the Tau Protein Phosphorylation Status in Humanized Yeast." Front Cell Dev Biol **8**: 592159.

Rannikmäe, K., R. N. Kalaria, S. M. Greenberg, H. C. Chui, F. A. Schmitt, N. Samarasekera, R. A.-S. Salman and C. L. Sudlow (2014). "APOE associations with severe CAA-associated vasculopathic changes: collaborative meta-analysis." J Neurol Neurosurg Psychiatry **85**(3): 300-305.

Reichert, C. O., F. A. de Freitas, J. Sampaio-Silva, L. Rokita-Rosa, P. L. Barros, D. Levy and S. P. Bydlowski (2020). "Ferroptosis Mechanisms Involved in Neurodegenerative Diseases." Int J Mol Sci **21**(22).

Reitz, C. and R. Mayeux (2014). "Alzheimer disease: epidemiology, diagnostic criteria, risk factors and biomarkers." Biochemical pharmacology **88**(4): 640-651.

Roberts, B. R., T. M. Ryan, A. I. Bush, C. L. Masters and J. A. Duce (2012). "The role of metallobiology and amyloid‐β peptides in Alzheimer’s disease." Journal of neurochemistry **120**: 149-166.

Roberts, M. S., B. M. Magnusson, F. J. Burczynski and M. Weiss (2002). "Enterohepatic circulation: physiological, pharmacokinetic and clinical implications." Clin Pharmacokinet **41**(10): 751-790.

Rodriguez, G. A., L. M. Tai, M. J. LaDu and G. W. Rebeck (2014). "Human APOE4 increases microglia reactivity at Aβ plaques in a mouse model of Aβ deposition." Journal of neuroinflammation **11**(1): 111.

Rogaev, E., R. Sherrington, E. Rogaeva, G. Levesque, M. Ikeda, Y. Liang, H. Chi, C. Lin, K. Holman and T. Tsuda (1995). "Familial Alzheimer's disease in kindreds with missense mutations in a gene on chromosome 1 related to the Alzheimer's disease type 3 gene." Nature **376**(6543): 775.

Rogers, J. T. and D. K. Lahiri (2004). "Metal and inflammatory targets for Alzheimer's disease." Current drug targets **5**(6): 535-551.

Rohn, T. T. (2014). "Is apolipoprotein E4 an important risk factor for vascular dementia?" International journal of clinical and experimental pathology **7**(7): 3504.

Safieh, M., A. D. Korczyn and D. M. Michaelson (2019). "ApoE4: an emerging therapeutic target for Alzheimer’s disease." BMC medicine **17**(1): 64.

Saha, N., B. Stoll, F. Lang and D. Häussinger (1992). "Effect of anisotonic cell-volume modulation on glutathione-S-conjugate release, t-butylhydroperoxide metabolism and the pentose-phosphate shunt in perfused rat liver." Eur J Biochem **209**(1): 437-444.

Sanan, D. A., K. H. Weisgraber, S. J. Russell, R. W. Mahley, D. Huang, A. Saunders, D. Schmechel, T. Wisniewski, B. Frangione, A. D. Roses and et al. (1994). "Apolipoprotein E associates with beta amyloid peptide of Alzheimer's disease to form novel monofibrils. Isoform apoE4 associates more efficiently than apoE3." J Clin Invest **94**(2): 860-869.

Saroja, S. R., K. Gorbachev, T. Julia, A. M. Goate and A. C. Pereira (2022). "Astrocyte-secreted glypican-4 drives APOE4-dependent tau hyperphosphorylation." Proc Natl Acad Sci U S A **119**(34): e2108870119.

Sasmita, A. O. (2019). "Current viral-mediated gene transfer research for treatment of Alzheimer’s disease." Biotechnology and Genetic Engineering Reviews **35**(1): 26-45.

Saul, A. and O. Wirths (2017). "Endogenous Apolipoprotein E (ApoE) Fragmentation Is Linked to Amyloid Pathology in Transgenic Mouse Models of Alzheimer's Disease." Mol Neurobiol **54**(1): 319-327.

Savaskan, N. E., A. Borchert, A. U. Bräuer and H. Kuhn (2007). "Role for glutathione peroxidase-4 in brain development and neuronal apoptosis: specific induction of enzyme expression in reactive astrocytes following brain injury." Free Radical Biology and Medicine **43**(2): 191-201.

Schäfer, M., F. Reisch, D. Labuz, H. Machelska, S. Stehling, G. P. Püschel, M. Rothe, D. Heydeck and H. Kuhn (2023). "Humanization of the Reaction Specificity of Mouse Alox15b Inversely Modified the Susceptibility of Corresponding Knock-In Mice in Two Different Animal Inflammation Models." Int J Mol Sci **24**(13).

Schebb, N. H., H. Kühn, A. S. Kahnt, K. M. Rund, V. B. O'Donnell, N. Flamand, M. Peters-Golden, P. J. Jakobsson, K. H. Weylandt, N. Rohwer, R. C. Murphy, G. Geisslinger, G. A. FitzGerald, J. Hanson, C. Dahlgren, M. W. Alnouri, S. Offermanns and D. Steinhilber (2022). "Formation, Signaling and Occurrence of Specialized Pro-Resolving Lipid Mediators-What is the Evidence so far?" Front Pharmacol **13**: 838782.

Schwitter, C., B. Lutz and L. Bindila (2023). "Extraction and Simultaneous Quantification of Endocannabinoids and Endocannabinoid-Like Lipids in Biological Tissues." Methods Mol Biol **2576**: 9-19.

Selley, M., D. Close and S. Stern (2002). "The effect of increased concentrations of homocysteine on the concentration of (E)-4-hydroxy-2-nonenal in the plasma and cerebrospinal fluid of patients with Alzheimer’s disease." Neurobiology of aging **23**(3): 383-388.

Sen, A., D. L. Alkon and T. J. Nelson (2012). "Apolipoprotein E3 (apoE3) but not apoE4 protects against synaptic loss through increased expression of protein kinase Cϵ." Journal of Biological Chemistry **287**(19): 15947-15958.

Serrano-Pozo, A., S. Das and B. T. Hyman (2021). "APOE and Alzheimer's disease: advances in genetics, pathophysiology, and therapeutic approaches." Lancet Neurol **20**(1): 68-80.

Sharma, V. K., V. Mehta and T. G. Singh (2020). "Alzheimer's Disorder: Epigenetic Connection and Associated Risk Factors." Curr Neuropharmacol **18**(8): 740-753.

Sherrington, R., E. Rogaev, Y. a. Liang, E. Rogaeva, G. Levesque, M. Ikeda, H. Chi, C. Lin, G. Li and K. Holman (1995). "Cloning of a gene bearing missense mutations in early-onset familial Alzheimer's disease." Nature **375**(6534): 754.

Shin, R.-W., T. P. Kruck, H. Murayama and T. Kitamoto (2003). "A novel trivalent cation chelator Feralex dissociates binding of aluminum and iron associated with hyperphosphorylated τ of Alzheimer’s disease." Brain research **961**(1): 139-146.

Simopoulos, A. P. (2002). "The importance of the ratio of omega-6/omega-3 essential fatty acids." Biomedicine & pharmacotherapy **56**(8): 365-379.

Singh, N., S. Haldar, A. K. Tripathi, K. Horback, J. Wong, D. Sharma, A. Beserra, S. Suda, C. Anbalagan and S. Dev (2014). "Brain iron homeostasis: from molecular mechanisms to clinical significance and therapeutic opportunities." Antioxidants & redox signaling **20**(8): 1324-1363.

Singh, P., M. Singh and S. Mastana (2006). "APOE distribution in world populations with new data from India and the UK." Annals of human biology **33**(3): 279-308.

Smith, M. A., P. L. Harris, L. M. Sayre and G. Perry (1997). "Iron accumulation in Alzheimer disease is a source of redox-generated free radicals." Proceedings of the National Academy of Sciences **94**(18): 9866-9868.

Song, G., H. Tian, S. Qin, X. Sun, S. Yao, C. Zong, Y. Luo, J. Liu, Y. Yu, H. Sang and X. Wang (2012). "Hydrogen decreases athero-susceptibility in apolipoprotein B-containing lipoproteins and aorta of apolipoprotein E knockout mice." Atherosclerosis **221**(1): 55-65.

Spanbroek, R., M. Hildner, A. Köhler, A. Müller, F. Zintl, H. Kühn, O. Rådmark, B. Samuelsson and A. J. Habenicht (2001). "IL-4 determines eicosanoid formation in dendritic cells by down-regulation of 5-lipoxygenase and up-regulation of 15-lipoxygenase 1 expression." Proc Natl Acad Sci U S A **98**(9): 5152-5157.

Spite, M., L. V. Norling, L. Summers, R. Yang, D. Cooper, N. A. Petasis, R. J. Flower, M. Perretti and C. N. Serhan (2009). "Resolvin D2 is a potent regulator of leukocytes and controls microbial sepsis." Nature **461**(7268): 1287-1291.

Stockwell, B. R. (2022). "Ferroptosis turns 10: Emerging mechanisms, physiological functions, and therapeutic applications." Cell **185**(14): 2401-2421.

Sun, Y., X. Xia, D. Basnet, J. C. Zheng, J. Huang and J. Liu (2022). "Mechanisms of Ferroptosis and Emerging Links to the Pathology of Neurodegenerative Diseases." Front Aging Neurosci **14**: 904152.

Sun, Y. Y., Z. Wang and H. C. Huang (2023). "Roles of ApoE4 on the Pathogenesis in Alzheimer's Disease and the Potential Therapeutic Approaches." Cell Mol Neurobiol **43**(7): 3115-3136.

Tai, L. M., T. Bilousova, L. Jungbauer, S. K. Roeske, K. L. Youmans, C. Yu, W. W. Poon, L. B. Cornwell, C. A. Miller and H. V. Vinters (2013). "Levels of soluble apolipoprotein E/amyloid-β (Aβ) complex are reduced and oligomeric Aβ increased with APOE4 and Alzheimer disease in a transgenic mouse model and human samples." Journal of Biological Chemistry **288**(8): 5914-5926.

Tai, L. M., S. Ghura, K. P. Koster, V. Liakaite, M. Maienschein-Cline, P. Kanabar, N. Collins, M. Ben-Aissa, A. Z. Lei, N. Bahroos, S. J. Green, B. Hendrickson, L. J. Van Eldik and M. J. LaDu (2015). "APOE-modulated Aβ-induced neuroinflammation in Alzheimer's disease: current landscape, novel data, and future perspective." J Neurochem **133**(4): 465-488.

Tai, L. M., S. Mehra, V. Shete, S. Estus, G. W. Rebeck, G. Bu and M. J. LaDu (2014). "Soluble apoE/Abeta complex: mechanism and therapeutic target for APOE4-induced AD risk." Mol Neurodegener **9**: 2.

Tai, L. M., R. Thomas, F. M. Marottoli, K. P. Koster, T. Kanekiyo, A. W. Morris and G. Bu (2016). "The role of APOE in cerebrovascular dysfunction." Acta neuropathologica **131**(5): 709-723.

Tambini, M. D., M. Pera, E. Kanter, H. Yang, C. Guardia‐Laguarta, D. Holtzman, D. Sulzer, E. Area‐Gomez and E. A. Schon (2016). "ApoE4 upregulates the activity of mitochondria‐associated ER membranes." EMBO reports **17**(1): 27-36.

Tang, B., J. Zhu, J. Li, K. Fan, Y. Gao, S. Cheng, C. Kong, L. Zheng, F. Wu, Q. Weng, C. Lu and J. Ji (2020). "The ferroptosis and iron-metabolism signature robustly predicts clinical diagnosis, prognosis and immune microenvironment for hepatocellular carcinoma." Cell Commun Signal **18**(1): 174.

Tang, D., X. Chen, R. Kang and G. Kroemer (2021). "Ferroptosis: molecular mechanisms and health implications." Cell Res **31**(2): 107-125.

Tao, Q., T. F. A. Ang, C. DeCarli, S. H. Auerbach, S. Devine, T. D. Stein, X. Zhang, J. Massaro, R. Au and W. Q. Qiu (2018). "Association of Chronic Low-grade Inflammation With Risk of Alzheimer Disease in ApoE4 Carriers." JAMA Netw Open **1**(6): e183597.

Taylor, D. R. and N. M. Hooper (2007). "Role of lipid rafts in the processing of the pathogenic prion and Alzheimer's amyloid-beta proteins." Semin Cell Dev Biol **18**(5): 638-648.

Teng, Z. (2024). "Novel Development and Prospects in Pathogenesis, Diagnosis, and Therapy of Alzheimer's Disease." J Alzheimers Dis Rep **8**(1): 345-354.

Teter, B., M. J. LaDu, P. M. Sullivan, S. A. Frautschy and G. M. Cole (2016). "Apolipoprotein E isotype-dependent modulation of microRNA-146a in plasma and brain." Neuroreport **27**(11): 791.

Tjonahen, E., S. F. Oh, J. Siegelman, S. Elangovan, K. B. Percarpio, S. Hong, M. Arita and C. N. Serhan (2006). "Resolvin E2: identification and anti-inflammatory actions: pivotal role of human 5-lipoxygenase in resolvin E series biosynthesis." Chem Biol **13**(11): 1193-1202.

Tolar, M., J. N. Keller, S. Chan, M. P. Mattson, M. A. Marques and K. A. Crutcher (1999). "Truncated apolipoprotein E (ApoE) causes increased intracellular calcium and may mediate ApoE neurotoxicity." Journal of Neuroscience **19**(16): 7100-7110.

Troesch, B., M. Eggersdorfer, A. Laviano, Y. Rolland, A. D. Smith, I. Warnke, A. Weimann and P. C. Calder (2020). "Expert Opinion on Benefits of Long-Chain Omega-3 Fatty Acids (DHA and EPA) in Aging and Clinical Nutrition." Nutrients **12**(9).

Uddin, M. S., M. T. Kabir, A. Al Mamun, M. M. Abdel-Daim, G. E. Barreto and G. M. Ashraf (2019). "APOE and Alzheimer’s disease: evidence mounts that targeting APOE4 may combat Alzheimer’s pathogenesis." Molecular neurobiology **56**(4): 2450-2465.

Urquhart, P., A. Nicolaou and D. F. Woodward (2015). "Endocannabinoids and their oxygenation by cyclo-oxygenases, lipoxygenases and other oxygenases." Biochim Biophys Acta **1851**(4): 366-376.

Van Bergen, J., X. Li, J. Hua, S. Schreiner, S. Steininger, F. Quevenco, M. Wyss, A. Gietl, V. Treyer and S. Leh (2016). "Colocalization of cerebral iron with amyloid beta in mild cognitive impairment." Scientific reports **6**: 35514.

Vance, J. E. (2014). "MAM (mitochondria-associated membranes) in mammalian cells: lipids and beyond." Biochim Biophys Acta **1841**(4): 595-609.

Vejandla, B., S. Savani, R. Appalaneni, R. S. Veeravalli and S. S. Gude (2024). "Alzheimer's Disease: The Past, Present, and Future of a Globally Progressive Disease." Cureus **16**(1): e51705.

Vinothkumar, G., C. Kedharnath, S. Krishnakumar, S. Sreedhar, K. Preethikrishnan, S. Dinesh, A. Sundaram, D. Balakrishnan, G. Shivashekar, Sureshkumar and P. Venkataraman (2017). "Abnormal amyloid β(42) expression and increased oxidative stress in plasma of CKD patients with cognitive dysfunction: A small scale case control study comparison with Alzheimer's disease." BBA Clin **8**: 20-27.

Wang, F., J. Wang, Y. Shen, H. Li, W. D. Rausch and X. Huang (2022). "Iron Dyshomeostasis and Ferroptosis: A New Alzheimer's Disease Hypothesis?" Front Aging Neurosci **14**: 830569.

Wang, X. Y., H. Q. Li, Y. J. Sheng, B. Q. He, Z. Y. Liu, W. L. Li, S. J. Yu, J. J. Wang, Y. X. Zhang, J. Y. Chen, L. P. Qin and X. Y. Meng (2024). "The function of sphingolipids in different pathogenesis of Alzheimer's disease: A comprehensive review." Biomedicine & Pharmacotherapy **171**.

Wang, Y.-Q., S.-Y. Chang, Q. Wu, Y.-J. Gou, L. Jia, Y.-M. Cui, P. Yu, Z.-H. Shi, W.-S. Wu and G. Gao (2016). "The protective role of mitochondrial ferritin on erastin-induced ferroptosis." Frontiers in aging neuroscience **8**: 308.

Wang, Y.-Y., Y.-J. Ge, C.-C. Tan, X.-P. Cao, L. Tan and W. Xu (2021). "The proportion of APOE4 carriers among non-demented individuals: a pooled analysis of 389,000 community-dwellers." Journal of Alzheimer's Disease **81**(3): 1331-1339.

Ward, R. J., F. A. Zucca, J. H. Duyn, R. R. Crichton and L. Zecca (2014). "The role of iron in brain ageing and neurodegenerative disorders." The Lancet Neurology **13**(10): 1045-1060.

Weisgraber, K. H. (1994). "Apolipoprotein E: structure-function relationships." Advances in protein chemistry **45**: 249-302.

Williams, D. L., P. Dawson, T. Newman and L. Rudel (1985). "Apolipoprotein E synthesis in peripheral tissues of nonhuman primates." Journal of Biological Chemistry **260**(4): 2444-2451.

Wisniewski, T. and E. Drummond (2020). "APOE-amyloid interaction: Therapeutic targets." Neurobiology of Disease **138**.

Xian, X., T. Pohlkamp, M. S. Durakoglugil, C. H. Wong, J. K. Beck, C. Lane-Donovan, F. Plattner and J. Herz (2018). "Reversal of ApoE4-induced recycling block as a novel prevention approach for Alzheimer’s disease." Elife **7**: e40048.

Xu, P. T., J. R. Gilbert, H. L. Qiu, J. Ervin, T. R. Rothrock-Christian, C. Hulette and D. E. Schmechel (1999). "Specific regional transcription of apolipoprotein E in human brain neurons." Am J Pathol **154**(2): 601-611.

Xu, Q., A. Bernardo, D. Walker, T. Kanegawa, R. W. Mahley and Y. Huang (2006). "Profile and regulation of apolipoprotein E (ApoE) expression in the CNS in mice with targeting of green fluorescent protein gene to the ApoE locus." J Neurosci **26**(19): 4985-4994.

Xu, Q., D. Walker, A. Bernardo, J. Brodbeck, M. E. Balestra and Y. Huang (2008). "Intron-3 retention/splicing controls neuronal expression of apolipoprotein E in the CNS." J Neurosci **28**(6): 1452-1459.

Xu, X. (2009). "γ-Secretase Catalyzes Sequential Cleavages of the AβPP Transmembrane Domain." Journal of Alzheimer's disease: JAD **16**(2): 211.

Yamazaki, Y., N. Zhao, T. R. Caulfield, C. C. Liu and G. Bu (2019). "Apolipoprotein E and Alzheimer disease: pathobiology and targeting strategies." Nat Rev Neurol **15**(9): 501-518.

Yan, S. S., D. Chen, S. Yan, L. Guo and J. X. Chen (2012). "RAGE is a key cellular target for Aβ-induced perturbation in Alzheimer's disease." Frontiers in bioscience (Scholar edition) **4**: 240.

Yang, H., J. M. Zhuo, J. Chu, C. Chinnici and D. Praticò (2010). "Amelioration of the Alzheimer's disease phenotype by absence of 12/15-lipoxygenase." Biol Psychiatry **68**(10): 922-929.

Yang, T., Y. Zhang, L. Chen, E. R. Thomas, W. Yu, B. Cheng and X. Li (2023). "The potential roles of ATF family in the treatment of Alzheimer's disease." Biomed Pharmacother **161**: 114544.

Yang, W. S., R. SriRamaratnam, M. E. Welsch, K. Shimada, R. Skouta, V. S. Viswanathan, J. H. Cheah, P. A. Clemons, A. F. Shamji and C. B. Clish (2014). "Regulation of ferroptotic cancer cell death by GPX4." Cell **156**(1-2): 317-331.

Yang, W. S. and B. R. Stockwell (2008). "Synthetic lethal screening identifies compounds activating iron-dependent, nonapoptotic cell death in oncogenic-RAS-harboring cancer cells." Chemistry & biology **15**(3): 234-245.

Yang, W. S. and B. R. Stockwell (2016). "Ferroptosis: death by lipid peroxidation." Trends in cell biology **26**(3): 165-176.

Yao, Y., C. M. Clark, J. Q. Trojanowski, V. M. Lee and D. Praticò (2005). "Elevation of 12/15 lipoxygenase products in AD and mild cognitive impairment." Ann Neurol **58**(4): 623-626.

Yokoyama, M., H. Kobayashi, L. Tatsumi and T. Tomita (2022). "Mouse Models of Alzheimer's Disease." Front Mol Neurosci **15**: 912995.

Yoon, S.-S. and S. A. Jo (2012). "Mechanisms of amyloid-β peptide clearance: potential therapeutic targets for Alzheimer’s disease." Biomolecules & therapeutics **20**(3): 245.

Yu, Y., Y. Cheng, J. Fan, X. S. Chen, A. Klein-Szanto, G. A. Fitzgerald and C. D. Funk (2005). "Differential impact of prostaglandin H synthase 1 knockdown on platelets and parturition." J Clin Invest **115**(4): 986-995.

Zhang, L. and H. Hong (2015). "Genomic discoveries and personalized medicine in neurological diseases." Pharmaceutics **7**(4): 542-553.

Zhang, Y., M. Wang and W. Chang (2022). "Iron dyshomeostasis and ferroptosis in Alzheimer's disease: Molecular mechanisms of cell death and novel therapeutic drugs and targets for AD." Front Pharmacol **13**: 983623.

Zhang, Y.-w., R. Thompson, H. Zhang and H. Xu (2011). "APP processing in Alzheimer's disease." Molecular brain **4**(1): 3.

Zhang, Y.-w., R. Thompson, H. Zhang and H. Xu (2011). "APP processing in Alzheimer's disease." Molecular brain **4**: 1-13.

Zhao, D., K. Yang, H. Guo, J. Zeng, S. Wang, H. Xu, A. Ge, L. Zeng, S. Chen and J. Ge (2023). "Mechanisms of ferroptosis in Alzheimer's disease and therapeutic effects of natural plant products: A review." Biomed Pharmacother **164**: 114312.

Zheng, H. and E. H. Koo (2006). "The amyloid precursor protein: beyond amyloid." Molecular neurodegeneration **1**: 1-12.

Zhou, M., H. Zhao, X. Wang, J. Sun and J. Su (2019). "Analysis of long noncoding RNAs highlights region-specific altered expression patterns and diagnostic roles in Alzheimer's disease." Brief Bioinform **20**(2): 598-608.

Zhu, C. W. and M. Sano (2006). "Economic considerations in the management of Alzheimer's disease." Clin Interv Aging **1**(2): 143-154.
